# Genetic and hypoxic alterations of the microRNA-210-ISCU1/2 axis promote iron–sulfur deficiency and pulmonary hypertension

**DOI:** 10.15252/emmm.201404511

**Published:** 2015-03-30

**Authors:** Kevin White, Yu Lu, Sofia Annis, Andrew E Hale, B Nelson Chau, James E Dahlman, Craig Hemann, Alexander R Opotowsky, Sara O Vargas, Ivan Rosas, Mark A Perrella, Juan C Osorio, Kathleen J Haley, Brian B Graham, Rahul Kumar, Rajan Saggar, Rajeev Saggar, W Dean Wallace, David J Ross, Omar F Khan, Andrew Bader, Bernadette R Gochuico, Majed Matar, Kevin Polach, Nicolai M Johannessen, Haydn M Prosser, Daniel G Anderson, Robert Langer, Jay L Zweier, Laurence A Bindoff, David Systrom, Aaron B Waxman, Richard C Jin, Stephen Y Chan

**Affiliations:** 1 Divisions of Cardiovascular Medicine and Network Medicine, Department of Medicine, Brigham and Women's Hospital, Harvard Medical SchoolBoston, MA, USA; 2 Regulus TherapeuticsSan Diego, CA, USA; 3 Institute for Medical Engineering and Science, Massachusetts Institute of TechnologyCambridge, MA, USA; 4 Harvard-MIT Division of Health Sciences and Technology, Massachusetts Institute of TechnologyCambridge, MA, USA; 5 Department of Chemical Engineering, Massachusetts Institute of TechnologyCambridge, MA, USA; 6 David H. Koch Institute for Integrative Cancer Research, Massachusetts Institute of TechnologyCambridge, MA, USA; 7 The Davis Heart and Lung Research Institute, Division of Cardiovascular Medicine, Department of Internal Medicine, Wexner Medical Center, The Ohio State UniversityColumbus, OH, USA; 8 Department of Cardiology, Boston Children's Hospital, Harvard Medical SchoolBoston, MA, USA; 9 Department of Pathology, Boston Children's Hospital, Harvard Medical SchoolBoston, MA, USA; 10 Division of Pulmonary/Critical Care Medicine, Department of Medicine, Harvard Medical SchoolBoston, MA, USA; 11 Department of Pediatric Newborn Medicine, Brigham and Women's Hospital, Harvard Medical SchoolBoston, MA, USA; 12 Program in Translational Lung Research, University of ColoradoDenver, Aurora, CO, USA; 13 Departments of Medicine and Pathology, David Geffen School of Medicine, University of California, Los AngelesLos Angeles, CA, USA; 14 Department of Cardiothoracic Surgery, University of Arizona College of MedicinePhoenix, AZ, USA; 15 Medical Genetics Branch, National Human Genome Research Institute, National Institutes of HealthBethesda, MD, USA; 16 Celsion-EGEN, Inc.Huntsville, AL, USA; 17 Department of Cardiology, University of BergenBergen, Norway; 18 The Wellcome Trust Sanger Institute, Wellcome Trust Genome CampusHinxton, Cambridge, UK; 19 Department of Clinical Medicine, University of BergenBergen, Norway; 20 Department of Neurology, Haukeland University HospitalBergen, Norway

**Keywords:** endothelial, iron–sulfur, metabolism, microRNA, mitochondria

## Abstract

Iron–sulfur (Fe-S) clusters are essential for mitochondrial metabolism, but their regulation in pulmonary hypertension (PH) remains enigmatic. We demonstrate that alterations of the miR-210-ISCU1/2 axis cause Fe-S deficiencies *in vivo* and promote PH. In pulmonary vascular cells and particularly endothelium, hypoxic induction of miR-210 and repression of the miR-210 targets ISCU1/2 down-regulated Fe-S levels. In mouse and human vascular and endothelial tissue affected by PH, miR-210 was elevated accompanied by decreased ISCU1/2 and Fe-S integrity. In mice, miR-210 repressed ISCU1/2 and promoted PH. Mice deficient in miR-210, via genetic/pharmacologic means or via an endothelial-specific manner, displayed increased ISCU1/2 and were resistant to Fe-S-dependent pathophenotypes and PH. Similar to hypoxia or miR-210 overexpression, ISCU1/2 knockdown also promoted PH. Finally, cardiopulmonary exercise testing of a woman with homozygous *ISCU* mutations revealed exercise-induced pulmonary vascular dysfunction. Thus, driven by acquired (hypoxia) or genetic causes, the miR-210-ISCU1/2 regulatory axis is a pathogenic lynchpin causing Fe-S deficiency and PH. These findings carry broad translational implications for defining the metabolic origins of PH and potentially other metabolic diseases sharing similar underpinnings.

See also: H Tang et al (June 2015)

## Introduction

Iron–sulfur (Fe-S) clusters ([4Fe-4S] and [2Fe-2S]) are critical bioinorganic prosthetic groups that are essential for electron transport and consequent metabolic processes (Beinert *et al*, 1997). The formation of Fe-S clusters is controlled by a conserved set of assembly and scaffold proteins. Current knowledge regarding the importance of these proteins in human disease has been derived largely through investigation of genetic mutations (as reviewed by Rouault, 2012). Yet, stemming from the relatively rare occurrence of these genetic mutations and technical obstacles to measuring these prosthetic groups in mammals, the regulation and actions of Fe-S clusters in the wide spectrum of human metabolic disease have been poorly investigated.

Pulmonary hypertension (PH) is a deadly and increasingly prevalent vascular disease where Fe-S biology may figure prominently. PH is defined by increased pulmonary arterial pressure and lung vasculopathy, triggered by varied and often disparate stimuli (Schermuly *et al*, 2011). Among these triggers, hypoxia and the actions of its master transcription factors of hypoxia, HIF-1α and HIF-2α, are well-recognized insults in multiple PH subtypes, including pulmonary arterial hypertension (PAH, WHO Group 1) (Bonnet *et al*, 2006; Fijalkowska *et al*, 2010; Farha *et al*, 2011; Marsboom *et al*, 2012) as well as PH associated with hypoxic lung diseases (WHO Group 3) (as reviewed by Tuder *et al*, 2013). The pulmonary vascular response to hypoxia and HIF factors in PH is incompletely characterized but increasingly has been linked to chronic repression of mitochondrial metabolism (Cottrill & Chan, 2013). Some factors have been identified to modulate metabolic processes in the diseased pulmonary vasculature, but the entire complement of regulators remains undefined.

Studying cultured cells, we previously reported that hypoxia up-regulated the HIF-α-dependent microRNA-210 (miR-210), leading to specific mitochondrial and metabolic alterations (Chan *et al*, 2009). In hypoxia, this microRNA (miRNA) decreased expression of its targets ISCU1 and ISCU2 (iron–sulfur cluster assembly proteins 1/2 or typically described by the single term, ISCU1/2). In mammalian cells, two splice isoforms of ISCU exist and both serve as scaffolding proteins essential for the biogenesis of Fe-S clusters. Both transcripts carry an identical 3′ UTR, but they differ in their location: ISCU1 is located in the cytosol, whereas ISCU2 is located in the mitochondria (Tong & Rouault, 2000). We found that down-regulation of ISCU1/2 decreased Fe-S-dependent mitochondrial respiration and promoted a metabolic shift toward glycolysis for energy production. In the acute setting, this adaptive metabolic shift improved cell survival. However, the effects in health or disease of chronic activation of the miR-210-ISCU1/2 axis with consequent repression of Fe-S clusters *in vivo* are not known. Based on the pathologic consequences of metabolic dysfunction in other diseases such as cancer, we hypothesized that chronic repression of Fe-S biogenesis directly drives dysfunction of mitochondrial metabolism, cellular proliferation, and frank disease. By interrogating that model further in both acquired injury (e.g. hypoxia) and genetic human disease (*ISCU mut/mut*), we now define the miR-210-ISCU1/2 regulatory axis as a crucial pathogenic lynchpin of pulmonary vascular disease *in vivo*.

## Results

### The miR-210-ISCU1/2 regulatory axis is activated in PH related to hypoxia and HIF activity

**Figure 1:**
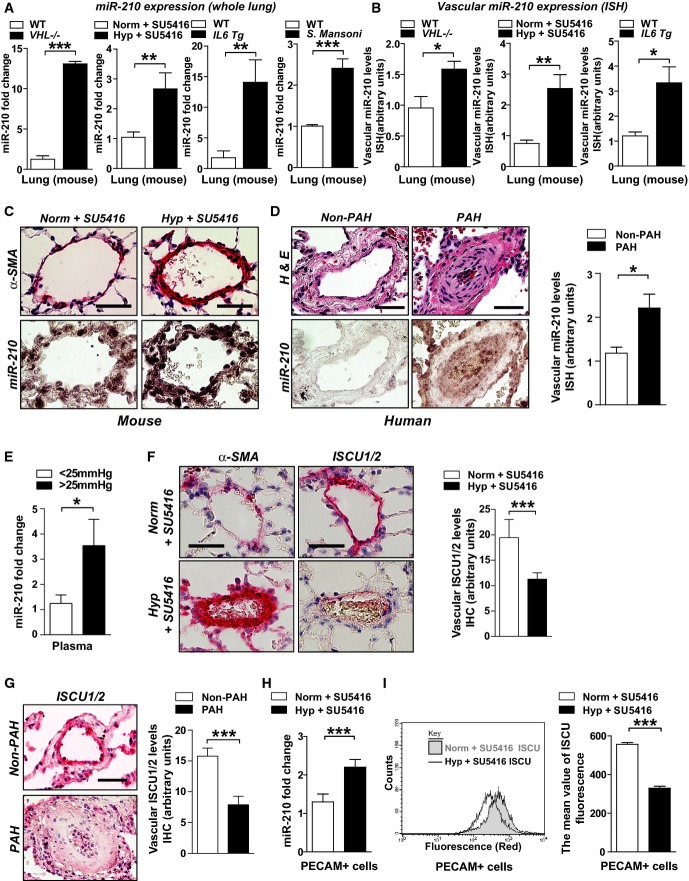
The miR-210-ISCU1/2 regulatory axis is activated in endothelium and remodeled vessels in PH

Based on our prior studies of the regulation of miR-210 in pulmonary arterial endothelial cells (PAECs) (Chan *et al*, 2009) and the up-regulation of both HIF-1α and HIF-2α in vascular endothelial cells in hypoxia-relevant PH in mice (Supplementary Fig S1), we hypothesized that the miR-210-ISCU1/2 axis is active in various forms of PH stemming from hypoxia-dependent or HIF-dependent activity. By RT–PCR, we found that miR-210 was induced in the lungs of mice deficient in the von Hippel–Lindau gene (*VHL*^−/−^), a genetic model of PH driven by constitutive HIF-α activation (Fig1A, Supplementary Fig S2A). miR-210 was also up-regulated in lungs of mice suffering from PH stemming from chronic hypoxia (10% O_2_) (Supplementary Fig S2B) and in mouse lungs with more severe PH (Supplementary Fig S2A) stemming from chronic hypoxia accompanied by serial administration of the VEGF-receptor antagonist SU5416 (Fig1A). Corresponding with the link between inflammatory cytokine stimulation and up-regulated HIF activity, miR-210 was also induced in inflammatory mouse models of PH driven by transgenic pulmonary interleukin-6 (IL-6) expression [elevated right ventricular systolic pressures reported in Steiner *et al* (2009)] and by chronic *S. mansoni* infection (Fig1A, Supplementary Fig S2A). In correlation, *in situ* miRNA staining was performed using specific mouse models where reliable histologic assessment of pulmonary vascular cell types was possible. Such staining revealed that miR-210 expression was induced within the diseased pulmonary vasculature of mice (< 100 μm external diameter vessels) (Fig1B and C), as compared with non-diseased tissue and miR-scrambled control probe (Supplementary Fig S3A). Induction of miR-210 was observed in remodeled pulmonary vessels (Fig1B) but not in other peripheral vascular tissue (Supplementary Fig S4A). MiR-210 expression was also up-regulated in the small diseased pulmonary arterioles (< 200 μm external diameter) in human PAH lung tissues compared with those arterioles observed in non-diseased human lung tissue (Fig1D; patient demographics in Supplementary Table S1). Among other vascular cell types, staining for miR-210 was also evident in the intimal layer of remodeled vessels, consistent with prior studies implicating the robust actions of miR-210 in PAECs (Chan *et al*, 2009). Circulating, extracellular miR-210 was also significantly elevated in plasma sampled adjacent to the pulmonary vascular space (pulmonary capillary wedge position, PCWP) from PH individuals (mean pulmonary arterial pressures, mPAP ≥ 25 mmHg, PCWP ≤ 15 mmHg) as compared with PH-free individuals (mPAP < 25 mmHg, Fig1E, demographic information in Supplementary Table S2). Such findings are consistent with a growing literature reporting that diseased tissues, which express increased levels of specific miRNAs, can release those miRNAs into the extracellular space and into the circulating plasma (Creemers *et al*, 2012). Conversely, the direct miR-210 targets ISCU1/2, detected specifically by immunohistochemical stain (Supplementary Fig S3B), were down-regulated in diseased pulmonary vasculature of mice and humans (Fig1F and G) suffering from PH, but not in unaffected peripheral vasculature (Supplementary Fig S4B–D).

A RT–qPCR reveals that miR-210 was increased in lungs of mice with PH triggered by various conditions: *VHL*^−/−^ as compared with *VHL*^+/+^ mice (*N* = 4/group), ***P <* *0.0001 (first graph); hypoxia + SU5416 (Hyp + SU5416) (*N* = 6/group) as compared with normoxia + SU5416 (Norm + SU5416) (*N* = 7/group), **P = 0.0015 (second graph); *Il6* transgenic versus littermate control mice (*N* = 4/group), **P = 0.0097 (third graph); and *S. mansoni*-infected mice (*N* = 4) compared with non-infected control mice (*N* = 5), ***P < 0.0001 (fourth graph).

B From animal subjects in (A), *in situ* hybridization (ISH, purple stain) revealed increased miR-210 in < 100-μm pulmonary vessels of mice suffering from PH, *P = 0.0493 for first graph, **P = 0.0015 for second graph, *P = 0.0391 for third graph.

C Representative ISH stain of miR-210 in < 100 μm pulmonary vessels of mice (bottom micrographs) exposed to Hyp + SU5416 compared with Norm + SU5416 (α-smooth muscle actin stain from serial sections, top row of micrographs).

D Increased miR-210 in < 200-μm remodeled pulmonary vessels of patients suffering from PAH (*N* = 19, Supplementary Table S1) as compared with non-PAH donor control lung (*N* = 10). Serial staining with hematoxylin and eosin is displayed in the top row of micrographs; quantification of miRNA ISH, right graph, *P = 0.0167.

E Increased levels of miR-210 in plasma drawn from the pulmonary circulation (pulmonary capillary wedge position, PCWP) of patients with elevated mean pulmonary arterial pressures (mean PAP ≥ 25 mmHg) compared with control subjects (mPAP < 25 mmHg, *N* = 5/group, demographics in Supplementary Table S2), *P = 0.0357.

F, G From animals in (C) and humans in (D), immunohistochemistry (IHC) revealed that the miR-210 targets ISCU1/2 were reciprocally down-regulated in miR-210-enriched remodeled pulmonary vessels—namely in PH mice exposed to Hyp + SU5416 (F, ***P = 0.0002) and in human PAH patients (G, ***P = 0.0008).

H, I miR-210 expression (H) was increased (***P = 0.0003), and ISCU1/2 expression (I) was decreased (***P < 0.0001) in PECAM^+^ pulmonary vascular endothelial cells isolated from PH mice (Hyp + SU5416) as compared with control (Norm + SU5416) (*N* = 4/group, left bars).

Data information: In (A, B), mean expression of miR-210 in control groups was assigned a fold change of 1, to which all samples were compared. Error bars reflect SEM. Mouse tissue scale bar: 50 μm, human tissue scale bar: 100 μm.

Guided by the intimal expression of miR-210, we wanted to quantify specifically the expression of miR-210 and ISCU1/2 in diseased pulmonary vascular endothelial cells. To do so, a MACS-based cellular sorting system was used to purify PECAM-positive (PECAM^+^) pulmonary vascular endothelial cells from PH mice exposed to hypoxia + SU5416 or hypoxia alone. These PECAM^+^ cells (> 95% purity, Supplementary Fig S5) displayed an up-regulation of miR-210 by RT–PCR (Fig1H, Supplementary Fig S6A) and a down-regulation of ISCU1/2 (by antibody staining and flow cytometry, Fig1I, Supplementary Fig S6B). In contrast, the expression of other reported targets of miR-210, such as SDHD, COX10, E2F3, and Ephrin A3, was unchanged or even increased in diseased PECAM^+^ cells from hypoxic mice as assessed by flow cytometry (Supplementary Fig S6C–F) or immunohistochemical stain of remodeled pulmonary vessels (Supplementary Fig S7A and B). Such findings emphasize the unique importance of ISCU1/2 as a canonical miR-210 target gene in this context. Furthermore, taken together, we can conclude that, in both mouse models and human examples of PH *in vivo*, the miR-210-ISCU1/2 regulatory axis is activated in small diseased pulmonary vessels, particularly in endothelial cells.

### Fe-S integrity is decreased in hypoxic pulmonary vascular cells and in hypoxic PH *in vivo*

**Figure 2:**
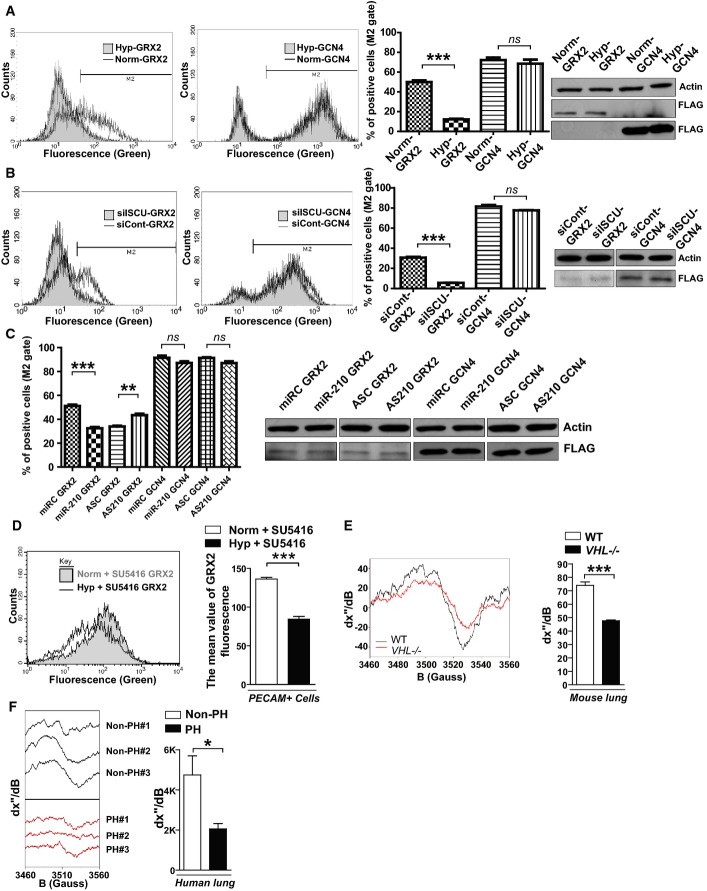
Impaired Fe-S cluster integrity in diseased pulmonary vasculature is driven by the miR-210-ISCU1/2 axis

Previously, we postulated that the down-regulation of Fe-S levels in PAECs is directly mediated by miR-210 and ISCU1/2 (Chan *et al*, 2009). To establish this principle, we optimized a previously validated fluorescent detection system of intracellular [2Fe-2S] clusters (Hoff *et al*, 2009). Specifically, this system utilizes two fusion proteins: one carrying the N-terminal half of the Venus fluorescent protein fused to the glutaredoxin 2 (GRX2) protein and another carrying the Venus C-terminal half fused to GRX2. When expressed in the same cell, these fusion constructs only fluoresce after GRX2 homodimerization—a process quantitatively dependent upon intact [2Fe-2S] clusters. After lentiviral delivery of sensor genes to cultured human PAECs, mean fluorescence was measured by flow cytometry of live cells, and sensor expression was quantified by immunoblot. GCN4 control sensors—fusion proteins that homodimerize independent of Fe-S levels—induced consistent sensor expression and fluorescence in all conditions (Fig2A–C). Similarly, GRX2 sensor expression was consistent among relevant comparisons. Yet, fluorescence derived from the GRX2 sensors decreased in hypoxia (Fig2A), reflecting a down-regulation of Fe-S levels. Supporting the role of the miR-210-ISCU1/2 axis in such Fe-S down-regulation, Fe-S-dependent GRX2 sensor fluorescence was also decreased in the absence of hypoxia by small interfering RNA (siRNA) knockdown of ISCU1/2 (Fig2B, efficiency of knockdown as demonstrated in Supplementary Fig S8A) or separately, by forced miR-210 expression [Fig2C, expression as we previously reported (Chan *et al*, 2009)]. Demonstrating the essential role of miR-210 in this hypoxic response, antisense inhibition of miR-210 partially rescued GRX2 fluorescence in the presence of hypoxia (Fig2C). Importantly, the miR-210-ISCU1/2 axis similarly regulated Fe-S levels in endothelial cells derived throughout the pulmonary vascular tree (Supplementary Fig S9). Such alterations of Fe-S levels were also not dependent upon reduction of total mitochondrial content in cultured pulmonary vascular cell types exposed to hypoxia (Supplementary Fig S10A and B), in mice exposed to hypoxia + SU5416 (Supplementary Fig S10C), or in mice where miR-210/ISCU1/2 were directly manipulated (Supplementary Fig S10D and E). Moreover, changes in Fe-S expression were also not dependent on total pulmonary iron content (Supplementary Fig S10F). Therefore, activation of miR-210 and down-regulation of ISCU1/2 are sufficient for repressing Fe-S levels in endothelial cells throughout the human pulmonary vascular tree and are necessary to do so during hypoxia.

After lentiviral delivery of GCN4 or GRX2 sensor genes to human PAECs, cellular fluorescence was measured by flow cytometry. Unlike control GCN4 sensors that homodimerized independent of Fe-S levels and induced consistent fluorescence, fluorescence derived from the GRX2 Fe-S-dependent sensors decreased in hypoxia, as displayed in representative flow cytometric plots (left) and by quantification of percentage of positive cells [(cell number in M2 gate)/(total cell number) × 100]. Immunoblotting revealed consistent expression of either GRX2 or GCN4 sensors (FLAG-tagged) in hypoxia (Hyp) compared with normoxia (Norm) (*N* = 3, ***P < 0.0001 for GRX2; *N* = 3, NS *P* = 0.1848 for GCN4).

In contrast to consistent fluorescence from control GCN4 sensors, Fe-S-dependent GRX2 sensor fluorescence was decreased by siRNA knockdown of ISCU1/2 (siISCU) as compared with control (siCont) (*N* = 3, ***P < 0.0001 for GRX2; *N* = 3, NS *P* = 0.1790 for GCN4).

GRX2, but not GCN4, sensor fluorescence was decreased after transfection of miR-210 oligonucleotide mimic (miR-210) as compared with control (miRC) (*N* = 3, ***P = 0.0002 for GRX2; *N* = 3, NS *P* = 0.0913 for GCN4). During hypoxic exposure, GRX2, but not GCN4, sensor fluorescence was increased after transfection of an antisense miR-210 inhibitor (AS210) as compared with control (ASC) (*N* = 3, **P = 0.003 for GRX2; *N* = 3, NS *P* = 0.1194 for GCN4).

After lentiviral delivery of sensor genes, Fe-S-dependent GRX2 sensor fluorescence was decreased in PECAM-positive cells from PH mouse lung (Hyp + SU5416) as compared with control non-PH mouse lung (Norm + SU5416, *N* = 3, ***P < 0.001).

By electron paramagnetic resonance (EPR) spectroscopy (representative *VHL*^−/−^ versus control lung), Fe-S cluster signal was decreased (left graph) in *VHL*^−/−^ mice lung tissue (*VHL*^−/−^, *N* = 3) as compared with WT control lung tissues (WT, *N* = 5), ***P = 0.0003.

By EPR, Fe-S-specific signal was decreased (right graph) in human PH-diseased lung tissue harvested at lung transplantation (PH, *N* = 3, Supplementary Table S3) compared with non-PH control donor lung tissues (control, *N* = 3), *P = 0.048.

Data information: In (A–C), sensor expression was confirmed by immunoblot for FLAG epitopes. Error bars reflect SEM. Source data are available online for this figure.

To determine whether Fe-S cluster biogenesis is repressed in PH *in vivo*, Fe-S-dependent GRX2 sensors as well as electron paramagnetic resonance (EPR) spectroscopy were utilized to quantitatively analyze Fe-S cluster levels in PH-diseased lung tissue. First, in PECAM^+^ cells derived from PH mice exposed to hypoxia + SU5416, a down-regulation of Fe-S-specific fluorescent signal was observed after lentiviral transduction of GRX2 sensors but not with GCN5 control sensors (Fig2D). In corroboration, as demonstrated by the alteration in EPR peak-to-peak spectroscopic signal lungs from *VHL*^−/−^ mice, Fe-S cluster levels were significantly down-regulated in PH-diseased *VHL*^−/−^ lung as compared with non-diseased littermate *VHL*^+/+^ control lung (Fig2E). Finally, Fe-S cluster levels were decreased in human lung tissue derived from PH patients undergoing lung transplantation (*N* = 3, patient demographics in Supplementary Table S3) as compared with donor tissue derived from non-PH persons (*N* = 3) (Fig2F). Taken together, these data demonstrate that deficiencies in Fe-S integrity, mediated in large part by hypoxia and the miR-210-ISCU1/2 axis, are prevalent in PH and may play a fundamental role in controlling pulmonary vascular homeostasis and disease.

### Hypoxic induction of miR-210 induces oxidative stress and pulmonary vascular proliferation via repression of Fe-S biogenesis and mitochondrial metabolism

**Figure 3:**
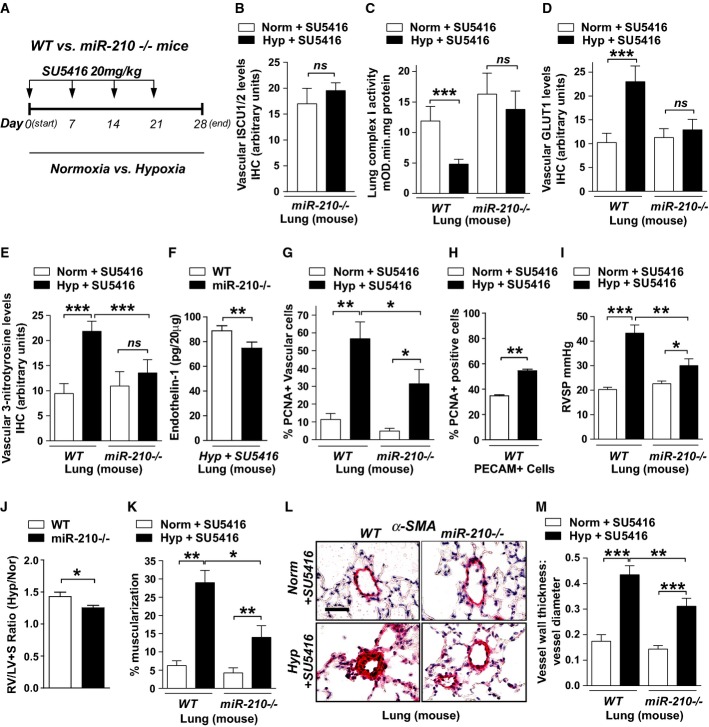
miR-210 regulates Fe-S biogenesis, mitochondrial function, and downstream PH pathways *in vivo* and is necessary to induce hypoxic PH in mice

To delineate the metabolic actions of miR-210 *in vivo* (Fig3A), we analyzed mice carrying homozygous deletions of the *mmu-miR-210* gene (*miR-210*^−/−^) as described (Prosser *et al*, 2011; Mok *et al*, 2013). Despite absent expression of miR-210 (Supplementary Fig S11), mice were viable at baseline and displayed no grossly abnormal phenotype. However, in contrast to decreased ISCU1/2 in diseased pulmonary vessels of WT mice exposed to hypoxia + SU5416, *miR-210*^−/−^ mice displayed preserved ISCU1/2 expression in small pulmonary vessels (< 100 μm) under those same conditions (Fig3B). Consistent with the role for ISCU1/2 and Fe-S biogenesis in mitochondrial electron transport in cultured cells (Chan *et al*, 2009), mitochondrial respiratory complex activity (Complex I) was also preserved in pulmonary tissue harvested from *miR-210*^−/−^ mice as compared with decreased activity in diseased WT mice exposed to hypoxia + SU5416 (Fig3C). Correspondingly, despite increased pulmonary vascular expression of the HIF-responsive glucose transporter-1 (GLUT1) in diseased WT mice (Fig3D), GLUT1 in *miR-210*^−/−^ pulmonary vessels was unchanged. Thus, by delineating a consistent reliance on mitochondrial metabolism in *miR-210*^−/−^ mice even under PH disease conditions, these findings demonstrated that miR-210 is necessary, at least in part, for the metabolic dysregulation observed in diseased pulmonary vasculature *in vivo*.

A Schema of comparing *miR-210*^−/−^ and WT mice after hypoxia + SU5416 (PH) versus normoxia + SU5416 (control).

B By immunohistochemistry (IHC), ISCU1/2 was unchanged in < 100 μm pulmonary vessels of *miR-210*^−/−^ mice exposed to Hyp + SU5416 (*N* = 6) versus Norm + SU5416 (*N* = 7), NS *P* = 0.2833.

C Fe-S-dependent Complex I-specific activity was decreased in WT PH mice as compared with control (*N* = 7/group, left bars, ***P < 0.0001), but activity was preserved in the lungs of *miR-210*^−/−^ mice in either condition (*N* = 8/group, right bars), NS *P* = 0.3693.

D IHC demonstrated that pulmonary vascular GLUT1 was increased in WT PH mice (left bars, ***P = 0.0006), but GLUT1 was unchanged in *miR-210*^−/−^ mice in either condition (right bars, NS *P* = 0.9967).

E 3-nitrotyrosine (3-NT) was increased in pulmonary vessels of WT PH mice but was reduced in *miR-210*^−/−^ mice in either condition (*N* = 6/group) (***P < 0.0001, NS *P* = 0.4087).

F In PH conditions, endothelin-1 was decreased in *miR-210*^−/−^ lung tissue compared with WT tissue (*N* = 5/group), **P = 0.007.

G PCNA was increased in WT PH pulmonary vessels but was decreased in *miR-210*^−/−^ tissue exposed to either condition (*N* = 5/group) (**P = 0.0096, *P = 0.0124, *P = 0.0263 for *miR-210*^−/−^*)*.

H PCNA was increased in PECAM^+^ endothelial cells from PH versus non-PH mice (*N* = 3/group, **P = 0.0052).

I Unlike WT mice (black bars) demonstrating increased right ventricular systolic pressure (RVSP) after Hyp + SU5416 (*N* = 10) versus Norm + SU5416 (*N* = 11), hemodynamic dysregulation was significantly alleviated in *miR-210*^−/−^ mice (white bars, *N* = 11) (***P < 0.0001, *P = 0.0258, **P = 0.0059).

J Compared with WT controls (*N* = 9), *miR-210*^−/−^ mice (*N* = 8) displayed a blunted increase of the Fulton index (RV/LV + S) under PH versus baseline conditions (expressed as a ratio of RV/LV + S under Hyp + SU5416 versus Norm + SU5416, *P = 0.031).

K–M Under PH (black bars, *N* = 8/group) versus baseline conditions (white bars, *N* = 6/group), pulmonary vascular remodeling was alleviated in *miR-210*^−/−^ mice, as visualized via histology (L), and confirmed by decreased % arteriolar muscularization (K, **P = 0.001, **P = 0.0045 for *miR-210*^−/−^, *P = 0.0158) and decreased vessel wall thickness (M, ***P < 0.0001, **P = 0.0086).

Data information: Error bars reflect SEM. Mouse tissue scale bar: 50 μm.

Next, we wanted to determine whether this miR-210-dependent metabolic shift is associated with phenotypes consistent with the cellular manifestations of PH. Expression levels of 3-nitrotyrosine, a marker of oxidative stress in the pulmonary vasculature, were significantly reduced in the pulmonary vessels of *miR-210*^−/−^ mice as compared with WT controls following hypoxia + SU5416 (Fig3E). Under the same conditions, expression of the potent vasoconstrictor endothelin-1 was also decreased in *miR-210*^−/−^ pulmonary tissue (Fig3F), consistent with alterations of endothelin-1 in cultured PAECs by miR-210 (Supplementary Fig S12). Finally, actively proliferating PCNA-positive pulmonary vascular cells were increased in WT controls exposed to hypoxia + SU5416, as quantified by immunofluorescent microscopy (Fig3G). Consistent with the activation of the miR-210-ISCU1/2 axis in vascular endothelial cells, based on flow cytometric analysis, such PCNA up-regulation corresponded specifically to PECAM^+^ pulmonary vascular endothelial cells (Fig3H). In contrast, PCNA expression was reduced in the *miR-210*^−/−^ pulmonary vascular wall compared with WT controls (Fig3G). Thus, we conclude that chronic induction of miR-210 *in vivo* represses ISCU1/2 as a primary target, leading to disruption of Fe-S biogenesis, mitochondrial metabolism, and pathologic alteration of the proliferative and oxidative states of the pulmonary vasculature.

### Pulmonary vascular induction of miR-210, particularly in the endothelium, promotes PH

Given the altered metabolic and cellular phenotypes driven by miR-210, we wanted to determine whether chronic induction of this miRNA is necessary and sufficient to promote PH. With hypoxia + SU5416, *miR-210*^−/−^ mice were substantially protected against the development of PH, exhibiting only slight increases in RVSP as compared with more substantial elevations in WT control mice (Fig3I). By echocardiography, left ventricular dysfunction was exonerated as a cause for PH (Supplementary Fig S13A). Yet, compared with WT controls, *miR-210*^−/−^ mice displayed a blunted increase of RV/LV + S (Fulton index) under disease versus baseline conditions, thus indicating at least partial protection from the RV hypertrophic response and consistent with the hemodynamic improvement (Fig3J). Furthermore, distal pulmonary vessel remodeling was significantly alleviated in *miR-210*^−/−^ mice compared with diseased WT littermates (Fig3K–M). Thus, miR-210 is necessary for the hypoxic induction of PH *in vivo*.

**Figure 4:**
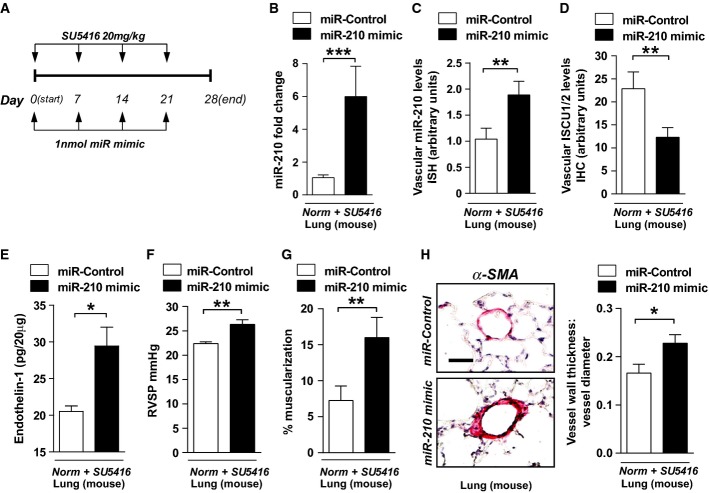
MiR-210 is sufficient to induce pulmonary vascular dysfunction in mice

To establish more definitively the causative actions of miR-210 and ISCU1/2 in PH, we next employed a variety of protocols for pharmacologic administration of miR-210 oligonucleotide mimics or inhibitors as well as a siRNA specific for ISCU1/2 in the pulmonary vasculature of mice. To do so, the time of hypoxic exposure and pharmacologic treatments were tailored to each type of condition and delivery system, in order to minimize any possible confounding effects of systemic overload of siRNA or miRNA oligonucleotides. First, in the presence of SU5416 but in the absence of hypoxia, chronic pulmonary expression of miR-210 in WT mice was achieved by serial intrapharyngeal injections of liposomally encapsulated miR-210 oligonucleotide mimics, as adapted from prior protocols (Bertero *et al*, 2014) (Fig4A). Delivery resulted in miR-210 up-regulation in whole lung tissue (Fig4B) and in small pulmonary vessels (Fig4C) but not the heart or other organs (Supplementary Fig S14). Such localized pulmonary delivery led to repression of pulmonary vascular ISCU1/2 (Fig4D, Supplementary Fig S7E and F). Interestingly, levels of other reported miR-210 targets such E2F3 and Ephrin A3 (Supplementary Fig S7C and D) were unchanged, again indicating the importance of ISCU1/2 in the specific actions of miR-210 in this context. Consistent with findings in cultured PAECs (Supplementary Fig S12A), endothelin-1 in pulmonary tissue was also up-regulated by miR-210 (Fig4E). In turn, chronic miR-210 expression led to elevated right ventricular systolic pressure (RVSP) compared with WT littermates treated with miR-Control (Fig4F). MiR-210 also induced pulmonary vascular remodeling, as demonstrated by substantial muscularization and medial thickening in the distal pulmonary vessels (Fig4G and H). Thus, even in the absence of hypoxia, miR-210 induction is sufficient to induce pulmonary vascular dysfunction *in vivo*.

A Schema for forced miR-210 expression *in vivo* (*N* = 8/group).

B, C Intrapharyngeal delivery of miR-210 mimic increased miR-210 in whole lung (***P = 0.0002) (B) and in < 100-μm pulmonary vessels (**P = 0.007) (C).

D miR-210 mimic also repressed ISCU1/2 levels in those same caliber vessels (*N* = 6/group), **P = 0.0022.

E Endothelin-1 was increased in mouse lung tissue after delivery of miR-210 mimic (*N* = 6/group), *P = 0.0368.

F–H miR-210 mimic delivery increased RVSP (*N* = 8/group) (F) and vascular remodeling (α-smooth muscle actin stain, α-SMA), as evidenced by increased percent of muscularized (< 100 μm) pulmonary vessels (G) and increased medial thickening relative to vessel diameter when compared with miR-Control (H) (*N* = 6/group). **P = 0.0021 for (F), **P = 0.0072 for (G), *P = 0.0335 for (H).

Data information: Error bars reflect SEM. Mouse tissue scale bar: 50 μm.

**Figure 5:**
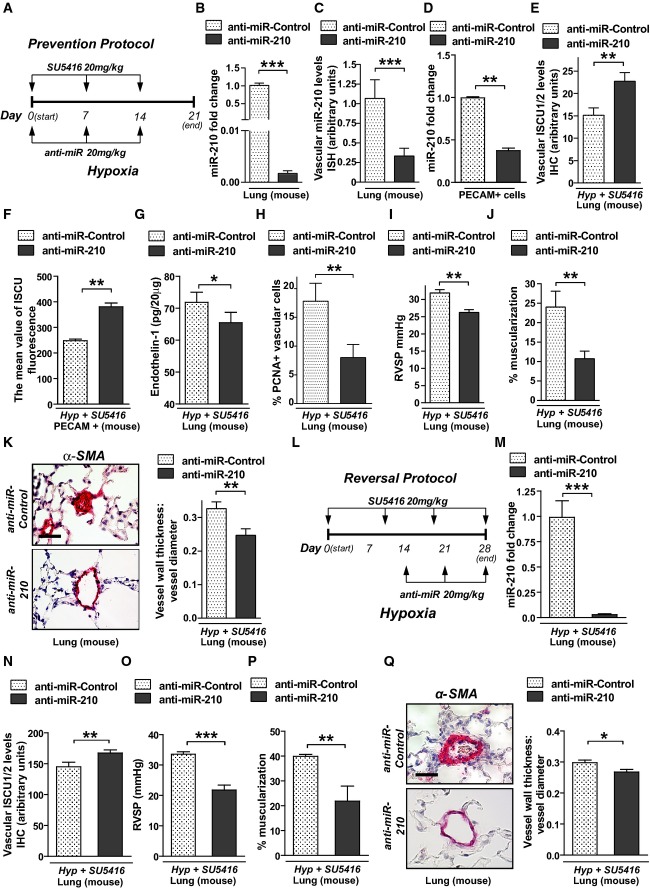
Antisense inhibition of miR-210 protects against and improves existing manifestations of PH *in vivo*

Separately, endogenous miR-210 in WT mice was pharmacologically inhibited by serial weekly intravenous injections of 2′-F- and 2′-O-methoxyethyl (2′MOE)-modified anti-miR-210 oligonucleotides, as we previously described (Bertero *et al*, 2014). A regimen to prevent PH development (“prevention study”; Fig5A) down-regulated miR-210 expression in lungs of mice exposed to hypoxia + SU5416, as assessed by RT–qPCR of whole lung (Fig5B), by RT–qPCR of PECAM^+^ pulmonary vascular cells from diseased lungs (Fig5C), and by *in situ* pulmonary vascular staining (Fig5D). This decrease of miR-210 was accompanied by de-repression of ISCU1/2 expression as assessed by *in situ* staining (Fig5E) and flow cytometric assessment of PECAM^+^ endothelial cells (Fig5F). Anti-miR-210 prevented endothelin-1 up-regulation (Supplementary Fig S12B), and, as in *miR-210*^−/−^ mice, anti-miR-210 also reduced the number of proliferating PCNA-positive vascular cells (Fig5H). As a result, anti-miR-210 prevented RVSP elevation (Fig5I) and vessel remodeling (Fig5J and K) as compared with mice exposed to anti-miR-Control. Similarly, when administered to ameliorate already existing PH [using a “reversal study” protocol which we previously described (Bertero *et al*, 2014)] (Fig5L), anti-miR-210 similarly decreased pulmonary vascular miR-210 (Fig5M), increased ISCU1/2 expression (Fig5N), and significantly ameliorated elevations of RVSP and vessel remodeling (Fig5O–Q).

A Schema of the strategy to determine whether antisense inhibition of miR-210 (anti-miR-210) prevents PH secondary to Hyp + SU5416 exposure (“prevention protocol”).

B–D Intravenous delivery of anti-miR-210 down-regulated miR-210 expression in whole lung tissue (*N* = 7/group), ***P < 0.0001 (B), the pulmonary vasculature as assessed by *in situ* staining (*N* = 7/group), ***P = 0.0003 (C), and PECAM^+^ vascular endothelial cells derived from whole lung tissue (*N* = 4/group), **P = 0.0013 (D).

E Correspondingly, anti-miR-210 delivery resulted in preservation of ISCU1/2 expression in < 100-μm pulmonary vessels as compared to anti-miR-Control (*N* = 5/group), **P = 0.0011.

F Similarly, ISCU1/2 expression was preserved in PECAM^+^ endothelial cells derived from mice treated with anti-miR-210 (*N* = 4/group), **P = 0.0016.

G Endothelin-1 was decreased in lung tissue from mice treated with anti-miR-210 during Hyp + SU5416 exposure (*N* = 6/group), *P = 0.0181.

H As assessed by *in situ* immunofluorescence, PCNA was decreased in pulmonary vessels (< 100 μm) after anti-miR-210 delivery, **P = 0.001.

I–K Anti-miR-210 delivery (*N* = 10/group) ameliorated the elevation of RVSP, **P = 0.0031 (i) and vascular remodeling, as reflected by increased percent of muscularized arterioles (*N* = 8/group, **P = 0.0091) (J) and increased medial thickness relative to vessel diameter in < 100-μm pulmonary vessels (*N* = 6/group, **P = 0.0021) (K).

L Schema of the strategy to determine whether anti-miR-210 improves existing PH (“reversal protocol”).

M, N Pharmacologic inhibition of miR-210 (*N* = 7/group) down-regulated pulmonary expression of miR-210, ***P < 0.0001 (M), and preserved ISCU1/2 expression in < 100-μm pulmonary vessels, **P = 0.0015 (N).

O–Q In contrast to anti-miR-Control (*N* = 8), anti-miR-210 ameliorated the elevation of RVSP (*N* = 10/group, ***P < 0.0001) (O) and pulmonary vascular remodeling (*N* = 7/group, **P = 0.0022, *P = 0.0167) (P–Q).

Data information: Error bars reflect SEM. Mouse tissue scale bar: 50 μm.

**Figure 6:**
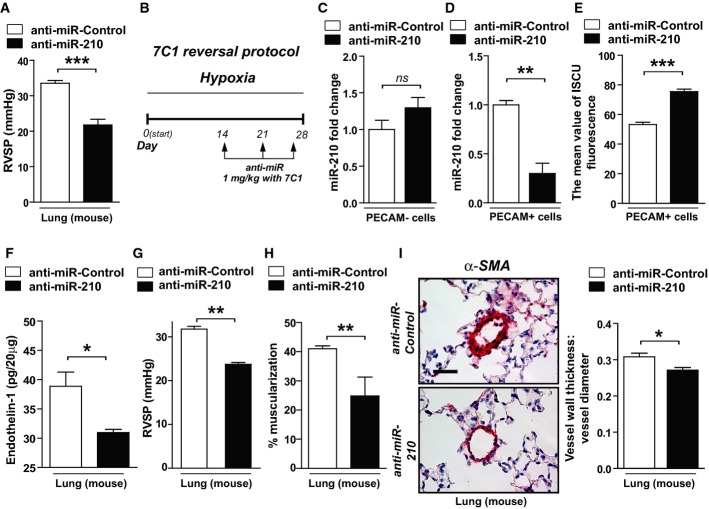
Antisense inhibition of miR-210 specifically in vascular endothelium improves existing manifestations of PH *in vivo*

To ensure that SU5416 was not confounding the actions of anti-miR-210, mice were exposed to anti-miR-210 and hypoxia alone with significant prevention of hemodynamic manifestations of PH (Fig6A). In that context, to determine whether endothelial-specific actions of the miR-210-ISCU1/2 axis are necessary for hypoxia-induced PH development, serial delivery *in vivo* of anti-miR-210 to the pulmonary vascular endothelium was achieved using the recently described 7C1 nanoparticle delivery system (Dahlman *et al*, 2014). When intravenously administered to ameliorate already existing PH induced by hypoxia alone without SU5416 (“reversal study”; Fig6B), endothelial delivery of 7C1-encapsulated anti-miR-210 led to a decrease in miR-210 specifically in PECAM^+^ pulmonary vascular endothelial cells but not in other PECAM-negative pulmonary cells (Fig6C). Corresponding with such endothelial-specific delivery, miR-210 level was decreased and ISCU1/2 expression was increased in those same PECAM^+^ cells (Fig6D and E). Mirroring the effects of miR-210 in cultured PAECs (Supplementary Fig S12B) and the effects of systemic delivery of anti-miR-210 (Fig5G), endothelin-1 was decreased by anti-miR-210 as compared to control (Fig6F). Moreover, such endothelial-specific miR-210 repression significantly ameliorated elevations of RVSP (Fig6G) and vessel remodeling (Fig6H and I). Thus, using both genetic and pharmacologic methods, we conclude that chronic induction of endogenous miR-210, particularly in endothelial cells, is necessary and sufficient to promote hypoxia-induced PH *in vivo*.

A Following the schema of Fig5A in the presence of hypoxia but the absence of SU5416, intravenous delivery of anti-miR-210 ameliorated the elevation of RVSP compared with anti-miR-Control (*N* = 6/group, ***P = 0.001).

B Schema of the strategy to determine whether delivery of anti-miR-210 specifically to the vascular endothelium via packaging with 7C1 nanoparticles improves hypoxia-induced PH (“7C1 reversal protocol”).

C, D Consistent with specific delivery to the vascular endothelium after intravenous administration (Dahlman *et al*, 2014), 7C1-mediated delivery of anti-miR-210 (*N* = 6/group) decreased miR-210 expression in PECAM^+^ pulmonary vascular endothelial cells, **P = 0.0013, (D) but not PECAM-negative pulmonary cells, NS *P* = 0.3775 (C).

E Correspondingly, endothelial-specific anti-miR-210 (*N* = 6/group) increased ISCU1/2 expression in PECAM^+^ cells even in the presence of chronic hypoxia, ***P = 0.0003.

F–I Delivery of anti-miR-210 (*N* = 5/group) decreased endothelin-1 in hypoxic mouse lung tissue, *P = 0.0198 (F). As a result, in contrast to anti-miR-Control (*N* = 8), endothelial-specific anti-miR-210 ameliorated the elevation of RVSP (*N* = 6), **P = 0.0024 (G), and pulmonary vascular remodeling (*N* = 6/group), **P = 0.0021, *P = 0.0117 (H–I).

Data information: Error bars reflect SEM. Mouse tissue scale bar: 50 μm.

### Knockdown of ISCU1/2 independent of miR-210 in the pulmonary vasculature promotes PH

**Figure 7:**
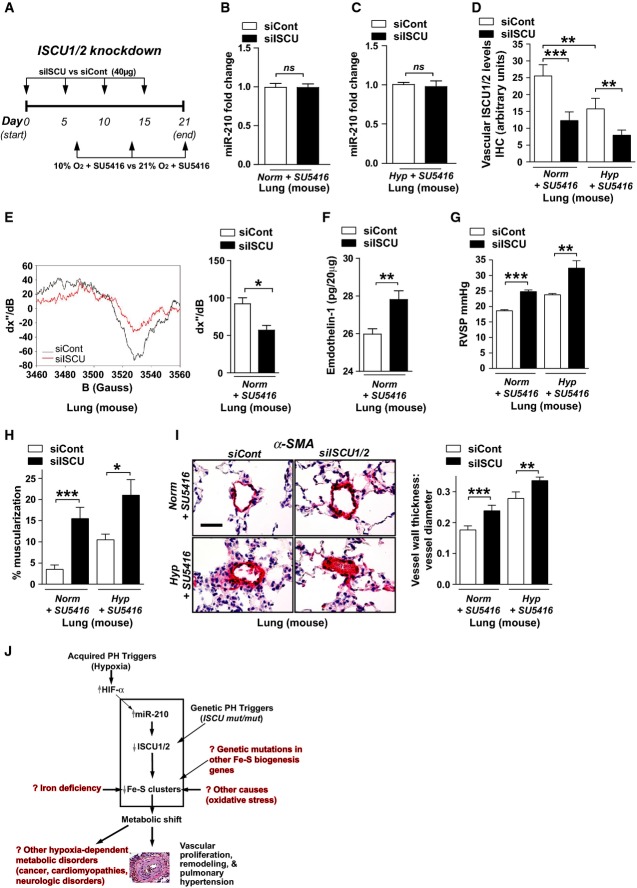
siRNA knockdown of pulmonary vascular ISCU1/2 *in vivo* promotes PH

To demonstrate definitively the importance of ISCU1/2 and Fe-S biogenesis in the actions of miR-210 and hypoxia in PH, we assessed the consequences of repressing ISCU1/2 in the pulmonary vasculature independent of hypoxia or miR-210 manipulation. To do so, we utilized the Staramine-mPEG nanocomplex intravenous system to intravenously deliver siRNAs directly to the pulmonary vasculature of mice *in vivo* (Polach *et al*, 2012) (Fig7A). First, delivery of siISCU did not alter miR-210 expression either under normoxia or hypoxia compared to the siControl mice (Fig7B and C). Compared to control siRNA-treated littermate mice (siCont), mice that were administered a siRNA targeting murine ISCU1/2 (siISCU, efficiency of knockdown in cultured MEFs as shown in Supplementary Fig S8B) exhibited a reduction of ISCU1/2 in the pulmonary vasculature (Fig7D), a consequent decrease in EPR-measured Fe-S integrity (Fig7E) without a reduction in total mitochondrial DNA content (Supplementary Fig S9D), and an up-regulation of endothelin-1 expression (Fig7F). As a result, in the presence of either normoxia + SU5416 or hypoxia + SU5416 (days 7–21), ISCU1/2 knockdown resulted in RVSP elevation (Fig7G) as well as increased vessel remodeling (Fig7H and I) as compared with siCont-treated littermate controls. Again, left ventricular dysfunction was exonerated as a cause for PH (Supplementary Fig S13B). Thus, similar to both hypoxia and forced miR-210 expression, knockdown of ISCU1/2 promoted PH, thereby confirming the direct importance of Fe-S biogenesis in pulmonary vascular homeostasis and PH manifestation *in vivo*.

A Schema of the strategy for pharmacologic inhibition of siRNA knockdown of ISCU1/2 in the pulmonary vasculature of mice *in vivo* by Staramine-mPEG-mediated intravenous delivery.

B, C Pharmacologic inhibition of ISCU (*N* = 8/group) did not influence miR-210 expression in the lungs either after Norm + SU5415, NS *P* = 0.8634 (B) or Hyp + SU5416, NS *P* = 0.9786 (C).

D As compared with control siRNA (siCont) where ISCU1/2 expression was decreased after exposure to Hyp + SU5416 (white bars), pulmonary vascular delivery of siRNA specific for ISCU1/2 (siISCU) via Staramine-mPEG nanocomplexes (Polach *et al*, 2012) down-regulated ISCU1/2 in < 100-μm pulmonary vessels in Norm + SU5416 and Hyp + SU5416 (*N* = 5/group, ***P = 0.0006, **P = 0.0019 for Hyp + SU5416, **P = 0.0029 for siCont).

E By electron paramagnetic resonance (EPR) spectroscopy (representative siCont versus siISCU lung), Fe-S cluster signal was decreased (quantitative graph, right) in siISCU mouse lung tissue (*N* = 4 mice) compared with siCont (*N* = 5 mice), *P = 0.0143.

F Endothelin-1 was increased in lung tissue after siISCU delivery under normoxic conditions (*N* = 5/group), **P = 0.0027.

G–I In the absence (left bars) and presence (right bars) of Hyp + SU5416 for 2 weeks, siISCU (black bars) induced elevations in RVSP (***P < 0.0001, **P = 0.0031) (G) and increased pulmonary vascular remodeling in < 100-μm pulmonary vessels (H, I) as compared with siCont (*N* = 5/group, ***P = 0.0003, *P = 0.0114 for H; ***P = 0.0006, **P = 0.0098 for I).

J Model of acquired and genetic Fe-S deficiency as a central cause of metabolic dysfunction and PH in mice and humans. Among others, potential translational implications are highlighted in red text.

Data information: Error bars reflect SEM. Mouse tissue scale bar: 50 μm.

### Exercise-induced pulmonary vascular dysfunction in an individual genetically deficient for ISCU

Beyond hypoxic activation of miR-210, other triggers, independent of hypoxia and HIF, may reduce ISCU1/2 and thus provoke pulmonary vascular dysfunction. Exceptionally rare homozygous intronic *ISCU* mutations in humans exist, causing defective *ISCU* transcript splicing and accompanied by profound, but not complete, deficiency in ISCU activity (Rouault & Tong, 2008). Such persons are plagued by a mitochondrial myopathy and lactic acidosis. Importantly, such persons also suffer from extreme exertional dyspnea which has never been fully explained by myopathy alone (Linderholm *et al*, 1969; Heinicke *et al*, 2011) but is consistent with exercise-induced pulmonary vascular dysfunction (so-called exercise-induced PAH') (Tolle *et al*, 2008b). Guided by our cellular and rodent findings above, we sought to determine whether persons genetically deficient in *ISCU* exhibit manifestations of PH either at rest or with exercise.

A 29-year-old woman with known homozygous intronic *ISCU* mutations was recruited for study (Sanaker *et al*, 2010). At rest, echocardiography revealed normal right ventricular function and size, while pulmonary arterial catheterization revealed normal right and left heart filling pressure but with a mean pulmonary arterial pressure at the upper limits of normal [mPAP = 21 mmHg; normal < 20.6 mmHg (Badesch *et al*, 2009)]. Advanced cardiopulmonary exercise testing revealed exertional dyspnea with a markedly depressed VO_2max_ (495 ml/min, 25% of predicted) that was driven largely by impaired systemic oxygen extraction [Ca-vO_2max_ = 4.4 m/dl (33% of predicted)], corresponding with her known mitochondrial myopathy. There was no pulmonary mechanical limit (VE_max_/MVV = 28%), but there was mixed lactic acidemia (2.7–7.2 mM, rest to peak exercise) and respiratory alkalemia at peak exercise (PaCO_2_ = 28 mmHg, arterial pH 7.38)—the latter well described in patients with mitochondrial myopathies (Heinicke *et al*, 2011). Importantly, abnormally increased pulmonary vascular resistance (PVR) during exercise (maximal PVR at exercise = 135 dynes s/cm^5^, normal < 80 dynes s/cm^5^ in persons < 50 years (Groves *et al*, 1987; Tolle *et al*, 2008b)] was observed, accompanied by elevated mean PAP [maximal mPAP = 31 mmHg; normal < 29 mmHg in persons < 50 years (Badesch *et al*, 2009)], together consistent with exercise-induced pulmonary vascular dysfunction as defined by contemporary diagnostic criteria (Tolle *et al*, 2008b). A pulmonary vasculopathy was also endorsed by increased ΔPAP/ΔCO and ΔTPG/ΔPCWP ratios (Table1) (Lewis, 2010), and an elevated VE/VCO_2_ slope (50.5; normal < 30) was consistent with a pulmonary vasculopathy (Ting *et al*, 2001) coupled with her mitochondrial myopathy and hyperventilation. Finally, a 4-week treatment with an oral pulmonary vasodilator [phosphodiesterase 5 (PDE5) inhibitor tadalafil] significantly increased her functional capacity, as quantified by an improvement of > 100 m on 6-min walk test (6 MWT, Table2). Together, these data comprise the first-known observation of clinically significant pulmonary vascular disease in individuals genetically deficient in ISCU1/2, thus carrying implications for clinical care paradigms in these persons. Moreover, together with our cellular and rodent studies, these results confirm the direct causative relationship of the miR-210-ISCU axis with PH, as driven by either acquired (hypoxia driven) or human genetic (*ISCU mut/mut*) alterations.

**Table 1:** Exercise-induced pulmonary vascular dysfunction in an *ISCU mut/mut* individual

Hemodynamic index	Rest	Peak exercise
RAP (mmHg)	5	12
Mean PAP (mmHg)	21	31
PCWP (mmHg)	7	10
CO (l/min)	6.5	11
PVR (dynes s/cm^5^)	160	135 (normal < 80 for persons < 50 years)
ΔPAP/ΔCO (mmHg/l/min)		2.2 (normal < 2 for persons < 50 years)
ΔTPG/ΔPCWP		2.3 (normal < 1)

Advanced cardiopulmonary exercise testing was performed on a woman with homozygous *ISCU* mutations. At rest, she displayed normal right atrial pressure (RAP) and pulmonary capillary wedge pressure (PCWP) but a mean pulmonary arterial pressure (PAP) at the upper limit of normal. During exercise, RAP and mean PAP were elevated but with PCWP remaining normal. Most importantly, abnormal indices of pulmonary vascular resistance (PVR) were evident (including lack of an appropriate decrease in PVR during exercise and increased ratios of ΔPAP/ΔCO and ΔTPG/ΔPCWP**)**, thus fulfilling criteria for “exercise-induced PAH” that do not rely solely upon changes in mean PAP alone (Tolle *et al*, 2008b; Lewis, 2010).

**Table 2:** Improvement of 6-min walk test (6 MWT) in an *ISCU mut/mut* individual after initiation of pulmonary vasodilator therapy (PDE5 inhibitor)

Pulmonary vasodilator	6MW distance (meters)
None	240
Tadalafil (20 mg daily for 7 days followed by 40 mg daily for 40 days)	360
Tadalafil (20 mg daily for 7 days followed by 40 mg daily for 166 days)	364

## Discussion

We present evidence in mice and humans whereby the miR-210-ISCU1/2 axis regulates the pulmonary vascular and endothelial expression of Fe-S clusters both through acquired injury (e.g. hypoxia) and genetic mutations. As the dynamic functions of Fe-S clusters in most disease states have not been investigated due to substantial technical challenges, these findings to our knowledge constitute the first targeted analysis of these prosthetic groups in vascular tissue, pulmonary or otherwise. Furthermore, using genetic and pharmacologic methods to perform gain-of-function and loss-of-function analyses in the rodent pulmonary vasculature *in vivo* coupled with the first-known observation of pulmonary vasculopathy in an *ISCU mut/mut* human, we define the miR-210-ISCU1/2 axis as an overarching upstream pathogenic mechanism, central to the vascular and endothelial metabolic alterations that promote PH (Fig7J).

These findings emphasize both the importance and novelty of ISCU1/2 and its regulation of Fe-S cluster integrity critical to pulmonary vascular pathogenesis. Mechanistic plausibility is supported by case reports linking defects in mitochondrial oxidative phosphorylation to PH (Barclay *et al*, 2005) as well as human genetic evidence associating mutations in the Fe-S biogenesis protein NFU1 with pulmonary vascular remodeling (Navarro-Sastre *et al*, 2011). While other factors have been identified that modulate metabolic alterations in PH (as reviewed by Tuder *et al*, 2011; Cottrill & Chan, 2013), many of these are canonical protein factors operating through previously well-explored processes—such as altered tricarboxylic acid cycle enzyme activity, mitochondrial membrane potential, or coupling of oxidative phosphorylation to energy production. In contrast, alteration of mitochondrial respiratory complexes has been reported primarily after genetic mutation [such as in cancer (Brandon *et al*, 2006)] but rarely via a dynamic regulatory pathway in acquired mammalian diseases. Our findings offer an alternative paradigm of pathogenesis whereby mitochondrial respiratory activity is dysregulated, independent of respiratory complex protein expression but critically dependent on miR-210, ISCU1/2, and Fe-S integrity. Because Fe-S clusters are not represented by the transcriptome or proteome, it is not surprising that this pathogenic axis has been missed until now. Therefore, our results not only provide a mechanistic explanation linking Fe-S biogenesis and mitochondrial respiratory activity to PH but also establish PH as the first of perhaps many mammalian “iron–sulfur” diseases driven by miR-210, ISCU1/2, and deficiencies in Fe-S clusters.

Beyond mitochondrial metabolism, it is also possible that other Fe-S-dependent mechanisms may contribute to consequent PH manifestations. For example, Fe-S clusters have been implicated in chromosomal and telomere homeostasis (Netz *et al*, 2012; Stehling *et al*, 2012), consistent with the association of telomere length and vascular senescence in PH (Noureddine *et al*, 2011). Alternatively, in cases of genetic deficiencies of Fe-S biogenesis (Rouault, 2012) and including deficiencies of ISCU1/2 (Haller *et al*, 1991; Mochel *et al*, 2008), decreases in Fe-S clusters alter cellular iron homeostasis and increase mitochondrial iron deposition. Consistent with previous descriptions of alternative gene targets of miR-210 important in iron transport (Yoshioka *et al*, 2012; Qiao *et al*, 2013), it is possible that miR-210 may exert additional control over iron homeostasis *in vivo*. For example, while iron content in whole-cell lysates of miR-210-expressing PAECs was unchanged compared with control PAECs (consistent with the findings of unchanged total pulmonary iron content in hypoxic mice, Supplementary Fig S10F), mitochondrial iron levels were greater with miR-210 expression as compared with control (Supplementary Fig S15). Therefore, given that direct alterations in iron handling and transport have been identified as a cause for PH in mice (Ghosh *et al*, 2013), the complex actions of the miR-210-ISCU1/2 axis in iron transport may be relevant for PH pathogenesis even beyond their effects on Fe-S biogenesis and warrant future investigation.

Upstream of ISCU1/2, our work highlights miR-210 as one of the several miRNAs known to modulate pulmonary vascular function and PH *in vivo*. Similar to the previously reported context-specific target gene predilection for miR-21 in PH (Parikh *et al*, 2012), the apparent favoring of ISCU1/2 as a target of miR-210 in the hypoxic pulmonary endothelium is not an uncommon scenario in miRNA biology where, with increased specific miRNA expression, only a subset of its validated gene targets may display a net down-regulation. Specific stoichiometry of miRNA to target transcript levels may dictate the overall effectiveness of target gene down-regulation (Parikh *et al*, 2012). Alternatively, indirect regulatory pathways may exist in hypoxia that ultimately may be more powerful stimuli on certain target genes than any direct miR-210 engagement of the target transcripts themselves. Such complex regulation may serve as homeostatic rheostats. Both possibilities may be active in this case, resulting in an apparent context-specific predilection for ISCU1/2 to be subject to net down-regulation. Nonetheless, other gene targets of miR-210 may still influence PH via processes beyond mitochondrial function and in cell types other than the pulmonary endothelium (Chan & Loscalzo, 2010; Gou *et al*, 2012). Yet, considering our results demonstrating PH manifestation after repressing ISCU1/2 alone (Fig7), we conclude that the actions of miR-210 in the pulmonary vasculature in large part are concentrated on Fe-S biogenesis and downstream mitochondrial dysfunction. Comparison of the roles of miR-210 in different cellular and disease contexts may further highlight these differences. Additionally, given the number of potential Fe-S biogenesis targets (Rouault & Tong, 2008; Rouault, 2012) beyond ISCU1/2, it is plausible that unidentified miRNAs act in concert with miR-210 to control further Fe-S biogenesis and other PH manifestations.

Our findings also emphasize the direct relevance of the miR-210-ISCU1/2-Fe-S regulatory axis and mitochondrial metabolic dysfunction in pulmonary arterial endothelial cells for overall PH development. Previous studies have characterized the metabolic dysfunction in both PASMCs and cardiomyocytes in the right ventricle in PH but have rarely focused on metabolic dysfunction in endothelial cells (as reviewed in Cottrill & Chan, 2013). Although mitochondrial oxidative phosphorylation accounts for a minority of endothelial energy production at baseline, further down-regulation of such metabolic activity in favor of glycolysis nonetheless has been observed in diseased PAECs from humans with idiopathic PAH (Xu *et al*, 2007), from lambs with PAH secondary to increased pulmonary blood flow (Sun *et al*, 2013), and from monocrotaline-treated rats with PAH (Sun *et al*, 2014). Yet, this is the first report to our knowledge that implicates this metabolic dysfunction of endothelial cells as a causative pathogenic event essential to increased cellular proliferation (Fig3H), release of endothelin-1 (Figs6), and overall PH manifestation *in vivo* (Fig6). Such results support a number of existing models of PH where mitochondrial dysregulation can trigger a pro-proliferative phenotype via modulation of oxidative stress, cellular growth factors, and cellular survival (Cottrill & Chan, 2013). Indeed, independent of Fe-S biogenesis, a number of clinical syndromes related to altered mitochondrial respiration manifest with PH (Sproule *et al*, 2008; Hung *et al*, 2012; Rivera *et al*, 2013; Catteruccia *et al*, 2014), thus supporting the direct clinical connection of mitochondrial dysfunction with this disease. Beyond proliferation, other end-stage phenotypes of PH may also be directly regulated by mitochondrial function (e.g. vasomotor tone, thrombosis, vascular stiffening, among others), and future mechanistic studies linking such metabolic dysregulation to such pathophenotypes will be crucial for development of improved metabolic therapies in PH. So, while the miR-210-ISCU1/2 axis indeed appears active at least to a certain degree in diseased PASMCs (Fig1C and F, Supplementary Fig S16), our results demonstrate the particularly robust actions of miR-210 in PAECs (Chan *et al*, 2009). Thus, the elucidation of the miR-210-ISCU1/2-Fe-S axis expands the pervasive relevance of mitochondrial dysfunction in additional vascular cell types beyond muscle tissue in PH and emphasizes the therapeutic potential of targeting this metabolic pathway in vascular endothelium.

Stemming from the paucity of human tissue samples derived during PH development and intrinsic differences of PH among rodents and humans, more direct evidence of causal pathogenic mechanisms in pulmonary vascular disease in humans has been especially difficult to generate. In this case, advanced cardiopulmonary exercise testing successfully unmasked a pathophenotype of pulmonary vascular dysfunction in a rare *ISCU mut/mut* individual, using contemporary criteria of “exercise-induced PAH” and consistent with prior descriptions in other contexts (Kovacs *et al*, 2009; Fowler *et al*, 2011)—with exercise, a blunted fall in PVR to values > 80 dynes s/cm^5^ along with mPAP > 30 mmHg in persons < 50 years (Groves *et al*, 1987; Tolle *et al*, 2008b). Notably, the hemodynamic dysfunction was modest. Nonetheless, this person's relatively young age coupled with our ability to make precise invasive hemodynamic measurements allowed us to avoid the pitfalls of “normal” variations of mPAP and PVR during exercise (Naeije *et al*, 2013) reported previously by echocardiography (Argiento *et al*, 2012) and in the elderly (Kovacs *et al*, 2009, 2012). Thus, the presence of a primary pulmonary vasculopathy was unambiguous by these criteria (Tolle *et al*, 2008b) and was further corroborated by abnormalities in the pressure–flow relation with exercise, the transpulmonary pressure gradient, and the VE/VCO_2_ slope in relation to her age. This finding of pulmonary vascular dysfunction was especially timely—adding to our rapidly evolving understanding of exercise-induced pulmonary vascular pathology and in guiding new clinical care paradigms for this patient population. Specifically, the patient's functional improvement on pulmonary vasodilator therapy with a PDE5 inhibitor suggested a clinical benefit of treating such an exercise-specific pathophenotype. Notably, as exercise-induced PAH may increase the risk of more severe PAH in the future (Tolle *et al*, 2008b), it remains to be seen whether such early clinical treatment in these individuals could retard the overall development of disease in these individuals. Finally, in light of advancing evidence of the causative interplay between impaired limb skeletal muscle oxidative metabolism and PAH in other populations (Tolle *et al*, 2008a), it is an intriguing possibility that mitochondrial dysfunction in both limb muscle and pulmonary vasculature in this individual may be responsible for her pulmonary vascular pathophenotype.

In that vein, the concept of miR-210, ISCU1/2, and Fe-S deficiency as pathogenic determinants in PH has even further translational significance for improving both diagnosis and therapeutic management of this disease. The finding of increased levels of plasma-based miR-210 in a small cohort of PH patients (Fig1E) could serve as the basis for the future study of miR-210 as a specific biomarker for PH. Moreover, we have recently shown that extracellular miR-210 can be taken up by recipient tissue to induce ISCU1/2 down-regulation and metabolic effects beyond the source tissue (Hale *et al*, 2014). Thus, diseased pulmonary vasculature may even rely upon circulating miR-210 as a molecular messenger to communicate with other recipient tissues during disease progression. Moreover, our newly found appreciation for the direct pathogenic importance of Fe-S deficiency could aid our ability to identify additional populations suffering from PH or at risk for PH who may benefit from PH-specific medications. For example, iron deficiency may affect Fe-S clusters via simple substrate deprivation, thus offering an explanation for the puzzling clinical association of PH and iron deficiency (Rhodes *et al*, 2011; Ruiter *et al*, 2011; Soon *et al*, 2011). Alternatively, rare genetic deficiencies in genes other than *ISCU* yet critically important in Fe-S biogenesis (e.g. frataxin, ISD11) may also predispose to PH, perhaps exacerbated in the setting of hypoxia. Thus, attempts to identify such Fe-S-specific conditions that predispose to PH development—conditions that otherwise have been ignored to date—could be especially fruitful.

Our findings also have broad implications on our understanding of the metabolic origins of PH and may direct us toward more effective therapeutic targets. The distinct HIF and hypoxia dependence of miR-210 coupled with the hypoxia-independent genetic (*ISCU mut/mut*) predisposition to PH demonstrates the wide spectrum of human PH subtypes where control of Fe-S integrity controls pathogenesis, encompassing at least Group 1 PAH (HIF dependent), Group 3 PH (hypoxia dependent), and likely Group 5 PH (“metabolic” dysfunction) (Simonneau *et al*, 2013). Future determination of other PH subtypes that may depend upon Fe-S deficiency could also aid in tailoring specific therapies in targeted subtypes based on Fe-S-dependent metabolic disease origin. To date, no small molecules have been developed that directly control the expression of Fe-S clusters, but further optimization of a quantitative system for measurement of Fe-S levels (Fig2) may hold the key for high-throughput drug screening. Pharmacologic inhibition of miR-210 (Figs5 and 6) also could be promising in humans, especially with the advent of specific anti-miR delivery methods to the pulmonary vasculature and endothelium (Figs6 and 7). Finally, this work could serve as a foundation for discovery of the actions of miR-210, ISCU1/2, and Fe-S biology in additional diseases beyond PH that may share similar hypoxic, genetic, and metabolic underpinnings.

## Materials and Methods

### Study design

The study objective was to determine the molecular mechanism by which Fe-S clusters are repressed in PH and whether such alterations in Fe-S clusters directly cause metabolic dysregulation and PH. First, using a fluorescent GRX2 transgene sensor system, Fe-S clusters were quantified in diseased PECAM^+^ endothelial cells from PH mice and in cultured cells in hypoxia or after manipulation of either miR-210 or ISCU1/2. Fe-S clusters were independently measured by electron paramagnetic resonance (EPR) spectroscopy of human lung tissue collected at lung transplantation from non-diseased or PH patients. The number of patient samples for EPR was limited by clinical availability and was calculated to measure a 30% difference between the means of experimental and control groups with a power of 80% and *P*-value of 0.01. Diseased pulmonary tissue from mice and separate cohorts of human patients was subjected to quantitative analysis by RT–PCR and *in situ* histology to measure expression of miR-210 and ISCU1/2. The number of patient samples for histologic analysis was calculated to measure a 20% difference between the means of experimental and control groups with a power of 80% and *P*-value of 0.01. Gain-of-function and loss-of-function experiments were also performed to manipulate miR-210 and ISCU1/2 expression in the pulmonary vasculature of mice, followed by molecular and physiologic analyses relevant to Fe-S-dependent functions. The number of animals in each group was calculated to measure a 25% difference between the means of experimental and control groups with a power of 80% and *P*-value of 0.01. Animal treatments were conducted in a controlled and non-blinded manner. Finally, a rare *ISCU mut/mut* individual was recruited for advanced cardiopulmonary exercise testing in order to determine undiagnosed pulmonary vascular disease. EPR experiments on human tissue, *in situ* expression/histologic analyses of both mouse and human tissue, and respiratory complex activity and pulmonary vascular hemodynamics in mice were performed in a blinded fashion.

### Ethical approval and human study participants

All experimental procedures involving the use of human tissue and plasma were approved by the Partners Healthcare, Boston Children's Hospital, and University of California, Los Angeles Institutional Review Boards, and the New England Organ Bank. Ethical approval for this study conformed to the standards of the Declaration of Helsinki and the Department of Health and Human Services Belmont Report. Informed consent from the *ISCU mut/mut* individual was obtained for advanced cardiopulmonary exercise testing. For formalin-fixed paraffin-embedded lung samples, human PH specimens were collected from unused or discarded surgical samples; non-diseased human lung specimens from the New England Organ Bank have been described (Kajstura *et al*, 2011). For fresh-frozen lung tissue, human PH specimens were collected from patients undergoing lung transplantation; non-PH specimens were collected from lungs that were deemed unsuitable as donor organs for unrelated reasons. For plasma harvest and analysis, human subjects were chosen with clinically significant dyspnea and undergoing right heart catheterization. Subjects were stratified by the presence or absence of clinical PH, as defined by elevated mean pulmonary arterial pressure > 25 mmHg (mean PAP).

### Mouse models

The Harvard Center for Comparative Medicine approved the use of animals in these experiments; such housing, husbandry, and use adhered to the NIH Guide for the Care and Use of Laboratory Animals. Their descriptions are detailed in Supplementary Materials and Methods.

### Advanced cardiopulmonary exercise testing

An individual with known homozygous intronic mutations in *ISCU* (*ISCU mut/mut*) (Sanaker *et al*, 2010) was referred for a clinically indicated advanced cardiopulmonary exercise test (CPET), stemming from severe exercise intolerance and dyspnea. Advanced CPET was performed as described in detail (Tolle *et al*, 2008b) and in Supplementary Materials and Methods.

### Statistics

Cell culture experiments were performed at least three times and at least in triplicate for each replicate. The number of animals in each group was calculated to measure at least a 20% difference between the means of experimental and control groups with a power of 80% and standard deviation of 10%. Randomization of animals was not necessary for adequate analysis. The number of unique patient samples for this study was determined primarily by clinical availability. *In situ* expression/histologic analyses of both rodent and human tissue and pulmonary vascular hemodynamics in mice and rats were performed in a blinded fashion. Immunoblot images are representative of experiments that have been repeated at least three times. Micrographs are representative of experiments in each relevant cohort. Numerical quantifications represent mean ± standard error of the mean for three or more independent experiments. Images are representative of experiments that were repeated at least three times. In appropriately powered datasets, statistical tests were selected based on the number of experimental groups and nature of its variables. Frequency distribution histogram analysis was assessed for normal distribution. Range and standard deviation were used as indices of variation within each data group and were used for comparison of variance across statistically comparable groups. Paired samples were compared by a two-tailed Student's *t-*test. Comparison of multiple samples was performed by ANOVA followed by Student–Newman–Keuls *post hoc* testing. A *P* < 0.05 was considered significant.

### Additional information

See Supplementary Materials and Methods regarding plasmids, mouse models, and protocols of cell culture, transfection, immunoblotting, lentivirus production, ELISA, RNA extraction and analysis, echocardiography, tissue harvest and *in situ* staining, respiratory Complex I activity assays, EPR spectroscopy, PECAM^+^ cell separation and flow cytometry, delivery of miRNA mimic oligonucleotides, miRNA inhibitors, and siRNAs to the pulmonary vasculature of mice, and advanced CPET, as we have previously described (Chan *et al*, 2009; Parikh *et al*, 2012; Bertero *et al*, 2014).

## For more information

The Chan lab website: http://www.sychanlab.net/

## Supporting Information

Source Data for Supplementary Figure S15

Review Process File
