# Supplementary figures and images for: Genetic and hypoxic alterations of the microRNA-210-ISCU1/2 axis promote iron–sulfur deficiency and pulmonary hypertension
Source: EMBO Mol Med. 2015 Mar 30;7(6):695–713. doi: 10.15252/emmm.201404511 (PMC4459813; doi:10.15252/emmm.201404511)

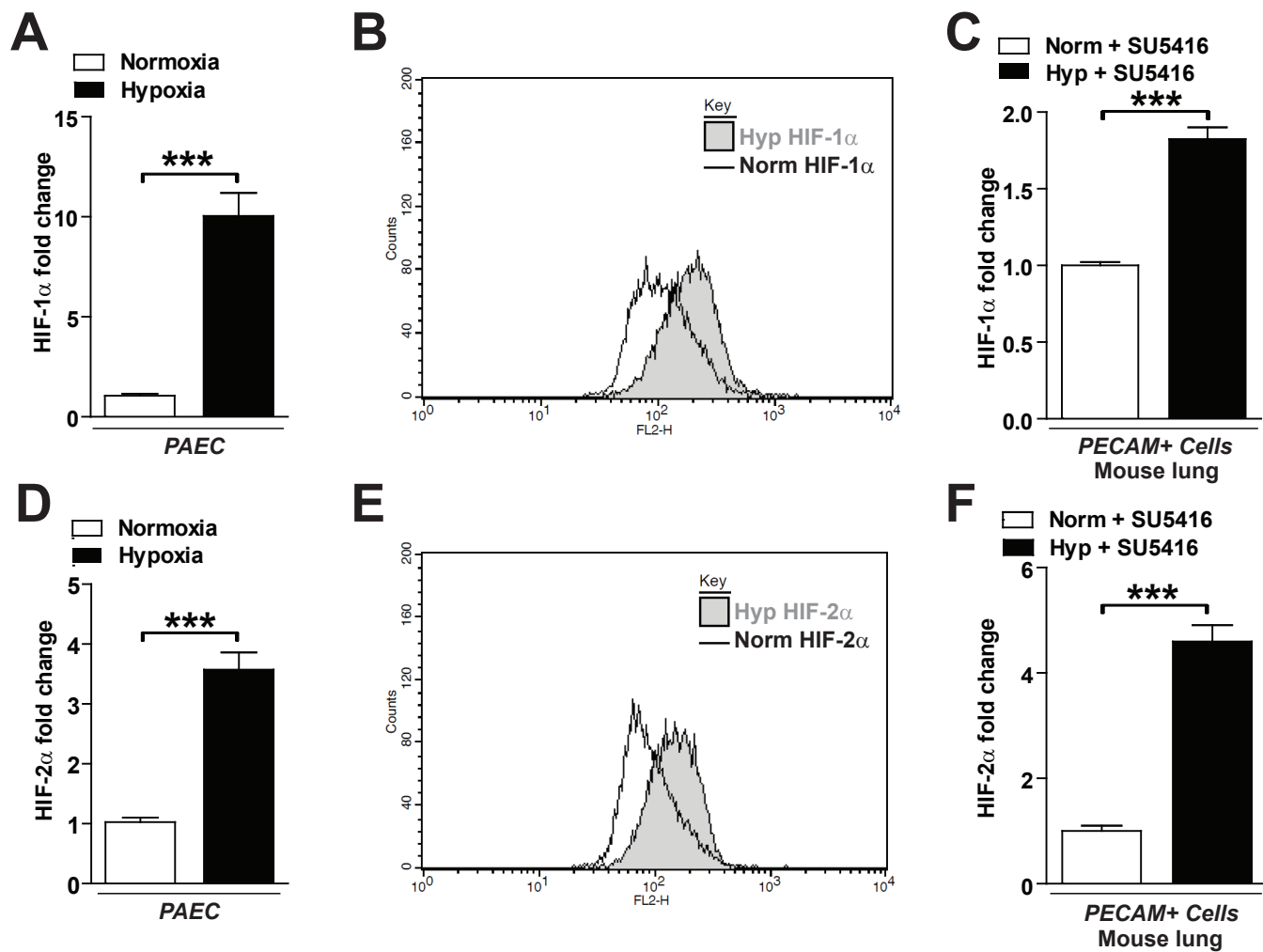

White et al. Fig. S1

Supplement: Supplementary file 1 [file emmm0007-0695-sd1.pdf]

**A**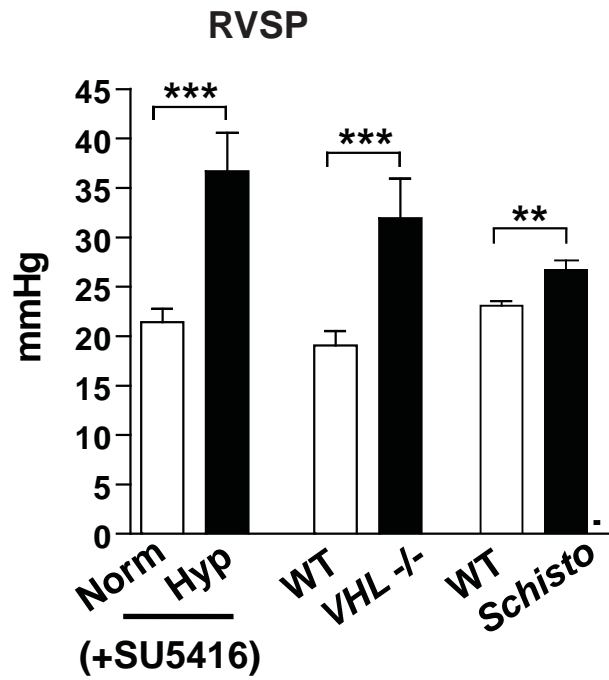**B**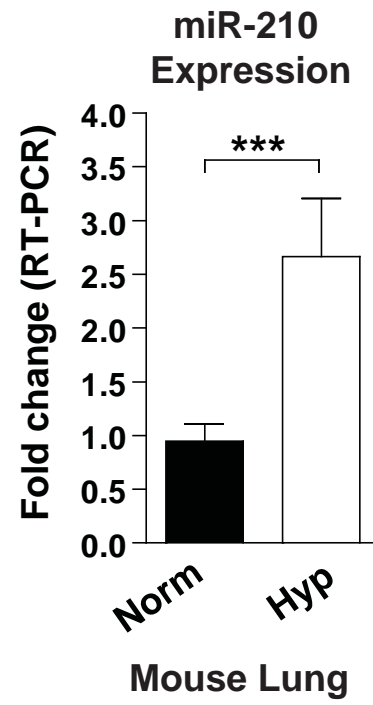

White et al. Fig. S2

Supplement: Supplementary file 2 [file emmm0007-0695-sd2.pdf]

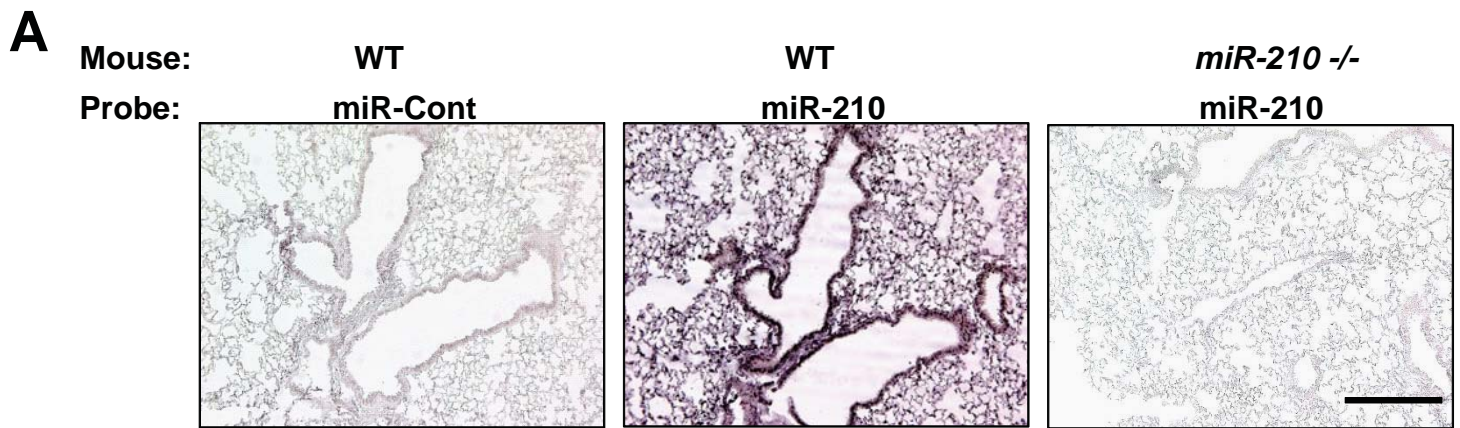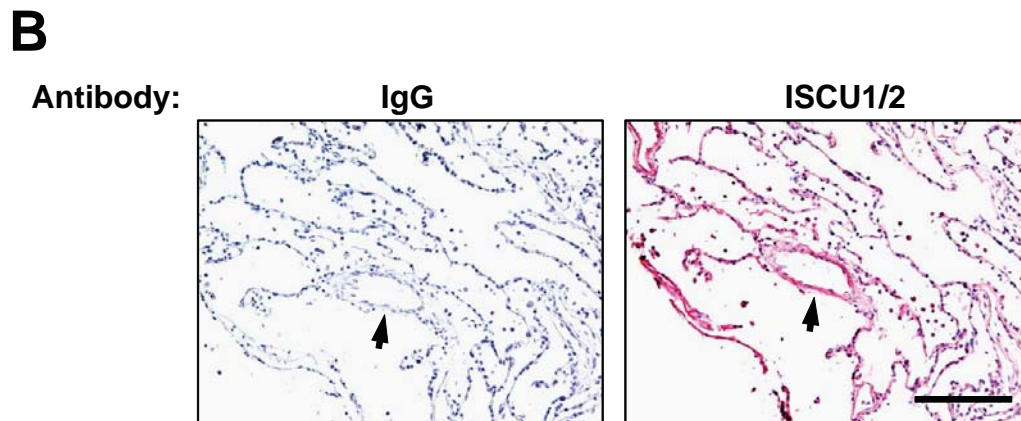

Non-diseased human lung tissue

White et al. Fig. S3

Supplement: Supplementary file 3 [file emmm0007-0695-sd3.pdf]

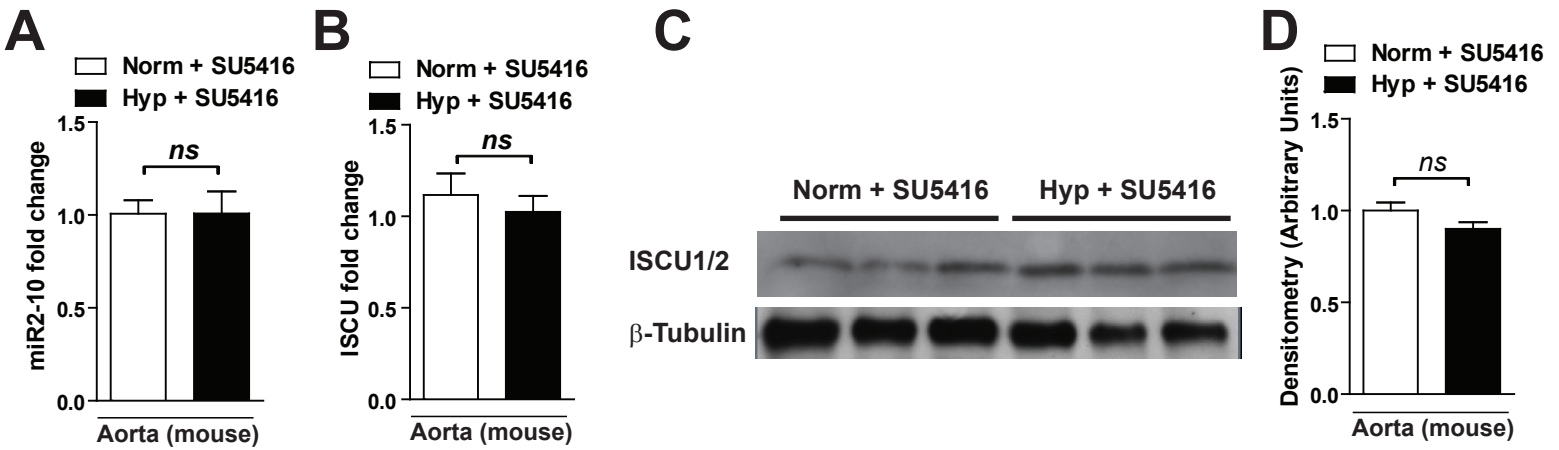

White et al. Fig. S4

Supplement: Supplementary file 4 [file emmm0007-0695-sd4.pdf]

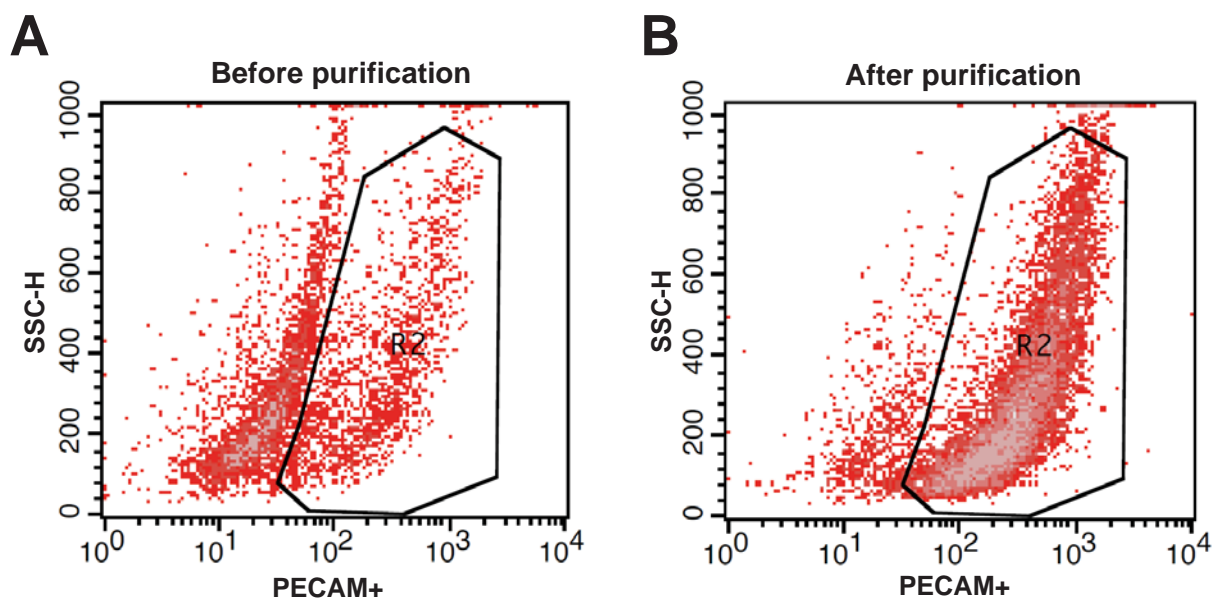

White et al. Fig. S5

Supplement: Supplementary file 5 [file emmm0007-0695-sd5.pdf]

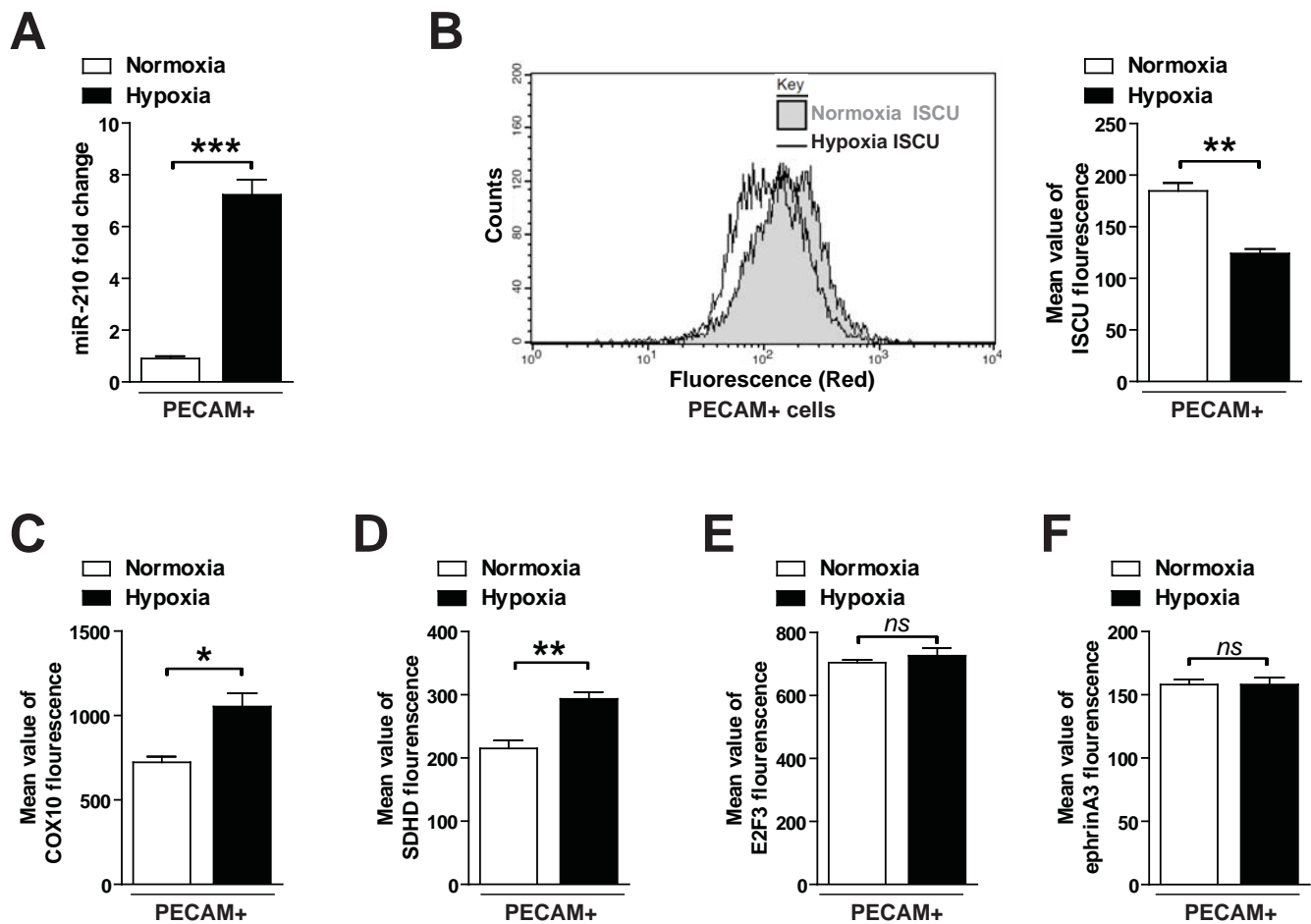

White et al. Fig. S6

Supplement: Supplementary file 6 [file emmm0007-0695-sd6.pdf]

**A**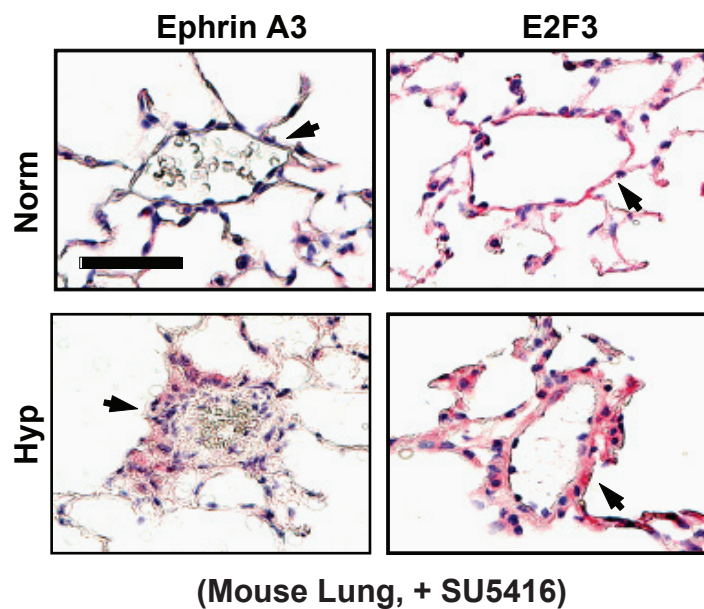**B**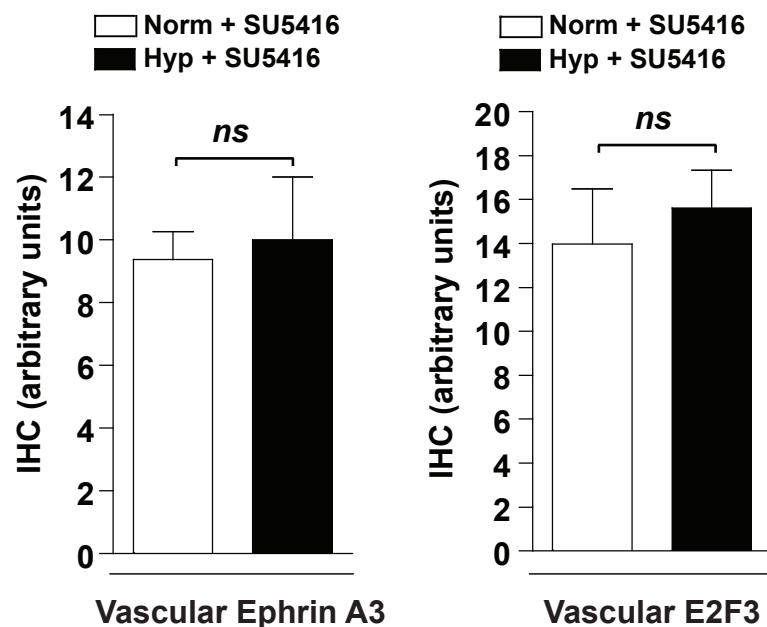**C**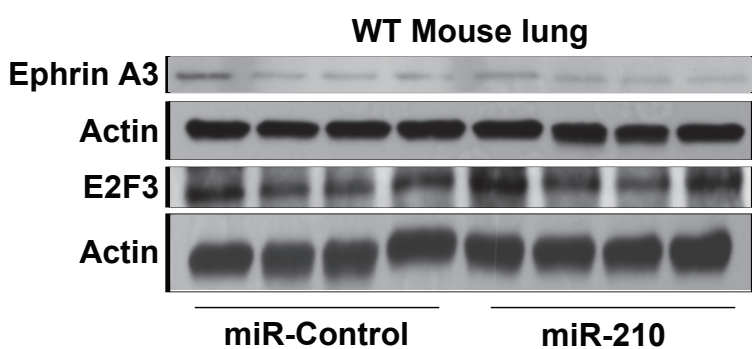**D**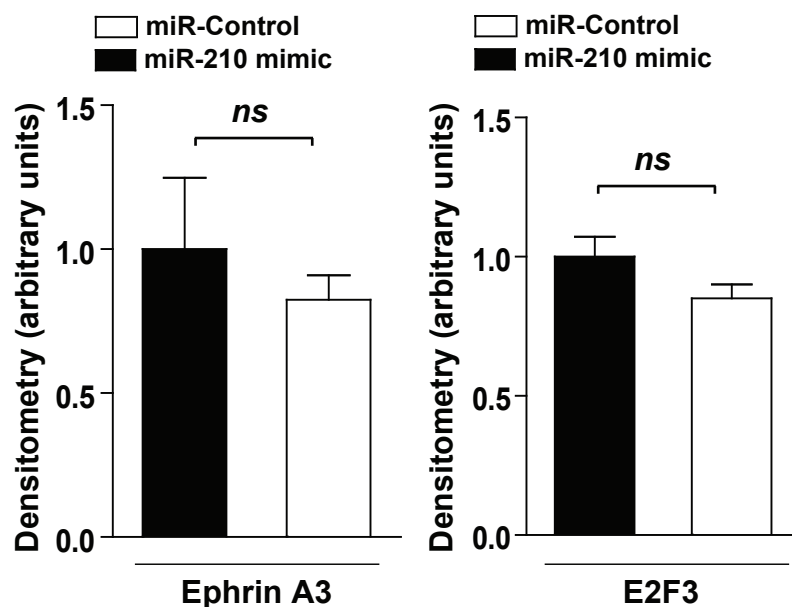**E**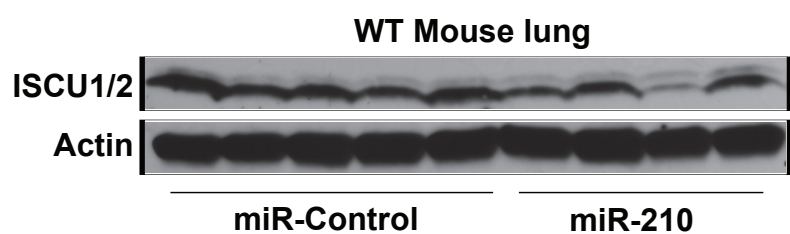**F**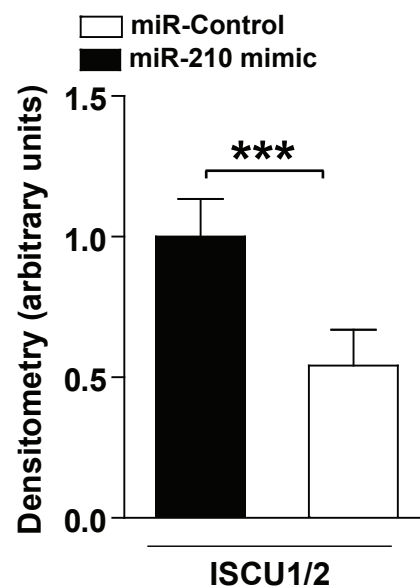

Supplement: Supplementary file 7 [file emmm0007-0695-sd7.pdf]

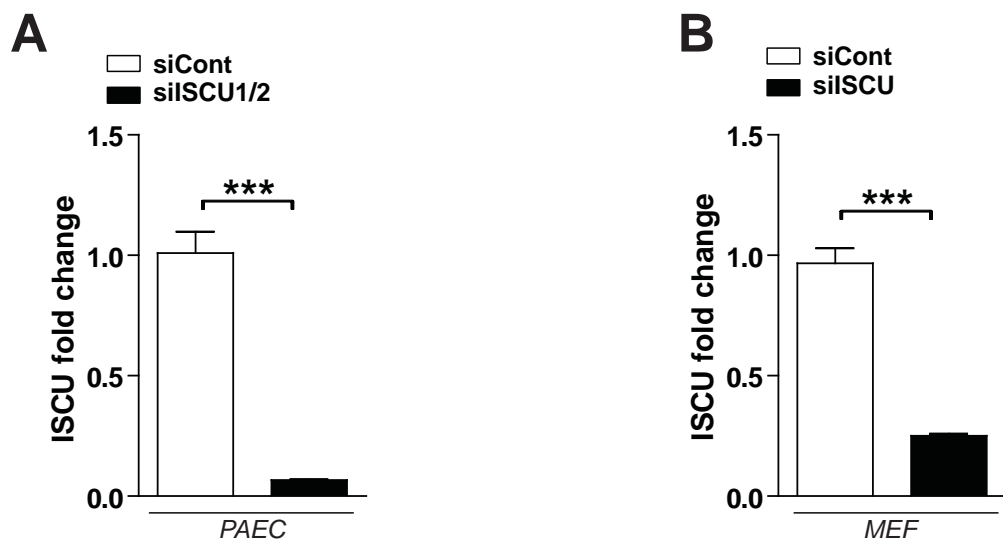

White et al. Fig. S8

Supplement: Supplementary file 8 [file emmm0007-0695-sd8.pdf]

**A**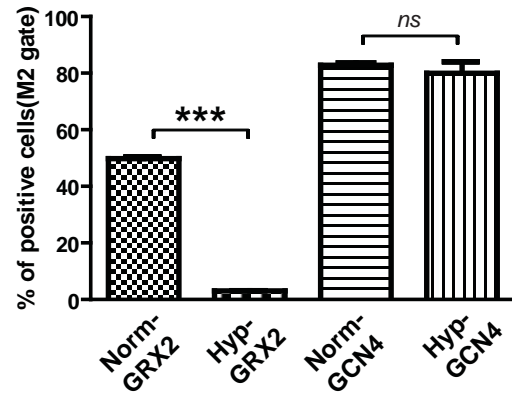**B**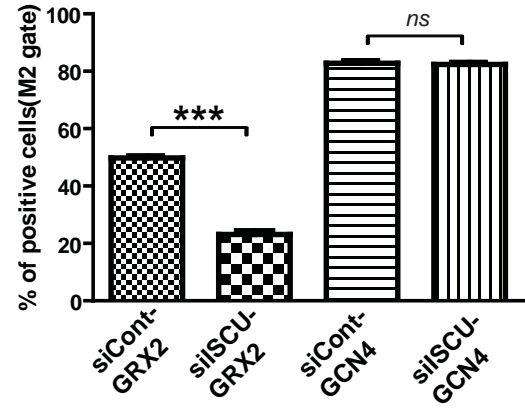**C**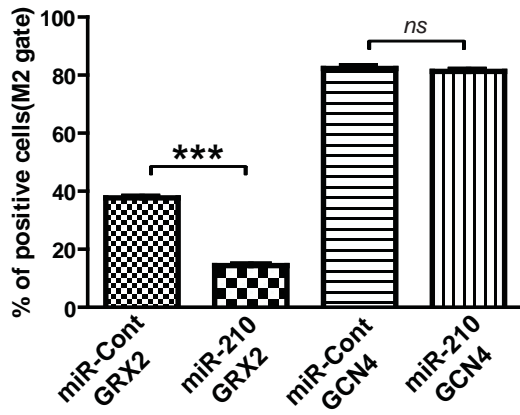**D**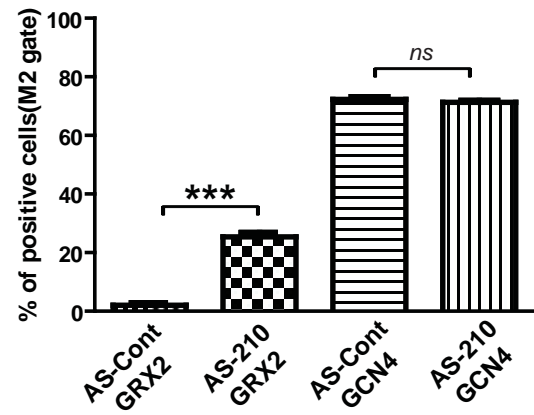

White et al. Fig. S9

Supplement: Supplementary file 9 [file emmm0007-0695-sd9.pdf]

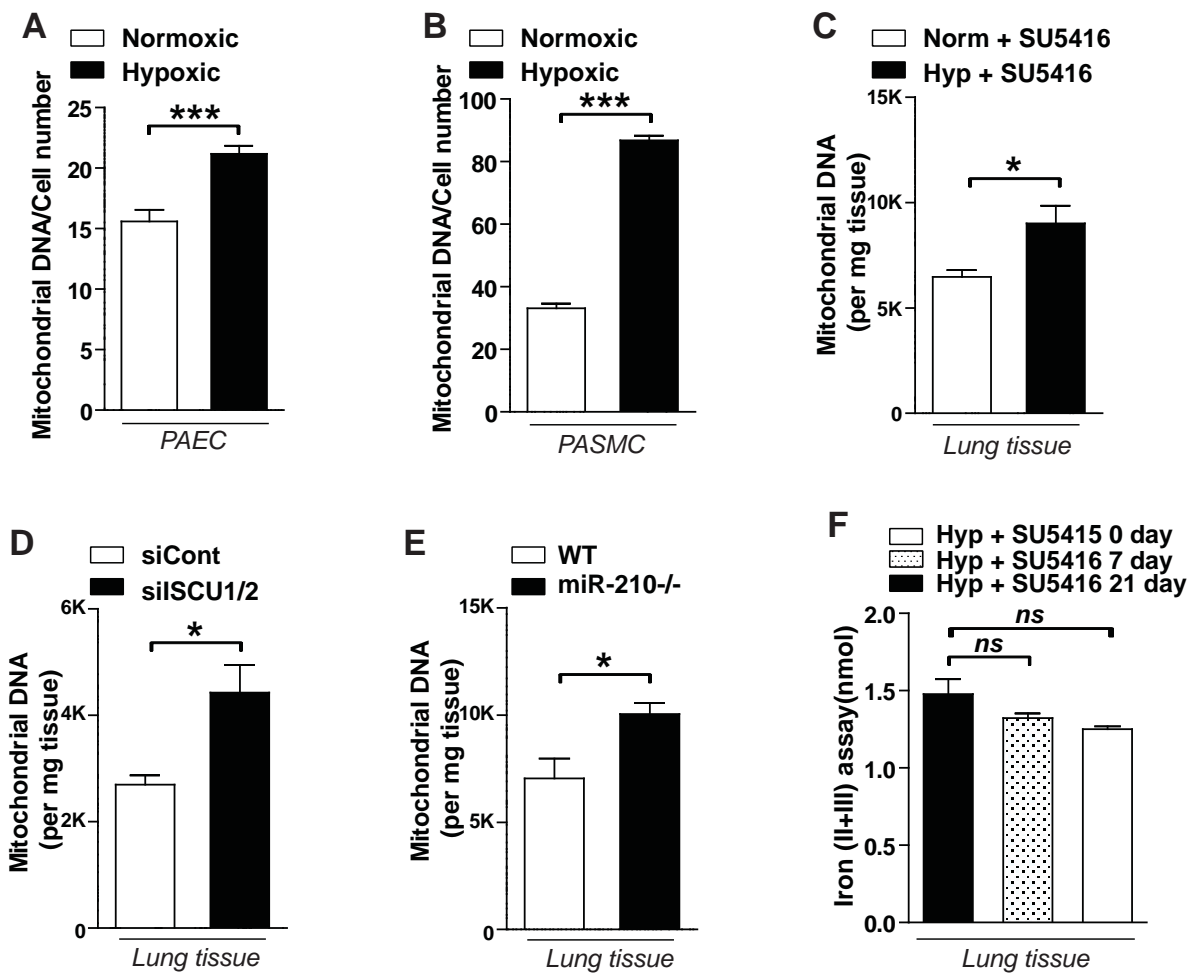

White et al. Fig. S10

Supplement: Supplementary file 10 [file emmm0007-0695-sd10.pdf]

## miR-210 Expression

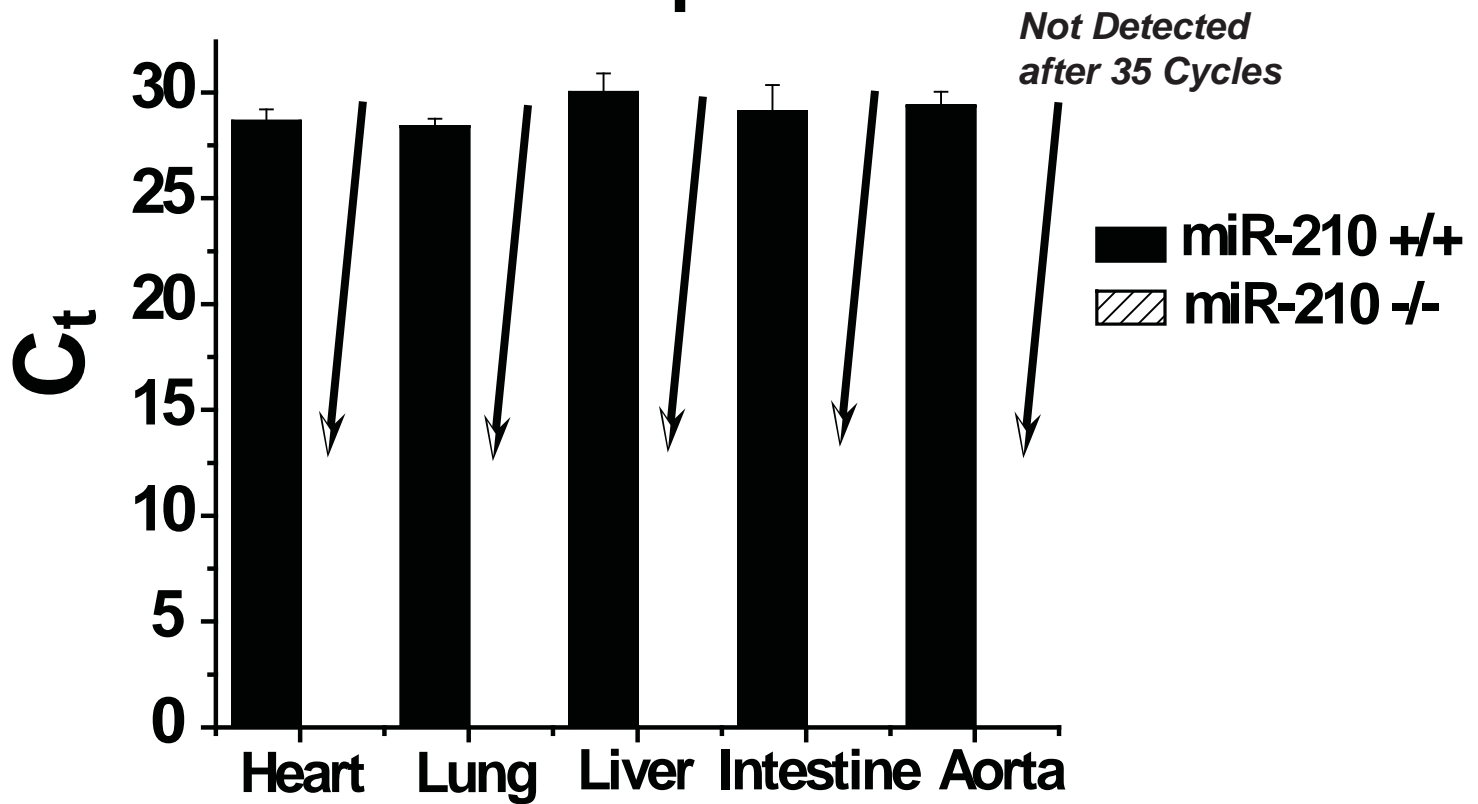

## miR-195 Expression

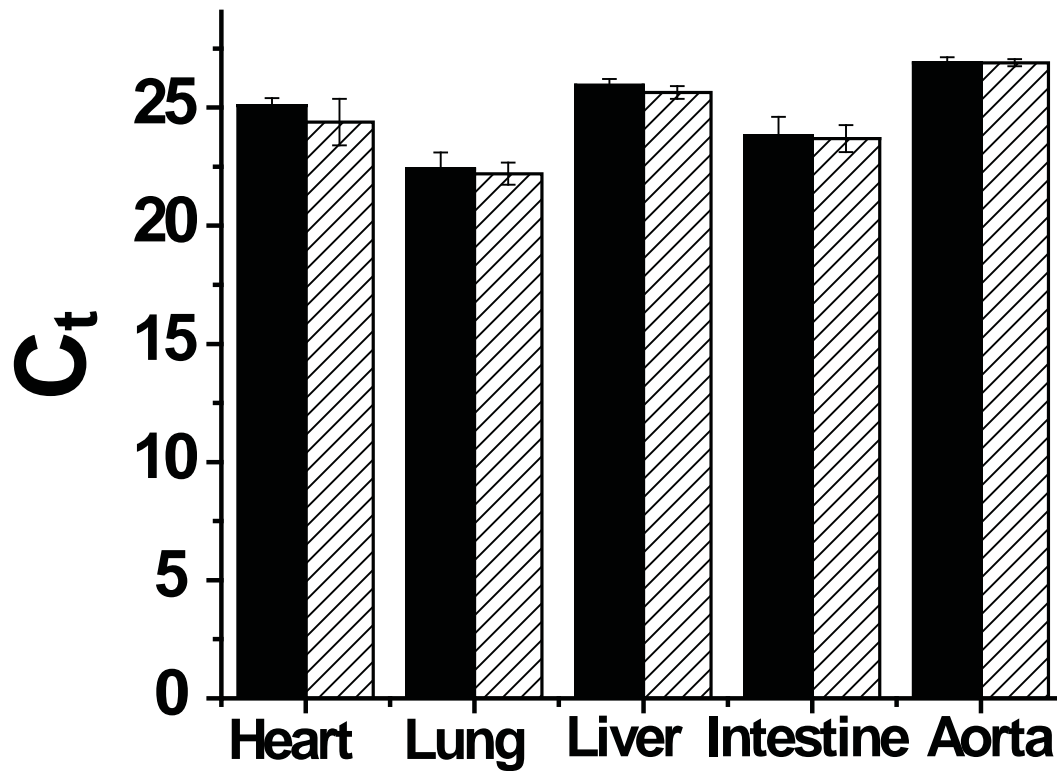

Supplement: Supplementary file 11 [file emmm0007-0695-sd11.pdf]

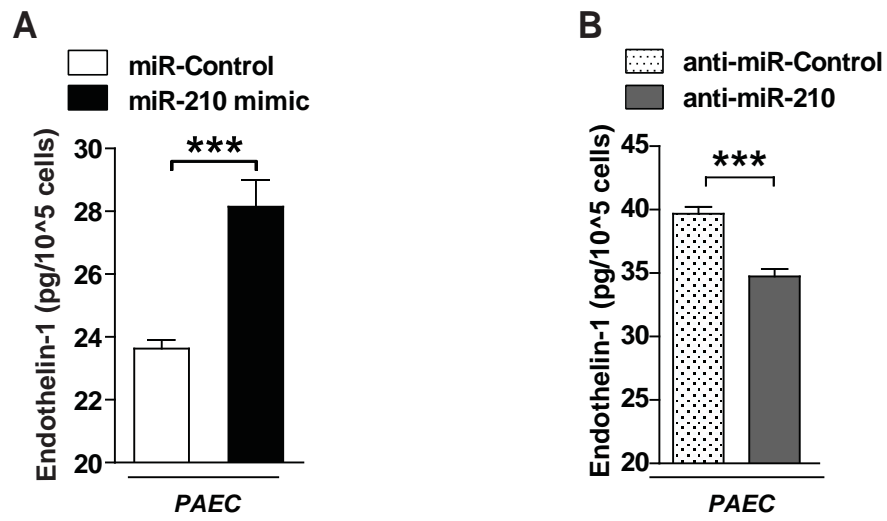

White et al. Fig. S12

Supplement: Supplementary file 12 [file emmm0007-0695-sd12.pdf]

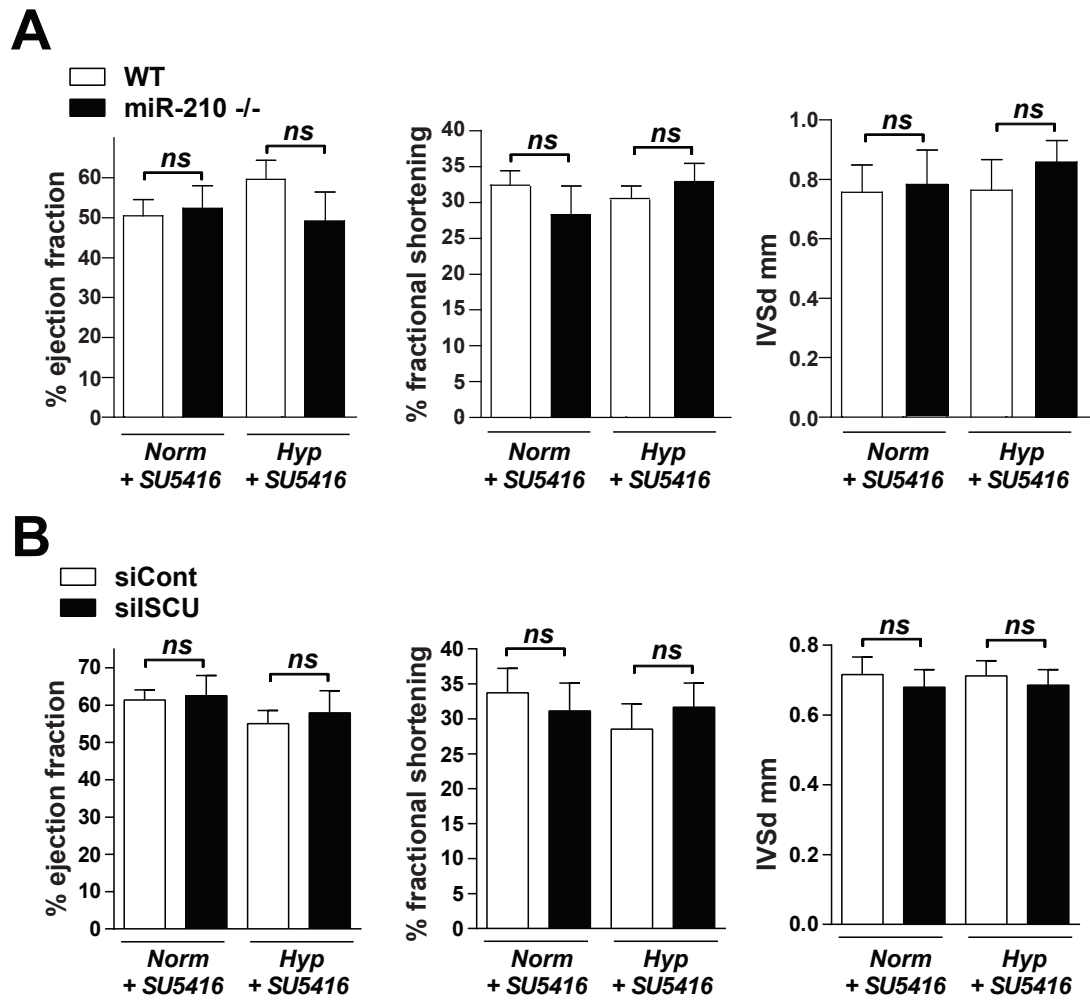

White et al. Fig. S13

Supplement: Supplementary file 13 [file emmm0007-0695-sd13.pdf]

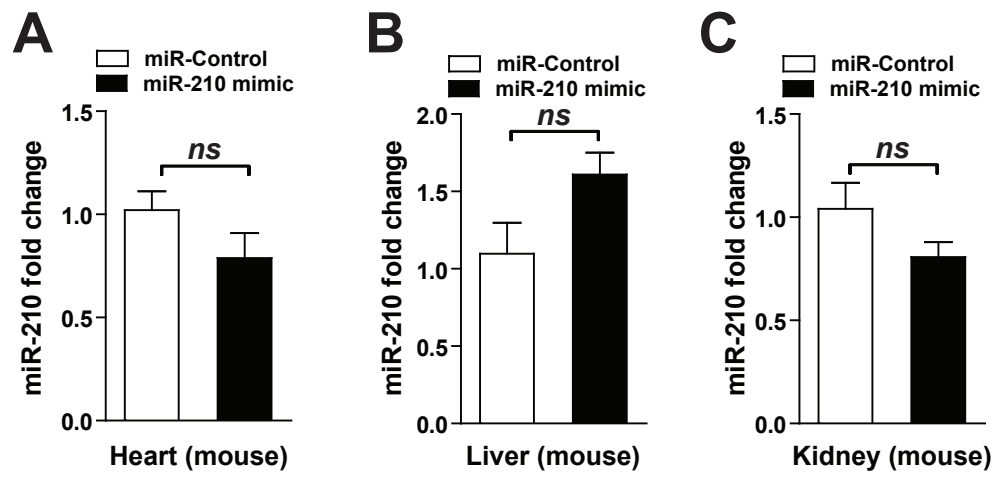

White et al. Fig. S14

Supplement: Supplementary file 14 [file emmm0007-0695-sd14.pdf]

**A**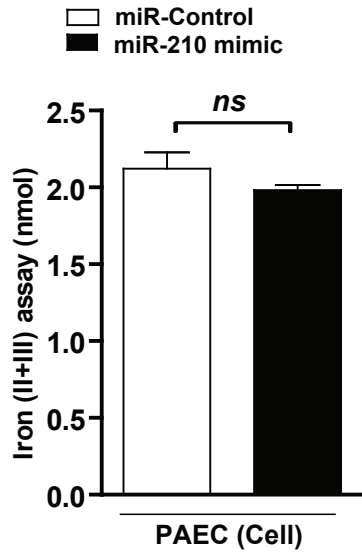**B**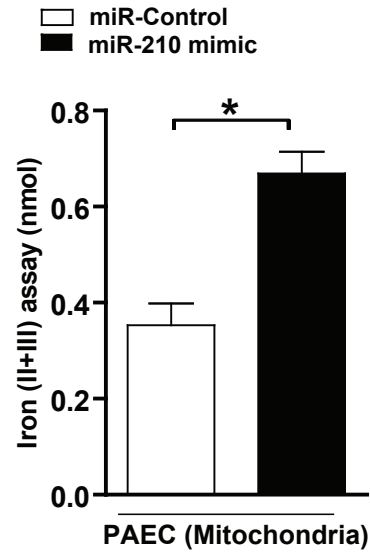**C**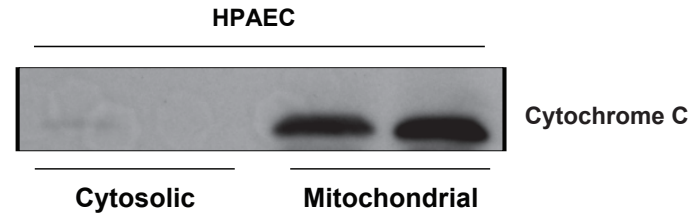

White et al. Fig. S15

Supplement: Supplementary file 15 [file emmm0007-0695-sd15.pdf]

**A**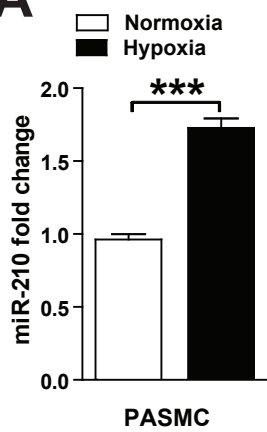**B**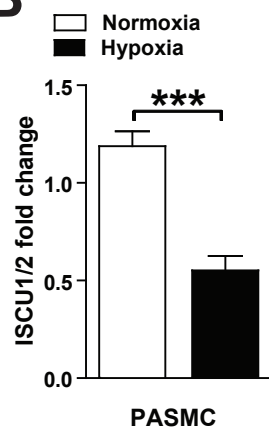**C**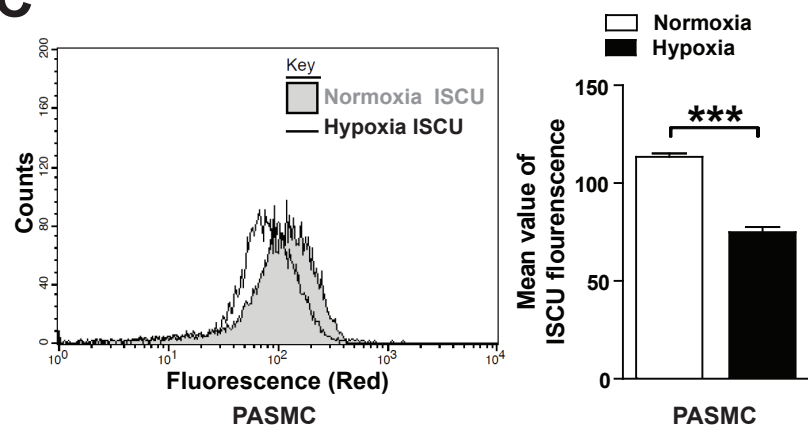

White et al. Fig. S16

Supplement: Supplementary file 16 [file emmm0007-0695-sd16.pdf]

Fig S4-c. ISCU1/2

Norm + SU5416

Hyp + SU5416

ISCU1/2

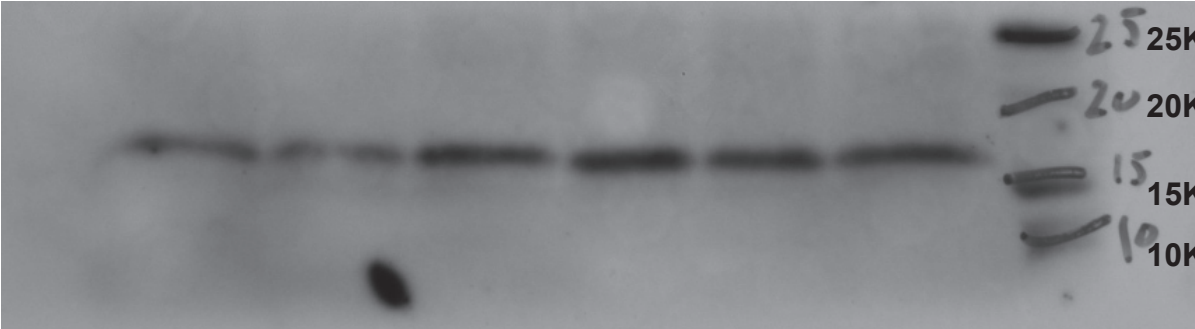

Fig S4-c. TUBULIN

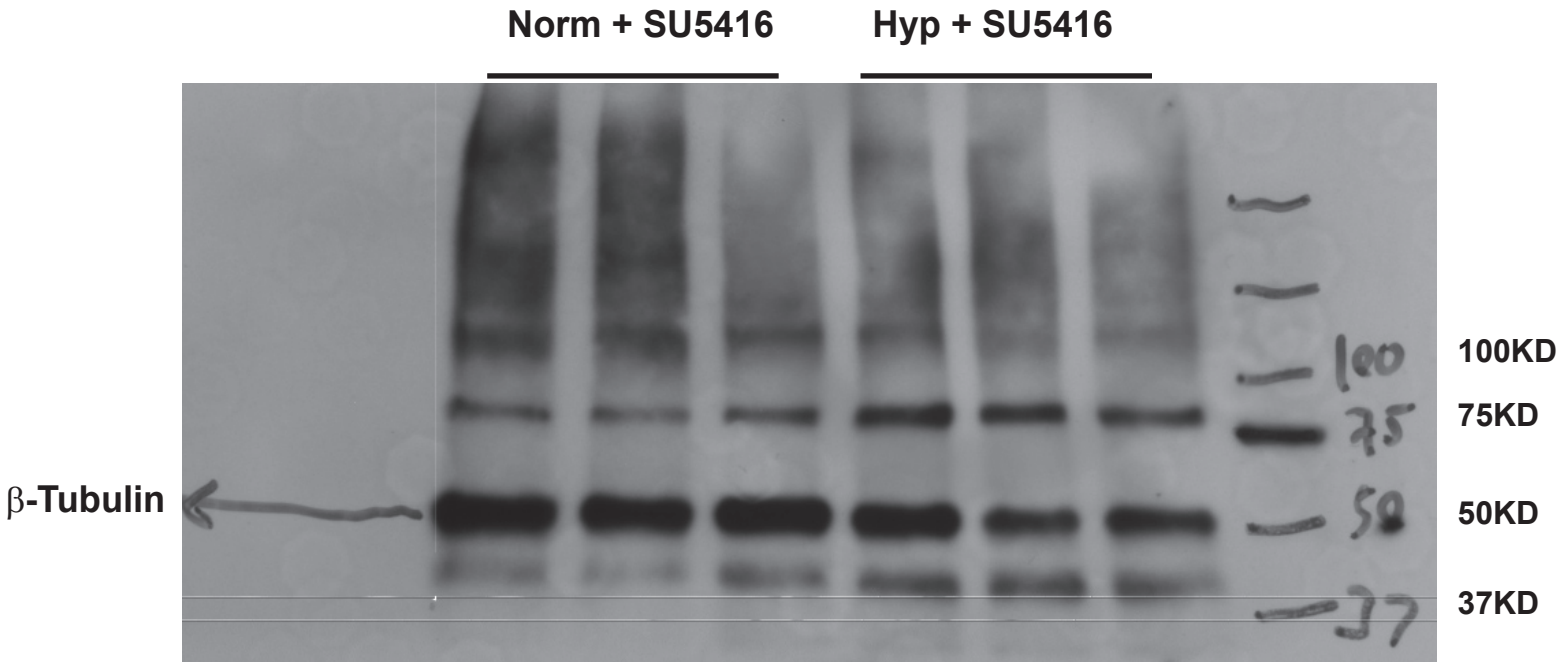

Supplement: Supplementary file 22 [file emmm0007-0695-sd22.pdf]

Fig S7-c. Ephrin A3

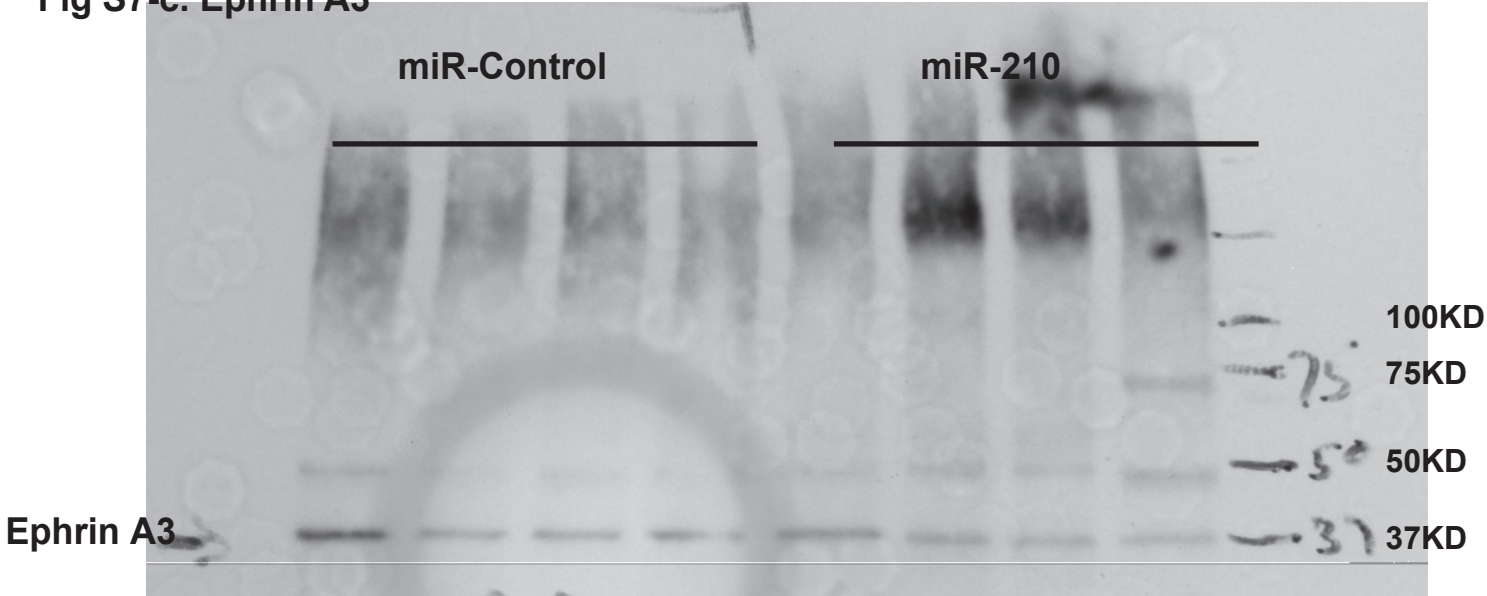

Fig S7-c. ACTIN for Ephrin A3

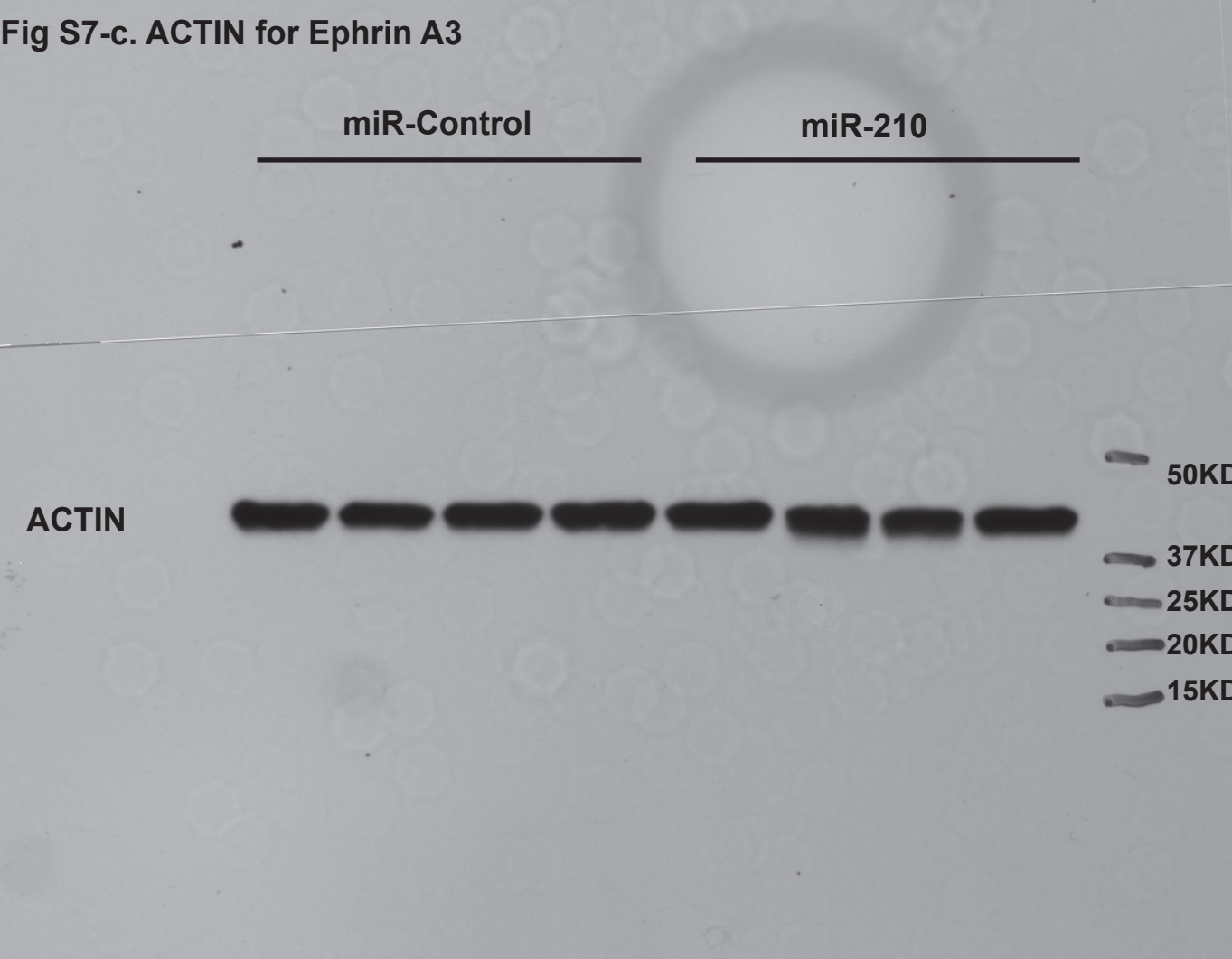

Fig S7-c. E2F3

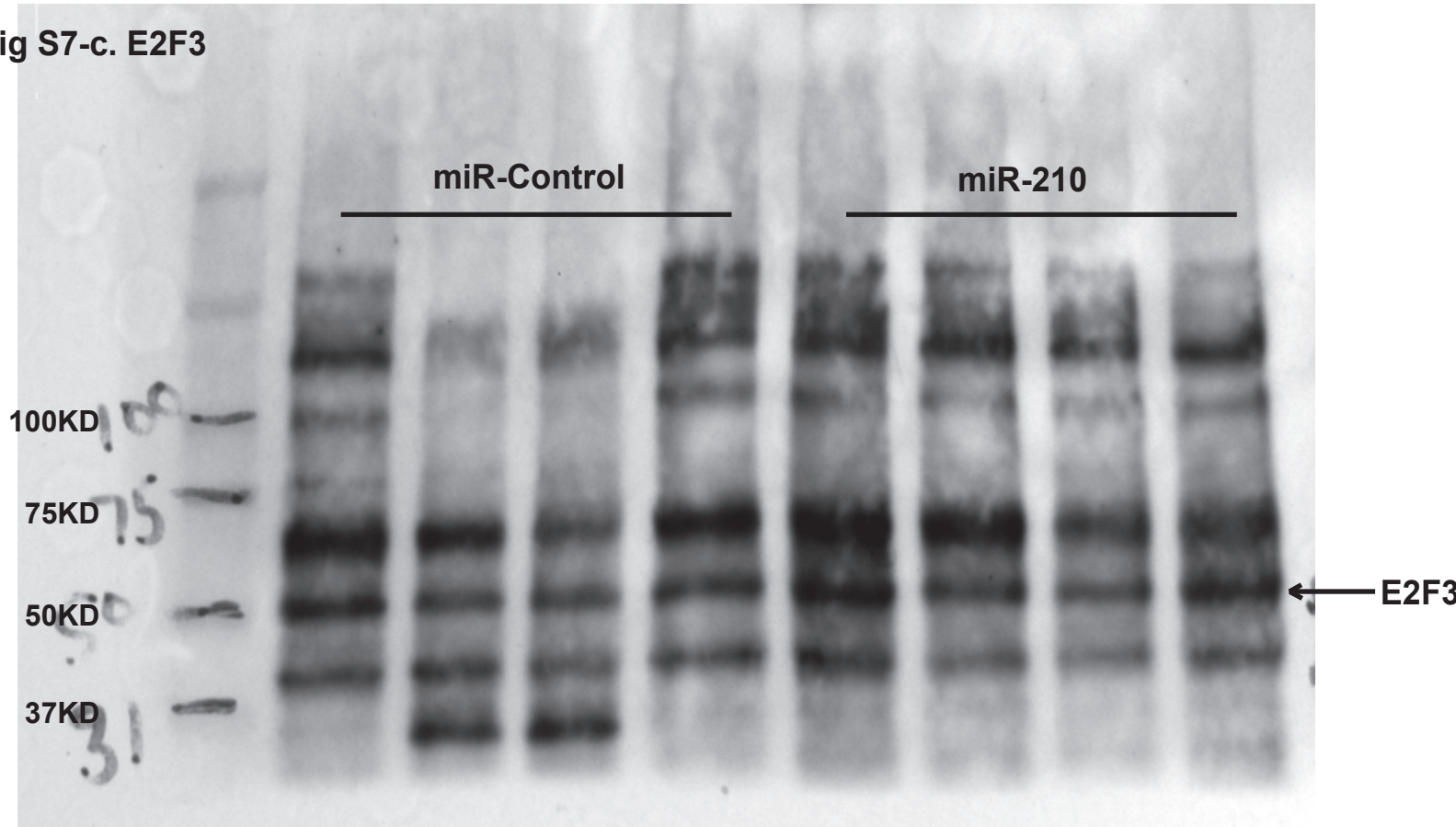

Fig S7-c. ACTIN for E2F3

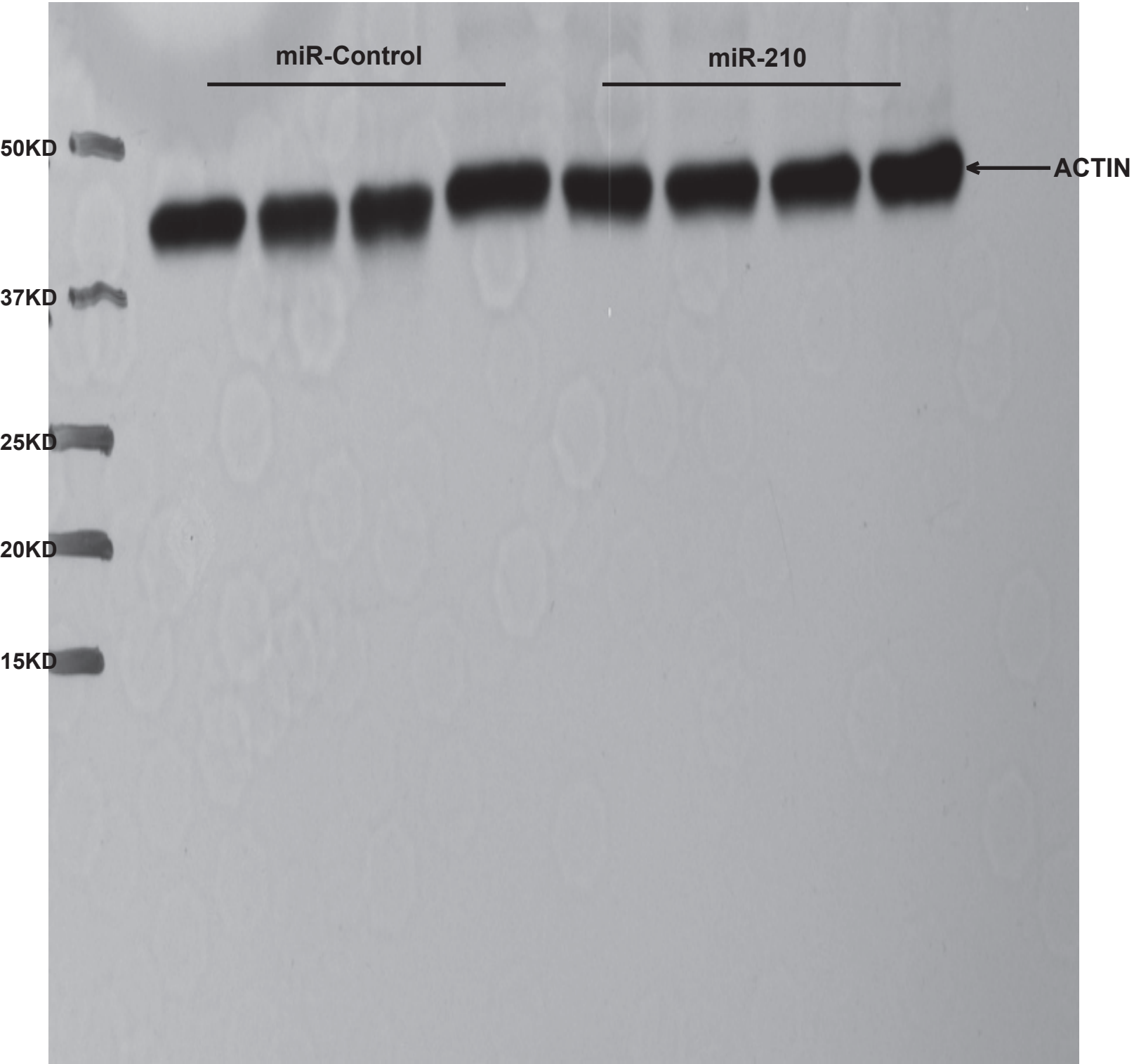

Fig S7-e. ISCU1/2

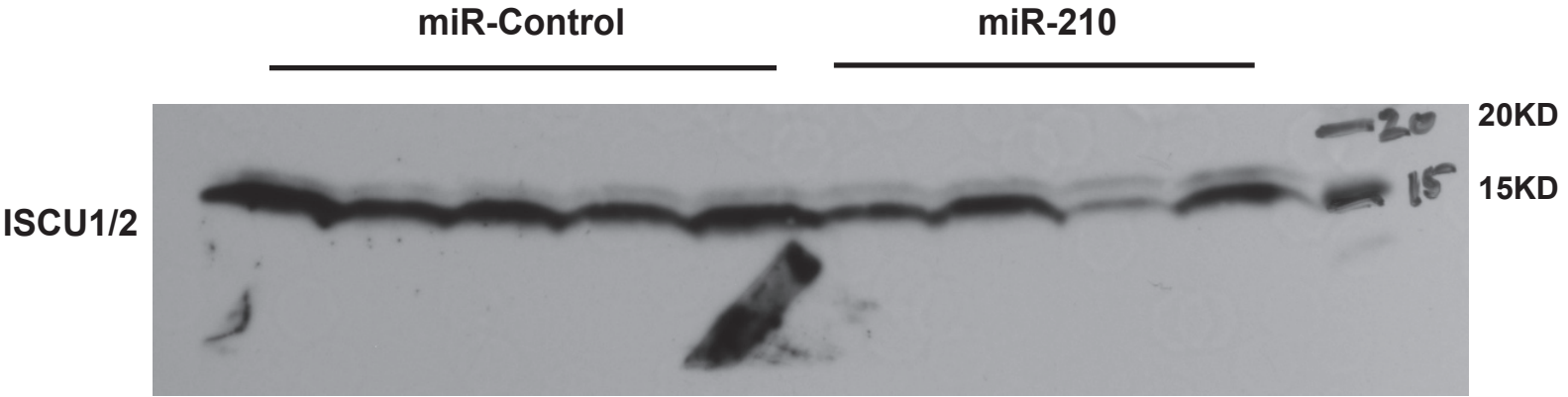

Fig S7-e. ACTIN

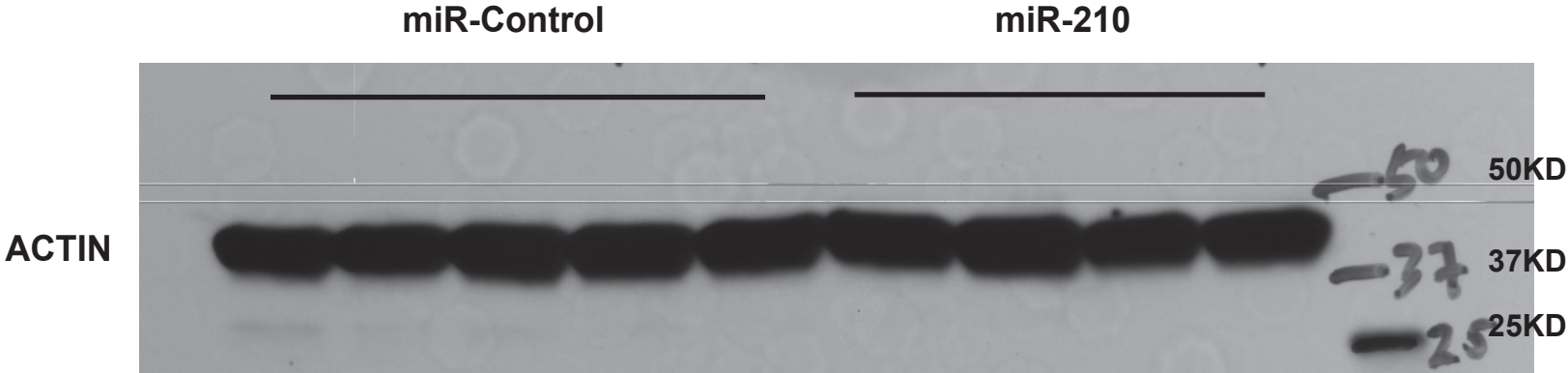

Supplement: Supplementary file 23 [file emmm0007-0695-sd23.pdf]

Fig S15-c. Cytochrome C

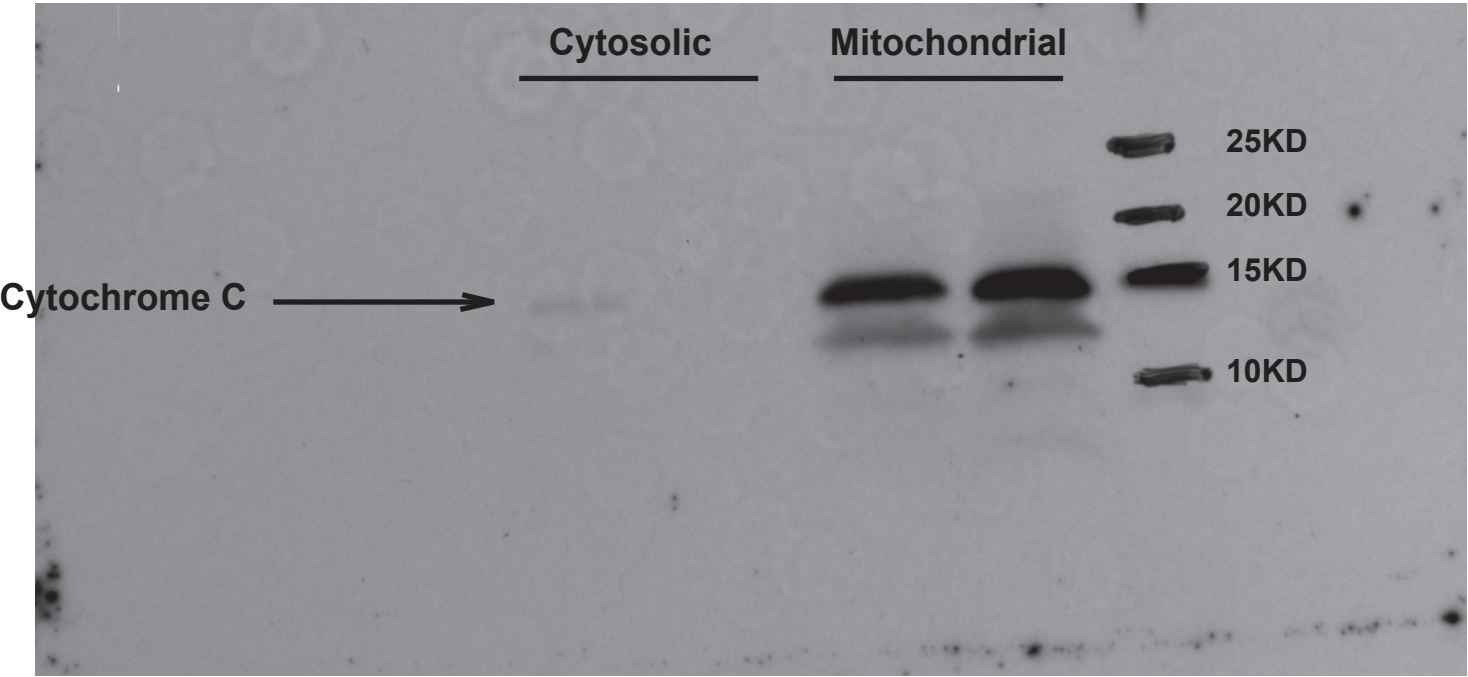

Supplement: Supplementary file 24 [file emmm0007-0695-sd24.pdf]
