# Supplementary material for: Genetic and hypoxic alterations of the microRNA-210-ISCU1/2 axis promote iron–sulfur deficiency and pulmonary hypertension
Source: EMBO Mol Med. 2015 Mar 30;7(6):695–713. doi: 10.15252/emmm.201404511 (PMC4459813; doi:10.15252/emmm.201404511)
Supplement: Supplementary file 17 [file emmm0007-0695-sd17.pdf]

Table S1. Clinical characteristics of PAH patients. These patients were previously described (Bertero et al, 2014).

Patient	Age (yr)	Gender	mPAP (mmHg)	Clinical description
1	34	Female	50	Autopsy, Idiopathic
2	64	Female	55	Autopsy, Idiopathic
3	12	Male	53	Lung transplant, BMPRII mutation
4	16	Male	62	Lung transplant, Idiopathic
5	1	Male	50	Lung resection, Trisomy 21
6	19	Male	48	Lung resection, Idiopathic
**Scleroderma-Induced PAH**				
**Patient**	**Age (yr)**	**Gender**	**mPAP (mmHg)**	**Clinical description**
1	68	Female	44	Lung transplant, Scleroderma
2	56	Male	50	Lung transplant, Scleroderma
3	43	Male	37	Lung transplant, Scleroderma
4	51	Male	48	Lung transplant, Scleroderma
5	59	Female	55	Lung transplant, Scleroderma
6	50	Male	33	Lung transplant, Scleroderma
7	67	Male	50	Lung transplant, Scleroderma
8	39	Male	40	Lung transplant, Scleroderma
9	42	Female	57	Lung transplant, Scleroderma
10	46	Male	47	Lung transplant, Scleroderma
11	60	Female	66	Autopsy, Scleroderma
12	54	Female	54	Autopsy, Scleroderma
13	72	Female	53	Autopsy, Scleroderma
