# Supplementary material for: Genetic and hypoxic alterations of the microRNA-210-ISCU1/2 axis promote iron–sulfur deficiency and pulmonary hypertension
Source: EMBO Mol Med. 2015 Mar 30;7(6):695–713. doi: 10.15252/emmm.201404511 (PMC4459813; doi:10.15252/emmm.201404511)
Supplement: Supplementary file 18 [file emmm0007-0695-sd18.pdf]

**Table S2. Demographic information of 10 human subjects undergoing pulmonary arterial catheterization for clinical dyspnea.** Five subjects were found to have mean pulmonary arterial pressure (PAP) > or = 25 mm Hg (PCWP≤15 mmHg), and five subjects were found to have mean PAP < 25 mm Hg. Plasma samples were drawn at the pulmonary capillary wedge position from which circulating miR-210 levels were measured.

| | **Mean PAP<25 mm Hg** | | **Mean PAP> or = 25 mm Hg** | | |
|---|---|---|---|---|---|
| | Mean | Standard Error | Mean | Standard Error | p-value |
| **Age** | 55.4 | 9.5 | 56 | 8.8 | 0.96 |
| **Systolic blood pressure (mm Hg)** | 138 | 7.5 | 125 | 2.4 | 0.06 |
| **Diastolic blood pressure (mm Hg)** | 79 | 2.8 | 77.8 | 1.6 | 0.96 |
| **Cardiac index (L/min/$m^2$)** | 3.1 | 0.3 | 2.8 | 0.5 | 0.67 |
| **Mean pulmonary arterial pressure (mm Hg)** | 19.6 | 1.7 | 37.4 | 5.7 | 0.02 |
| **Male** | 3/5 | | 1/5 | | |
