# Supplementary material for: Genetic and hypoxic alterations of the microRNA-210-ISCU1/2 axis promote iron–sulfur deficiency and pulmonary hypertension
Source: EMBO Mol Med. 2015 Mar 30;7(6):695–713. doi: 10.15252/emmm.201404511 (PMC4459813; doi:10.15252/emmm.201404511)
Supplement: Supplementary file 19 [file emmm0007-0695-sd19.pdf]

Table S3. Clinical characteristics of PH patients analyzed for pulmonary Fe-S expression

Patient	Age	Gender	Primary lung disease	PAP	Mean PAP
1	70	M	IPF	43/19	29
2	62	M	COPD	38/20	27
3	50	F	Scleroderma	80/24	46

M: Male. F: Female. IPF: Idiopathic Pulmonary Fibrosis. COPD: Chronic Obstructive Pulmonary Disease.
