# Supplementary material for: Genetic and hypoxic alterations of the microRNA-210-ISCU1/2 axis promote iron–sulfur deficiency and pulmonary hypertension
Source: EMBO Mol Med. 2015 Mar 30;7(6):695–713. doi: 10.15252/emmm.201404511 (PMC4459813; doi:10.15252/emmm.201404511)
Supplement: Supplementary file 20 [file emmm0007-0695-sd20.pdf]

Genetic and hypoxic alterations of the microRNA-210-ISCU1/2 axis promote iron-sulfur deficiency and pulmonary hypertension

Supplementary Information

Table of Contents

1. Supplementary Materials and Methods (p. 2-19)
2. Supplementary References (p. 20-21)

Supplementary Materials and Methods

Cell culture

HEK293 cells (ATCC) were cultured in DMEM with 10% fetal calf serum (Life Technologies). Primary human pulmonary arterial endothelial cells (human PAECs, Lonza) and primary human pulmonary microvascular endothelial cells (human pulmonary MVECs, Lonza) were recently authenticated, negative for mycoplasma contamination, and cultured in EGM-2 (Lonza). Primary human pulmonary arterial smooth muscle cells (human PASMCs, Lonza) were also recently authenticated and negative for mycoplasma contamination. Human PAECs, human pulmonary MVECs, and human PASMCs were used for experiments at passages 3-8. Cells were transfected using Lipofectamine 2000 (Thermo Fisher Scientific) per the manufacturer's instructions.

Plasmids

Mammalian expression plasmids encoding GRX2 and GCN4 transgenes fused to the N-terminal or C-terminal portions of the Venus fluorescent protein were provided by Dr. J. Silberg (Rice University), as previously described (Hoff et al, 2009). Transgenes were subcloned into the pCDH-MCS-EF1-PURO lentiviral parent vector (System Biosciences) via BamHI and NotI sites.

Mouse Models

The generation of *mmu-miR-210-/-* mice was described (mixture of C57BL/6N and C57BL/6J Tyr c/c) (Mok et al, 2013), and these mice were a gift from A. Bradley and H. Prosser (Wellcome Trust, U.K.). Multiple mouse models of experimental PH were utilized. First, *VHL* flox/flox;Cre-ER mice (*VHL-/-* mice; >10 backcrosses, C57BL/6) were a gift from W.G. Kaelin

(Dana Farber Cancer Institute, Boston). Conditional inactivation of VHL protein was established by dual injection of tamoxifen (2mg/intraperitoneal/48 hour interval) in 3 week old male *VHL* flox/flox:Cre-ER and wiltype (WT) littermate (*VHL*flox/flox without Cre-ER transgene) mice. Euthanasia and tissue harvest was performed at 10-12 weeks of age. Second, *IL-6* transgenic mice (C57BL/6) have been described (Steiner et al, 2009). Third, to elicit chronic hypoxic PH, C57BL/6 WT littermate male mice (10-12 weeks old, Taconic) were subjected to 21-28 continuous days of normobaric hypoxia in a temperature-humidity controlled chamber (10% O_2, OxyCycler chamber, Biospherix Ltd.) as compared with normoxia (21% O_2). Fourth, to elicit robust PH, WT versus *miR-210-/-* male littermate mice (>6 backcrosses, 10-12 weeks old), were injected weekly (days 0, 7, 14, and 21) with the vascular endothelial growth factor (VEGF) Flk-1/KDR receptor inhibitor SU5416 (20mg/kg/subcutaneous injection, Sigma-Aldrich, as previously described (Ciuclan et al, 2011)) in combination with 28 days of continuous exposure to normobaric hypoxia, as above. Notably, the time of hypoxic exposure varied between 21 and 28 days in wildtype mice administered miRNA or siRNA oligonucleotides, as described below. Fifth, exposure of mice to *Schistosoma mansoni* ova to cause experimental PH was performed using published techniques (Graham et al, 2013a; Graham et al, 2013b) and previously described by our group (Bertero et al, 2014). Upon completion of each of these protocols to induce experimental PH, a combination of transthoracic echocardiography, right heart catheterization, euthanasia, and tissue harvest was performed as described previously (Parikh et al, 2012) and described in detail below.

Exposure to hypoxia

Human PAECs were exposed for 48 hours either to standard non-hypoxic cell-culture conditions,

(20% O_2, 5% CO_2, with N_2 balance at 37°C) or to hypoxia (2% O_2, 5% CO_2, with N_2 balance at 37°C), in a modular hypoxia chamber. Conditions were based on prior studies of human PAECs (Chan et al, 2009; Manalo et al, 2005) to allow for steady-state adaptation without non-specific cell death. After fixation and permeabilization by Cytofix/Cytoperm kit (BD), human PAECs were stained by polyclonal rabbit anti-HIF-1α (Novus, 1:500); polyclonal rabbit anti-HIF-2α (Novus, 1:500) overnight. After washing by PBS, the cells were stained by PE-anti-Rabbit-IgG antibody 1:2000 (Santa Cruz Biotechnology) and were tested by FACScan (BD Biosciences).

Immunoblotting

Immunoblotting was performed as described (Chan et al, 2009), after preparation of extracts in RIPA lysis buffer (Sigma-Aldrich). Primary antibodies used in this study were polyclonal rabbit anti-ISCU (Santa Cruz, 1:1000); polyclonal rabbit anti-β-tublin (Santa Cruz, 1:1000); polyclonal rabbit anti-Ephrin-A3 (Santa Cruz, 1:1000); polyclonal rabbit anti-E2F3 (Santa Cruz, 1:1000); polyclonal rabbit anti-cytochrome C (Santa Cruz, 1:1000); monoclonal mouse anti-FLAG (Sigma-Aldrich, 1:5000); polyclonal rabbit anti-actin (Sigma-Aldrich, 1:3000). Secondary antibodies included goat anti-mouse HRP and goat anti-rabbit HRP (Santa Cruz Biotechnology, 1:3000).

Lentivirus production and infection of human PAECs

HEK293 cells were transfected using Lipofectamine 2000 (Thermo Fisher Scientific) with lentiviral plasmids along with packaging plasmids (pPACK, System Biosciences), per manufacturer's instructions. Virus was sterile filtered (0.2 μm) and utilized for infection of

human PAECs for gene transduction.

Electron paramagnetic resonance spectroscopic analysis of Fe-S clusters in human lung

Electron paramagnetic resonance (EPR) spectroscopy (Bruker ESP-300E EPR spectrometer equipped with an ER-4102ST cavity and an EIP Model 28B frequency counter) was utilized to quantitatively analyze Fe-S cluster levels in PH and control lung tissues (300 mg/patient), flash-frozen from human patients. All spectra were recorded at $15^{o}K$ with the following parameters: microwave frequency ~9.46 GHz, microwave power = 1.0 mW, modulation amplitude = 5.0 G, modulation frequency = 100 kHz, time constant = 163.84 ms, and scan number = 10. The peak-to-peak amplitude of the tissue Fe-S EPR signal centered at approximately g = 1.93 (corresponding to a magnetic field of ~ 3505 Gauss) was measured. This signal is unique by virtue of the fact that it presents upfield from other paramagnetic centers that have $g > 2.0$. It is accepted that this signal primarily represents [2Fe-2S] and [4Fe- 4S] clusters, as we described (Chan et al, 2009).

Plasma RNA extraction

Plasma sampling from human patients was performed as previously described (Baggish et al, 2011). To remove any residual cellular contents, thawed plasma samples were further centrifuged (13,000 rpm x 10 minutes), and plasma supernatant was aliquoted into 100 μl volumes. To a standard volume of each plasma supernatant (100 μl), 2 femtomoles were added of a chemically synthesized microRNA duplex mimic of microRNA-422b (miR-422b) (Life Technologies). Given minimal expression and minimal changes of expression of endogenous miR-422b as determined by empiric observation (SY Chan, unpublished data, 2010), equivalent levels of exogenously added miR-422b were used for quantitative normalization of microRNA

plasma levels, as previously described (Baggish et al, 2011). Total RNA extraction was performed using a microRNA extraction kit (Benevbio).

RT-PCR

Taqman RT-PCR analysis was performed on flash-frozen lung tissues and plasma tissues, as described (Parikh et al, 2012). Briefly, RNA extraction (RNeasy mini kit, Qiagen) was followed by measuring total RNA concentration by Nanodrop spectrophotometry (ThermoScientific). Reverse transcription of 10ng of total RNA was performed to generate cDNA representing levels of mature microRNA molecules (Taqman MicroRNA Reverse Transcription Kit, Life Technologies). Owing to the stem loop structures of the microRNA primers, only mature microRNA molecules were amplified into cDNA. cDNA was amplified via fluorescently labeled Taqman probe and primer sets using an Applied Biosystems 7900HT Fast Real Time PCR device. Fold-change of RNA species was calculated using the formula 2 ($-\Delta\Delta Ct$) with a control level that was normalized to fold-change of 1. As reference controls for microRNA lung quantification in human tissue, the small RNA, RNU48, was used; as reference controls for microRNA lung quantification in mouse tissue, endogenous miR-16 was used, given empiric observations of its consistent expression in hypoxia and models of PH (Parikh et al, 2012). As reference controls for microRNA plasma quantification, the spike-in control miR-422b was used.

Quantification of mitochondrial DNA levels

Flash frozen mouse lung tissue (normalized by mass) or cultured human PAECs (normalized by cell number) were homogenized and lysed in Cytosol Extraction Buffer (Abcam), followed by mitochondrial DNA extraction via the Mitochondrial DNA Isolation Kit (Abcam). DNA

concentrations were measured by NanoDrop DN-1000 spectrophotometer (NanoDrop Technologies).

Quantification of iron in lung tissue and mitochondrial fractions versus total cell lysates of PAECs

Flash frozen mouse lung tissue were homogenized and lysed in 1% (v/v) lauryl maltoside (Abcam) in sterile PBS (pH7.4, Sigma-Aldrich), followed by measurement of protein in lysates by DC protein assay (Biorad). 80 µg of total protein was loaded into each well for iron (II+III), per the QuantiChromTM Iron Assay Kit (DIFE-250) instructions (BioAssay Systems). Similarly, cultured PAECs previously transfected with miR-210 oligonucleotide mimics versus control mimics for 48 hours as described above were lysed in % (v/v) lauryl maltoside (Abcam) in sterile PBS (pH7.4, Sigma-Aldrich), followed by measurement of protein in lysates by DC protein assay (Biorad). Separately, mitochondrial fractions were extracted from total cell lysates using Mitochondrial Isolation Kit for Mammalian Cells (Thermo Fisher Scientific, #89874). Total cell lysates and mitochondrial fractions normalized by protein content were then assayed for iron (II+III) levels per the QuantiChromTM Iron Assay Kit (DIFE-250) instructions (BioAssay Systems).

Quantification of endothelin-1 by ELISA

Flash frozen mouse lung tissue or human PAECs were homogenized and lysed in 1% (v/v) lauryl maltoside (Abcam) in sterile PBS (pH7.4, Sigma-Aldrich), followed by measurement of protein in lysates by DC protein assay (Biorad). 20 µg of total protein was loaded into each well for mature endothelin-1 immunocapture, per the manufacturer's instructions (Enzo Life Sciences).

Immunocaptured mature endothelin-1 protein concentration was quantitatively determined in each sample by DC protein assay for calculation of specific activity. Separately, after washing, mature endothelin-1 activity was measured by spectrophotometry (A_{340nm}).

Transthoracic echocardiography and right heart catheterization of mice

Transthoracic echocardiography was performed on conscious mice using a 28 MHz linear array transducer connected to a digital ultrasound console (Vevo2100 Visualsonics), as previously described for mice (Bauer et al, 2013). Right heart catheterization and measurement of right ventricular systolic pressure (RVSP) were performed as previously described for mice (Song et al, 2008).

Tissue harvest from mice

After physiological measurements, blood was extracted from mice by cardiac puncture, as described (Minamishima et al, 2008). By right ventricular puncture, the pulmonary vessels were gently flushed with 1 cc of saline to remove the majority of blood cells, prior to harvesting cardiopulmonary tissue. Organs were then harvested for histological preparation (see below) or flash frozen in liquid nitrogen for subsequent homogenization and extraction of RNA and/or protein.

Isolation of mouse pulmonary vascular endothelial cells

Up to three lobes of lung tissue from a mouse were diced with scissors, to which 100 μL of collagenase D solution (Sigma) were added for a final concentration of 2 mg/ml collagenase D in 4.9 mL HEPES buffer (pH=7.4). After incubation for 30 min at 37°C with automated rotation, 20

μL of DNase I (Sigma, final concentration of 80 U/mL DNase I) was added and incubated on ice for 30 min. The solution was filtered twice by a 70 μm cell strainer (BD Biosciences) to yield a single cell suspension. After two rounds of PBS wash, cell pelleting, and resuspension, the ACK lysing buffer (Life Technologies) was used to remove erythrocytes. Remaining cells were incubated with anti-CD31 (PECAM) MicroBeads (Miltenyi Biotec) and with FITC-conjugated anti-CD31 (PECAM) antibody (Abcam; Ab95692). Single PECAM-positive cells were then collected using an autoMACS Pro Separator, per the manufacturer's instructions (Miltenyi Biotec). The purity of PECAM+ cells was confirmed by flow cytometric analysis of FITC-positive cells by a FACScan flow cytometer (BD Biosciences). After fixation and permeabilization by Cytofix/Cytoperm kit (BD), the PECAM-positive cells were stained by polyclonal rabbit anti-COX10 (Santa Cruz, 1:500); polyclonal rabbit anti-SDHD (Santa Cruz, 1:500); polyclonal rabbit anti-E2F3 (Santa Cruz, 1:500); polyclonal rabbit anti-Ephrin-A3 (Santa Cruz, 1:500); anti-ISCU antibody 1:500 (Santa Cruz Biotechnology) overnight. After washing by PBS, the cells were stained by PE-anti-Rabbit-IgG antibody 1:2000 (Santa Cruz Biotechnology) and were tested by FACScan (BD Biosciences).

In situ *hybridization, immunohistochemistry, and immunofluorescence*

To process lung tissue, prior to excision, lungs were flushed with PBS at constant low pressure (~10mmHg) via right ventricular cannulation, prior to tracheal inflation of the left lung with 10% neutral-buffered formalin (Sigma-Aldrich) at a pressure of ~20cm H_2O. After 16 hours of fixation in 10% neutral-buffered formalin at 25°C, lung tissues were paraffin embedded via an ethanol-xylene dehydration series, before being sliced into 5μm sections (Hypercenter XP System and Embedding Center, Shandon). Immunohistochemistry was performed as previously

described (Parikh et al, 2012). Briefly, high temperature citrate buffer antigen-retrieval was performed followed by 10% (v/v) normal goat serum block. Primary antibodies [anti-alpha smooth muscle actin monoclonal antibody (1:500, Sigma-Aldrich), anti-ISCU1/2 polyclonal antibody (1:250, Santa Cruz Biotechnology), anti-GLUT1 polyclonal antibody (1:500, Santa Cruz Biotechnology), anti-3-nitrotyrosine polyclonal antibody (1:1000, EMD Millipore), anti-ephrin A3 polyclonal antibody (1:500, Santa Cruz Biotechnology), anti-E2F3 polyclonal antibody (1:500, Santa Cruz Biotechnology)] or isotype IgG control (desired concentration, Vector Labs) were incubated overnight at 4°C followed by Tris-buffered saline wash and incubation with biotinylated secondary antibody (Vector Labs) for 1hr at 25°C. Color formation was achieved by incubation with streptavidin-biotinylated alkaline phosphatase complex (Vector Labs) followed with alkaline phosphatase substrate solution (Vector Labs). Levamisole (Vector Labs) was added to inhibit endogenous alkaline phosphatase activity. To improve visualization of lung architecture, lung tissues were counterstained with Harris modified hematoxylin (Sigma-Aldrich). Photomicrographs were obtained using an Olympus Bx51 microscope. In concert with specific antibody stains, identification of muscularized pulmonary vessels was accomplished by α-SMA stain of adjacent (5 μm) tissue sections. Intensity of staining was quantified using Adobe Photoshop CS3 Extended (Adobe) in a blinded fashion. Analysis was performed by inversion of photomicrographs, and selection and highlight of two representative opposing areas within the pulmonary vascular wall to generate a mean intensity value between 0 and 255 (A.U.) per square unit of tissue in the vascular wall. Degree of pulmonary arteriolar muscularization was assessed in paraffin-embedded lung sections stained for α-SMA by calculation of the proportion of fully and partially muscularized peripheral (<100 μm diameter) pulmonary arterioles to total peripheral pulmonary arterioles, as previously described (Bertero et al, 2014). A minimum of 10

resistance pulmonary arteries (<100 μm external diameter; confirmed by non-association with bronchial airways) were analyzed per animal, and a minimum of 5 small pulmonary arteries (<200 μm external diameter; confirmed by non-association with bronchial airways) were analyzed per patient specimen.

The scoring of pulmonary vascular pathology was assessed in diseased pulmonary hypertensive lung tissues by a blinded investigator. Following α-SMA staining of 5μm lung sections to visualize the pulmonary vasculature, the ratio of vessel wall thickness: vessel diameter was utilized as an index to accurately assess the extent of vascular remodeling in resistance pulmonary arteries. A minimum of 10 resistance pulmonary arteries were quantitatively analyzed per mouse.

Immunofluorescence was utilized to visualize nuclear expression of proliferating cell nuclear antigen (PCNA) in lung tissue sections. After high temperature citrate buffer antigen-retrieval and 10% (v/v) normal goat serum block, tissue slides were incubated at 4^oC with anti-PCNA monoclonal antibody (1:1000, Abcam) or IgG control (equivalent concentration, Vector Labs). A PBS wash was then performed, followed by incubation with Alexa Fluor® 488 secondary antibody (Life Technologies) for 1hr at 25^oC. To visualize nuclei, lung tissues were counterstained with DAPI (Vector Labs). As above, identification of pulmonary vessels was accomplished by immunohistochemical stain of α-SMA in an adjacent (5 μm) tissue section. Analysis was performed in photomicrographs (Olympus Fluoview 1000) by quantitative assessment of PCNA-positive (PCNA+) cells in the pulmonary vascular wall versus the total number of DAPI-positive (DAPI+) cells, and expressed as a percentage. A minimum of 5

resistance pulmonary arteries (<100 µm external diameter; confirmed by non-association with bronchial airways) were analyzed per animal.

In situ microRNA analysis was utilized to visualize miR-210 in paraffin-embedded 5µm lung tissue sections, as described (Parikh et al, 2012). Tissues were digested with proteinase K (Sigma-Aldrich) and N-(3-Dimethylaminopropyl)-N′-ethylcarbodiimide hydrochloride (EDC; Sigma-Aldrich) prior to incubation with 3'-FITC-labeled miRCURY LNA hsa-miR-210 detection probe (100nmol; Exiqon) or FITC-labeled miRCURY LNA hsa-miR-control (100nmol; Exiqon) for 16 hours at 60°C. Color formation was achieved by incubation with anti-FITC horseradish peroxidase-conjugated secondary antibody (1:500, DAKO) for 1 hour at 25°C, followed by incubation with the chromagen Nitro blue tetrazolium chloride/5-Bromo–4-chloro–3–indolyl phosphate (NBT/BCIP, Roche). Similar to quantification of immunohistochemical stain, analysis was performed using the 0 and 255 arbitrary unit inversion scale and normalization for square unit of vascular tissue sampled. The average value of intensity in control samples was then normalized to 1, to which all other samples were compared. A minimum of 10 resistance pulmonary arteries (<100 µm external diameter; confirmed by non-association with bronchial airways) were analyzed per animal, and a minimum of 5 small pulmonary arteries (<200 µm external diameter; confirmed by non-association with bronchial airways) were analyzed per patient.

Generation of siRNA:Staramine-mPEG nanocomplexes

To generate siRNA:Staramine-mPEG nanocomplexes, chloroform solutions of 10:1 molar mixtures of Staramine and Staramine-mPEG515 were rotary-evaporated to a film, which was

then held under high vacuum overnight. Water for injection (B. Braun Medical *Inc*., S9200-SS, Bethlehem, PA) was added to the film to give the desired Staramine-mPEG concentration and bath sonicated for ~30 minutes using a Branson Water Bath Sonicator 2510. This process was followed by probe sonication for 5 minutes with a continuous pulse output wattage of 5-10 watts (rms) using a Sonic Dismembrator (Fisher Scientific). The liposome solution was filtered through a 0.2 µm filter, diluted with 5% dextrose to generate an isotonic solution, then mixed with siControl or siISCU at a 20:1 N:P molar ratio. Particle size and zeta potential measurements were conducted (Brookhaven 90 Plus Particle Size and Zeta Potential Analyzer). Nanocomplex uptake of siRNA was determined by gel retardation assay and Quant-iT RiboGreen RNA assays (Life Technologies). Gel retardation assay was performed with a 1% agarose gel in 1X TAE buffer (100V for 1 hour) using 10% TritonX-100 to release the siRNA from the Staramine-mPEG nanocomplexes. The RiboGreen assay was used to determine the percentage of siRNA complexed with Staramine-mPEG; the fluorescently stained siRNA was measured before and after the disruption of complexes with 10% of Triton X-100.

Generation of anti-miR oligonucleotide:7C1 particles

The polymeric nanoparticle 7C1, composed of low molecular weight polyamines and lipids (Dahlman et al, 2014), was utilized for endothelial-specific delivery of anti-miR oligonucleotides in mice. Briefly, and as previously described (Dahlman et al, 2014), 7C1 was synthesized by reacting C_{15} epoxide-terminated lipids with low molecular weight polyethyleneimine (PEI with MW ~ 600 Da) at a 14:1 molar ratio. 7C1 nanoparticles were then mixed with $C_{14}PEG_{2000}$ and anti-miR oligonucleotides in a microfluidic device to produce nanoparticles (diameter between 35 and 60 nm) that were stable in PBS solution for at least 40 days. Particles were dialyzed

against 1X PBS before sterile filtration through a 0.22 um membrane. Particle size and structure were analyzed by dynamic light scattering (DLS) (Zetapals, Brookhaven Instruments). Oligonucleotide concentration in nanoparticles was measured using Quant-iT™ RiboGreen (Life Technologies).

Complex I activity assay

Lung tissues were homogenized and lysed in 1% (v/v) lauryl maltoside (Abcam) in sterile PBS (pH7.4, Sigma-Aldrich), followed by measurement of protein in lysates by DC protein assay (Biorad). 100µg lung protein per animal was loaded into each well for Complex I immunocapture, per manufacturer's instructions (Mitosciences). Immunocaptured Complex I protein concentration was quantitatively determined in each sample by DC protein assay for calculation of specific activity. Separately, after washing, Complex I activity was measured by spectrophotometry (A_{340nm}) reflecting oxidation of NADH to NAD^+.

Transfection of cultured cells and Fe-S fluorescent sensor quantification

Lentiviruses (N-terminal and C-terminal GRX2 constructs mixed in 1:1 v/v ratio; N-terminal and C-terminal GCN4 constructs mixed in 1:1 v/v ratio; and empty lentivirus) were generated (see above) and used for infection of cultured human PAECs in the presence of 2ug/ml polybrene (Santa Cruz Biotechnology). After 24 hours, viruses were removed, human PAECs were washed with PBS, and EGM-2 media (Lonza) was replaced. As described above, virus-transduced human PAECs were treated with hypoxia or normoxia for 48 hours. Alternatively, human PAECs were transfected with a mixture of siRNAs specific for human ISCU1/2 (20nM siISCU1/2, active strands including 5'-GCAUGUGGUGACGUAAUGATT-3', 5'-

CUAUGAGAUACGCACAAUATT-3’, 5’-GAAUGCCUUGGUCUGAAUATT-3’, Santa Cruz Biotechnology) versus control siRNA (20nM siControl scramble. Santa Cruz Biotechnology). Separately, to force expression of miR-210, miR-210 mirVana mimic oligonucleotides (4 nM, Life Technologies) versus mirVana mimic control #1 (4 nM) were transfected. Finally, to inhibit miR-210, a hairpin antisense inhibitor of miR-210 (20nM, Thermo Scientific) was transfected, as compared with a scrambled hairpin control inhibitor #1 (20 nM). After 48 hours, human PAECs were harvested by trypsin, and fluorescence was quantified by flow cytometry (FACSCalibur, Becton Dickinson). Specifically, human PAECs transduced with empty lentivirus were measured as a negative control (M1 gate), and cells with increased fluorescence (M2 gate) were identified. In parallel, human PAECs were harvested by trypsin for immunoblotting to confirm GRX2 and GCN4 transgene expression.

Forced expression of miR-210 in the pulmonary vasculature of mice

In the absence of chronic hypoxia but in the presence of SU5416, male C57BL/6 littermate mice (10-12 weeks old, Taconic) were administered 1 nmol mirVana miR-210 mimic (Thermo Fisher Scientific) or 1 nmol mirVana miR-Control #1 (Thermo Fisher Scientific) at 0, 7, 14 and 21 days, in combination with SU5416 (20mg/kg/subcutaneous/every 7 days). Mimics were pre-mixed with Lipofectamine 2000 [5% (v/v) suspended in sterile PBS] to generate miR-containing cationic liposomes, per the manufacturer’s instructions (Life Technologies). Subsequently, in mice under isoflurane anesthesia, oral passage of a sterile 23-gauge blunt needle beyond the posterior oropharynx into the respiratory tract was followed by drop-wise delivery of mimic solution. Successful microRNA mimic lung delivery was confirmed in each recipient mouse by pulmonary aspiration and transient tachypnea. Upon completion of the 28 day experimental

protocol (Fig. S5a), right heart catheterization was performed prior to tissue harvest. This procedure was previously described in detail (Bertero et al, 2014).

Pharmacologic inhibition of miR-210 in the pulmonary vasculature of mice

Pharmacologic inhibition of miR-210 was assessed in a murine model of PH through anti-miR-210 oligonucleotide delivery. As provided by Regulus Therapeutics, the anti-miR-210 inhibitor was a non-toxic, lipid-permeable, high-affinity oligonucleotide complementary to the active site of miR-210, containing a phosphorothioate backbone and modifications (fluoro, methoxyethyl, and bicyclic sugar) at the sugar 2′ position and shown to be highly-stable *in vivo* (Chau et al, 2012).

To prevent the development of PH using these inhibitors ("prevention protocol"), male C57BL/6 wildtype littermate mice (10-12 weeks old, Taconic) were administered tail-vein intravenous doses of anti-miR-210 (20mg/kg) or anti-miR-control (20mg/kg) suspended in phosphate-buffered medium at 0, 7, and 14 days. Beginning at day 7, mice were subjected to 14 additional days of chronic hypoxia (10% O_2) + weekly injections of SU5416 (20mg/kg/subcutaneous). Upon completion of the 21 day experimental protocol (Fig. 5a), right heart catheterization was performed prior to tissue harvest. This procedure was previously described in detail (Bertero et al, 2014).

Alternatively, C57BL/6 wildtype littermate mice (10-12 weeks old, Taconic) were subjected to 11 days of chronic hypoxia (10% O_2) + weekly injections of SU5416 (20mg/kg/subcutaneous) -- a protocol which we previously confirmed induces PH (Bertero et al, 2014). To ameliorate the

severity of this already existing PH using these inhibitors ("reversal protocol"), mice were then concurrently administered tail-vein intravenous doses of anti-miR-210 (20mg/kg) or anti-miR-control (20mg/kg) suspended in phosphate-buffered medium at days 11, 16, and 21. Upon completion of the 22 day experimental protocol (Fig. 5l), right heart catheterization was performed prior to tissue harvest, as above.

Finally, C57BL/6 wildtype littermate mice (10-12 weeks old, Taconic) were subjected to 14 days of chronic hypoxia (10% O_2) in order to induce PH. To ameliorate such already existing PH using these inhibitors delivered specifically to the vascular endothelium ("7C1 reversal protocol"), mice were then administered tail-vein intravenous doses of anti-miR-210 (1 mg/kg) or anti-miR-control (1 mg/kg) formulated in 7C1 (as described above) at days 14, 21, and 26. Upon completion of the 28 day experimental protocol (Fig. 6b), right heart catheterization was performed prior to tissue harvest, as above.

Knockdown of ISCU1/2 in the pulmonary vasculature of mice

Knockdown of ISCU1/2 in the pulmonary vasculature of a mouse model of PH was performed using custom-designed small interfering RNA (siRNA) oligonucleotides specifically targeting ISCU1/2 (siISCU, 5'–UGAAUCUGGCGUCCACAAUCUUCCC–3'; 5'–GGGAAGAUUGU GGACGCCAGAUUCA–3', Stealth siRNA, Life Technologies). Scrambled siRNA (siCont) was used as a control (5'–UUCCUCUCCACGCGCAGUACAUUUA-3'; 5'– UAAAUGUACUGC GCGUGGAGAGGAA–3', Stealth siRNA, Life Technologies). These siRNAs were then complexed with the Staramine-mPEG nanocomplex delivery system (EGEN, *Inc.*), as previously described (Polach et al, 2012). Staramine-mPEG/siRNA complexes were then delivered to the

pulmonary vascular compartment by intravenous tail-vein injection (4 doses of 40μg siRNA every 5 days) of male C57BL/6 littermate mice (10-12 weeks old, Taconic), as described (Polach et al, 2012) (Fig. S5c). At day 7, mice were subjected to 14 days of hypoxia (10% O_2) + weekly injections of SU5416 (20mg/kg/subcutaneous) or normoxia (21% O_2) + weekly injections of SU5416 (20 mg/kg/subcutaneous). Upon completion of the 21 day experimental protocol, transthoracic echocardiography and right heart catheterization were performed prior to tissue harvest.

Advanced cardiopulmonary exercise testing

As previously described in detail (Tolle et al, 2008), pulmonary gas exchange and minute ventilation (V_E) were measured breath-by-breath using a commercially available metabolic cart (Medical Graphics Corporation CPX/D, St. Paul, MN). The pneumotachograph was calibrated using a 3L syringe at five different flow rates, and the zirconia cell O_2 analyzer and single-beam CO_2 analyzer were calibrated with room air and a 5% CO_2/12% O_2 gas. Radial and pulmonary artery catheters (Edwards Scientific, Irvine, CA) were placed using standard techniques, the latter using the internal jugular approach. Systemic and pulmonary artery pressures were measured with HP1290A quartz pressure transducers (Hewlett-Packard Co., Andover, MA). Transducers were interfaced with MT95K2 recorder (Astro-Med Inc., W. Warwick, RI), and mean end-expiratory values were obtained for right atrial (RAP), mPAP, and mean systemic arterial pressure (MAP). Two-ml samples of systemic and pulmonary arterial blood were obtained at rest and during exercise and analyzed at 37 degrees for pO_2, pCO_2, pH (Model 1620; Instrumentation Laboratories, Lexington, MA), hemoglobin concentration [Hb], and O_2 saturation with O_2 content calculated from the latter two (Model 482; Instrumentation Laboratories).

All patients completed a single bout of incremental cycling (Medical Graphics CPE 2000) exercise to exhaustion. Two minutes of rest were followed by two minutes of unloaded cycling. Work was then continuously increased by 6.25-25 Watts per minute based on history of exertional tolerance. MAP and end-expiratory RAP and mPAP were measured continuously. End-expiratory pulmonary capillary wedge pressure (PCWP) was obtained at rest and during each minute of exercise. Central pressures associated with an end-expiratory pleural pressure swing that is greater than 10mmHg were excluded, or in select cases, incremental exercise was replicated with an esophageal balloon in place, and end-expiratory pleural pressures subtracted. Two ml blood samples were simultaneously drawn from the radial and pulmonary arterial catheters during rest and the last 15 seconds of each minute of exercise. At cessation of exercise, patients were asked which of the following symptoms caused them to stop: shortness of breath, leg fatigue or pain, chest pain, alone or in combination.

Ventilatory and pulmonary gas exchange data were averaged for the final thirty seconds of the two-minute rest period and over contiguous 30-second intervals during exercise. Predicted values for VO_2max utilizing age, gender, and height were previously described (Hansen et al, 1984). The ventilatory threshold was determined by the V-slope method (Beaver et al, 1986). V_E/VCO_2 was measured at the ventilatory threshold. Q_t was calculated from the Fick Principle $Q_t = VO_2/(Ca\text{-}vO_2)$. Predicted maximal Q_t was calculated from predicted VO_2max and an assumed arterial-venous O_2 content difference = ([Hb] × 10). PVR was calculated from (mPAP-PCWP)/Q_t. Peak heart rate ≥ 80% of predicted or peak respiratory exchange ratio (RER) ≥ 1.00 were used as indicators of maximum effort.

Supplementary References

Baggish AL, Hale A, Weiner RB, Lewis GD, Systrom D, Wang F, Wang TJ, and Chan SY (2011) Dynamic Regulation of Circulating MicroRNA during Acute Exhaustive Exercise and Sustained Aerobic Exercise Training. J Physiol 589:3983-3994.

Bauer M, Cheng S, Unno K, Lin FC, and Liao R (2013) Regional cardiac dysfunction and dyssynchrony in a murine model of afterload stress. PLoS One 8:e59915.

Beaver WL, Wasserman K, and Whipp BJ (1986) A new method for detecting anaerobic threshold by gas exchange. J Appl Physiol 60:2020-2027.

Bertero T, Lu Y, Annis S, Hale A, Bhat B, Saggar R, Saggar R, Wallace WD, Ross DJ, Vargas SO, Graham BB, Kumar R, Black SM, Fratz S, Fineman JR, West JD, Haley KJ, Waxman AB, Chau BN, Cottrill KA, and Chan SY (2014) Systems-level regulation of microRNA networks by miR-130/301 promotes pulmonary hypertension. J Clin Invest 124:3514-3528.

Chan SY, Zhang YY, Hemann C, Mahoney CE, Zweier JL, and Loscalzo J (2009) MicroRNA-210 controls mitochondrial metabolism during hypoxia by repressing the iron-sulfur cluster assembly proteins ISCU1/2. Cell Metab 10:273-284.

Chau BN, Xin C, Hartner J, Ren S, Castano AP, Linn G, Li J, Tran PT, Kaimal V, Huang X, Chang AN, Li S, Kalra A, Grafals M, Portilla D, MacKenna DA, Orkin SH, and Duffield JS (2012) MicroRNA-21 promotes fibrosis of the kidney by silencing metabolic pathways. Sci Transl Med 4:121ra118.

Ciuclan L, Bonneau O, Hussey M, Duggan N, Holmes AM, Good R, Stringer R, Jones P, Morrell NW, Jarai G, Walker C, Westwick J, and Thomas M (2011) A Novel Murine Model of Severe Pulmonary Arterial Hypertension. Am J Respir Crit Care Med 184: 1171-1182.

Dahlman JE, Barnes C, Khan OF, Thiriot A, Jhunjunwala S, Shaw TE, Xing Y, Sager HB, Sahay G, Speciner L, Bader A, Bogorad RL, Yin H, Racie T, Dong Y, Jiang S, Seedorf D, Dave A, Singh Sandhu K, Webber MJ, Novobrantseva T, Ruda VM, Lytton-Jean AK, Levins CG, Kalish B, Mudge DK, Perez M, Abezgauz L, Dutta P, Smith L, Charisse K, Kieran MW, Fitzgerald K, Nahrendorf M, Danino D, Tuder RM, von Andrian UH, Akinc A, Panigrahy D, Schroeder A, Koteliansky V, Langer R, and Anderson DG (2014) In vivo endothelial siRNA delivery using polymeric nanoparticles with low molecular weight. Nature Nanotechnol 9:648-655.

Graham BB, Chabon J, Gebreab L, Poole J, Debella E, Davis L, Tanaka T, Sanders L, Dropcho N, Bandeira A, Vandivier RW, Champion HC, Butrous G, Wang XJ, Wynn TA, and Tuder RM (2013a) Transforming growth factor-beta signaling promotes pulmonary hypertension caused by Schistosoma mansoni. Circulation 128:1354-1364.

Graham BB, Chabon J, Kumar R, Kolosionek E, Gebreab L, Debella E, Edwards M, Diener K, Shade T, Bifeng G, Bandeira A, Butrous G, Jones K, Geraci M, and Tuder RM (2013b) Protective role of IL-6 in vascular remodeling in Schistosoma pulmonary hypertension. Am J Respir Cell Mol Biol 49:951-959.

Hansen JE, Sue DY, and Wasserman K (1984) Predicted values for clinical exercise testing. Am Rev Respir Dis 129:S49-55.

Hoff KG, Culler SJ, Nguyen PQ, McGuire RM, Silberg JJ, and Smolke CD (2009) In vivo fluorescent detection of Fe-S clusters coordinated by human GRX2. Chem Biol 16:1299-1308.

Manalo D, Rowan A, Lavoie T, Natarajan L, Kelly B, Ye S, Garcia J, and Semenza G (2005) Transcriptional regulation of vascular endothelial cell responses to hypoxia by HIF-1. Blood 105:659-669.

Minamishima YA, Moslehi J, Bardeesy N, Cullen D, Bronson RT, and Kaelin WG, Jr. (2008) Somatic inactivation of the PHD2 prolyl hydroxylase causes polycythemia and congestive heart failure. Blood 111:3236-3244.

Mok Y, Schwierzeck V, Thomas DC, Vigorito E, Rayner TF, Jarvis LB, Prosser HM, Bradley A, Withers DR, Martensson IL, Corcoran LM, Blenkiron C, Miska EA, Lyons PA, and Smith KG (2013) MiR-210 is induced by Oct-2, regulates B cells, and inhibits autoantibody production. J Immunol 191:3037-3048.

Parikh VN, Jin RC, Rabello S, Gulbahce N, White K, Hale A, Cottrill KA, Shaik RS, Waxman AB, Zhang YY, Maron BA, Hartner JC, Fujiwara Y, Orkin SH, Haley KJ, Laszlo-Barabasi A, Loscalzo J, and Chan SY (2012) MicroRNA-21 Integrates Pathogenic Signaling to Control Pulmonary Hypertension: Results of a Network Bioinformatics Approach. Circulation 125:1520-1532.

Polach KJ, Matar M, Rice J, Slobodkin G, Sparks J, Congo R, Rea-Ramsey A, McClure D, Brunhoeber E, Krampert M, Schuster A, Jahn-Hofmann K, John M, Vornlocher HP, Fewell JG, Anwer K, and Geick A (2012) Delivery of siRNA to the mouse lung via a functionalized lipopolyamine. Mol Ther 20:91-100.

Song Y, Coleman L, Shi J, Beppu H, Sato K, Walsh K, Loscalzo J, and Zhang YY (2008) Inflammation, endothelial injury, and persistent pulmonary hypertension in heterozygous BMPR2-mutant mice. Am J Physiol Heart Circ Physiol 295:H677-690.

Steiner MK, Syrkina OL, Kolliputi N, Mark EJ, Hales CA, and Waxman AB (2009) Interleukin-6 overexpression induces pulmonary hypertension. Circ Res 104:236-244, 228p following 244.

Tolle JJ, Waxman AB, Van Horn TL, Pappagianopoulos PP, and Systrom DM (2008) Exercise-induced pulmonary arterial hypertension. Circulation 118:2183-2189.
