# Supplementary material for: Genetic and hypoxic alterations of the microRNA-210-ISCU1/2 axis promote iron–sulfur deficiency and pulmonary hypertension
Source: EMBO Mol Med. 2015 Mar 30;7(6):695–713. doi: 10.15252/emmm.201404511 (PMC4459813; doi:10.15252/emmm.201404511)
Supplement: Supplementary file 21 [file emmm0007-0695-sd21.docx]

**Supplementary Legends**

**Figure S1.** **HIF-1α and HIF-2α were increased in cultured human PAECs after hypoxic exposure and in PECAM+ pulmonary vascular endothelial cells derived from a hypoxia-induced model of PH in mice.** As assessed by RT-qPCR and intracellular staining with flow cytometric quantitation, HIF-1α (**a-b**) and HIF-2α (**d-e**) were increased by hypoxia (0.2% O_2_, 24 h) as compared with normoxia (21% O_2_, 24 h) in human cultured PAECs (N=6/group), *** P<0.0001. As assessed by RT-qPCR, HIF-1α (**c**) and HIF-2α (**f**) were increased in PECAM+ pulmonary vascular endothelial cells derived from PH mice (Hyp+SU5416) as compared with control mice (Norm+SU5416) α (N=6/group), *** P<0.0001. Error bars reflect SEM.

**Figure S2. miR-210 was up-regulated in pulmonary tissue derived from additional mouse models of PH.** (**a**) RVSP was increased in WT mice exposed to Hyp+SU5416 for 4 weeks as compared with Norm+SU5416 (left bars) (N=4/group), *** P<0.0001. RVSP was increased in *VHL* -/- mice as compared with *VHL* +/+ (WT) littermates (middle bars) (N=4/group), *** P<0.0001. RVSP was increased in wildtype mice suffering from chronic *S. mansoni* infection (N=4) as compared with control uninfected littermates (N=5), ** P=0.0012. (right bars). (**b**) As assessed by RT-qPCR, miR-210 was up-regulated in the lungs of mice chronically exposed to 10% O_2_ for three weeks (Hyp) as compared with 21% O_2_ (Norm) (N=5/group). In (**b**), mean expression of miR-210 in control groups was assigned a fold change of 1, to which all samples were compared, *** P<0.0001. Error bars reflect SEM.

**Figure S3. Validation of *in situ* microRNA staining and immunohistochemical staining in fixed, paraffin embedded lung tissue.** (**a**) Comparison of serial lung tissue sections harvested from WT mice exposed to Hyp+SU5416 and stained with standardized concentration of miR-scramble control probe (miR-Cont) (left micrograph) and miR-210 probe (middle micrograph, purple stain). miR-210 was undetectable by *in situ* staining in *miR-210*-/- murine lungs (right micrograph). Murine scale bar 200 µm. (**b**) Comparison of serial non-diseased human lung tissue stained with standardized concentration of a primary IgG control antibody (left micrograph) or primary ISCU1/2 antibody (right micrograph). Human scale bar is 100 µm.

**Figure S4.** **Levels of miR-210 and ISCU1/2 were not altered in aortic tissue of hypoxic mice.** (**a-b**) As assessed by RT-qPCR, RNA levels of ISCU1/2 and miR-210 were not altered in aortic tissue of mice exposed to chronic hypoxia+SU5416 (Hyp+SU5416, N=4) as compared with mice exposed to normoxia+SU5416 (Norm+SU5416, N=5) [NS P=0.9172 for miR-210, NS P=0.2885 for ISCU1/2]. As assessed by immunoblotting (**c**) and gel densitometry (**d**) protein expression of ISCU1/2 was not altered in aortic tissue of mice exposed to Hyp+SU5416 (N=4) as compared with Norm+SU5416 mice (N=5), NS P=0.1589. Error bars reflect SEM.

**Figure S5.** **Isolation of PECAM+ vascular endothelial cells from lungs of mice.** As described in Supplementary Materials and Methods, magnetic bead purification of PECAM+ endothelial cells was performed from a single cell suspension derived from digested mouse lung. The purity of PECAM+ endothelial cells was assessed by staining with FITC-conjugated anti-PECAM antibody and quantifying positively stained cells by flow cytometry. (**a**) Before purification, PECAM+ cells comprised 30% of cells in the whole lung preparation. (**b**) After purification, PECAM+ cells comprised >95% of tested cells.

**Figure S6. miR-210 was increased, and ISCU1/2 was decreased in PECAM-positive cells in mice suffering from hypoxic PH.** (**a**) RT-qPCR revealed an increase of miR-210 in PECAM+ pulmonary vascular endothelial cells isolated from PH mice (Hyp) as compared with control mice (Norm) (N=6/group), *** P<0.0001. (**b**) By flow cytometry, ISCU1/2 expression was decreased in PECAM+ pulmonary vascular endothelial cells isolated from PH mice (Hyp) as compared with control mice (Norm), ** P=0.0024. (**c**-**f**) By flow cytometry, COX10 and SDHD levels were increased in diseased PECAM+ cells [* P=0.0186 for (**c**), ** P=0.0083 for (**d**)], while E2F3 and Ephrin A3 expression were unchanged [NS P=0.4343 for (**e**), NS P=0.9832 for (**f**)]. Error bars reflect SEM.

**Figure S7. Expression of reported miR-210 target genes Ephrin A3 and E2F3 are unchanged in remodeled pulmonary vessels of mice with experimental PH.** (**a**) Immunohistochemical stain and (**b**) *in situ* quantification (IHC) revealed that expression of the miR-210 targets Ephrin A3 and E2F3 were unchanged in the pulmonary vessels of WT mice suffering from PH secondary to exposure to Hyp+SU5416 (black bars) as compared with Norm+SU5416 (white bars) (N=4/group), NS P=0.2645 for Ephrin A3, NS P=0.153 for E2F3. Immunoblot (**c**) and densitometry (**d**) revealed that expression of Ephrin A3 and E2F3 were not changed in WT mice lung after delivery of miR-210 mimic compared with miR-control, NS P=0.3778 for Ephrin A3, NS P=0.1097 for E2F3. In contrast, immunoblot (**e**) and densitometry (**f**) demonstrated that expression of ISCU1/2 was decreased after delivery of miR-210 mimic in mouse lung compared with miR-control (N=5/group), *** P=0.0003. Murine scale bar 50µm. Error bars reflect SEM.

**Figure S8. siRNA knockdown of ISCU1/2 in PAECs (human) and MEFs (mouse).** RT-qPCR demonstrated successful knockdown of ISCU1/2 in cultured human PAECs using a siRNA targeting human *ISCU* (**a**) and in cultured murine embryonic fibroblasts (MEFs) using a siRNA targeting mouse *ISCU* (**b**). N=5/group, *** P<0.0001. Error bars reflect SEM.

**Figure S9.** **Impaired Fe-S cluster integrity was driven by the miR-210-ISCU1/2 axis in hypoxic human pulmonary microvascular endothelial cells (MVECs).** After lentiviral delivery of GCN4 or GRX2 sensor genes to human pulmonary MVECs, cellular fluorescence was measured by flow cytometry. In contrast to consistent fluorescence from GCN4 control sensors, Fe-S dependent GRX2 sensor fluorescence was decreased by hypoxia (**a**) or siRNA knockdown of ISCU1/2 (siISCU) (**b**) as compared with normoxia or control (siCont), respectively (N=5/group) [*** P<0.0001, NS P=0.4669 for (**a**) and *** P<0.0001, NS P=0.2846 for (**b**)]. In contrast to consistent fluorescence from control GCN4 sensors, Fe-S dependent GRX2 sensor fluorescence was decreased by miR-210 mimic (**c**) and rescued by antisense (AS)-miR-210 inhibition under hypoxia (**d**) as compared with miR-control mimic (miR-Cont) or antisense-control (AS-Cont) under hypoxia, respectively (N=5/group) [*** P<0.0001, NS P=0.0761 for (**c**) and *** P<0.0001 NS P=0.1246 for (**d**)]. Error bars reflect SEM.

**Figure S10.** **Mitochondrial DNA levels in pulmonary vascular cell types and lung tissue were increased after exposure to hypoxia, hypoxia+SU5416, ISCU1/2 knockdown, or genetic deficiency of miR-210, while iron levels were unaffected by hypoxia+SU5416.** Mitochondrial DNA levels were increased in cultured human PAECs (**a**) or PASMCs (**b**) exposed to hypoxia (0.2% O_2_, 24h) as compared with normoxia (21% O_2_, 24h) (N=5/group), *** P<0.0001. Similarly, mitochondrial DNA levels were increased in lung tissue derived from mice exposed to chronic hypoxia with SU5416 (Hyp+SU5416) as compared to normoxia with SU5416 (Norm+SU5416) (N=4/group), * P=0.042 (**c**); from mice exposed to serial delivery of a siRNA specific for ISCU1/2 (siISCU) as compared with a scrambled control siRNA (siCont) (N=4/group), * P=0.0134 (**d**); and *miR-210-/-* mice compared with WT mice (**e**) (N=5/group), * P=0.0207. (**f**) As assessed by assay for iron (II+III), total pulmonary iron levels were not significantly altered after 7 or 21 days of hypoxia+SU5416 exposure (N=4/group) [NS P=0.2456 for 7 days, NS P=0.054 for 21 days]. Error bars reflect SEM.

**Figure S11.** **Expression levels of miR-210 in *mmu-miR-210-/-* mice.** RT-qPCR revealed a specific deficiency in miR-210 expression in various tissue homogenates (10 ng total RNA/sample) derived from *miR-210-/-* mice as compared with WT littermates (*miR-210+/+*) exposed to 72 hours of 10% O_2_ (hypoxia) (top graph). In contrast, miR-195 levels were comparable between both groups (bottom graph). Expression levels are represented by average Ct. (N=4/group).

**Figure S12. Mature endothelin-1 expression was regulated by the miR-210-ISCU1/2 axis in cultured human PAECs*.*** ELISA analysis of protein lysates from cultured human PAECs revealed that mature endothelin-1 expression was increased by forced expression of miR-210 mimic (N=4/group), *** P<0.0001 (**a**) and was reversed by miR-210 inhibition (anti-miR-210) in hypoxia (0.2% O_2_ 24h) (N=4/group), *** P=0.0005 (**b**). Error bars reflect SEM.

**Figure S13. Echocardiographic assessment of left ventricular function in mice with experimental PH.** (**a**) By transthoracic echocardiography, no significant differences were observed in left ventricular (LV) ejection fraction (left graph) [NS P=0.4923 for Norm, NS P=0.1027 for Hyp], LV fractional shortening (middle graph) [NS P=0.0553 for Norm, NS P=0.1199 for Hyp], or interventricular septal thickness (right graph, IVSd; mm=millimeters) [NS P=0.5886 for Norm, NS P=0.3141 for Hyp] in WT mice exposed to Hyp+SU5416 or Norm+SU5416 (white bars) as compared with *miR-210 -/-* mice exposed to the same conditions (black bars) (N=5/group). (**b**) No significant differences were observed in left ventricular (LV) ejection fraction (left graph) [NS P=0.3968 for Norm, NS P=0.6250 for Hyp], LV fractional shortening (middle graph) [NS P=0.1064 for Norm, NS P=0.2467 for Hyp], or interventricular septal thickness (right graph, IVSd; mm=millimeters) [NS P=0.8316 for Norm, NS P=0.9867 for Hyp] in WT mice exposed to Hyp+SU5416 or Norm+SU5416 after either control siRNA delivery (siCont, white bars) or siISCU delivery (black bars) (N=5/group). Error bars reflect SEM.

**Figure S14. Intrapharyngeal delivery of miR-210 mimic oligonucleotides is specific to the lung but not other organs.** (**a**) RT-qPCR revealed no significant alterations of miR-210 expression in various tissue after intrapharyngeal delivery of miR-210 mimic oligonucleotides in heart [NS P=0.1522] (**a**), liver [NS P=0.1989] (**b**), or kidney [NS P=0.2002] (**c**) (N=6/group). Error bars reflect SEM.

**Figure S15.** **miR-210 increased** **mitochondrial iron levels in HPAECs, while total cellular iron levels were unaffected.** (**a**) As assessed by assay for iron (II+III), cellular iron levels in human PAECs were not altered by forced miR-210 expression (miR-210 mimic, N=5/group, NS P=0.3404). (**b**) In contrast, mitochondrial iron (II+III) levels were increased after forced miR-210 expression (N=5/group, * P=0.0388). (**c**) Immunoblot for mitochondrial-specific Cytochrome C revealed expression in mitochondrial, but not cytosolic, fractions, thus confirming efficient mitochondrial extraction from total cellular lysates (human PAECs). Error bars reflect SEM.

**Figure S16. miR-210 was increased, and ISCU was decreased in human pulmonary artery smooth muscle cells under hypoxia.** (**a**) RT-qPCR revealed a significant increase of miR-210 and decrease of ISCU1/2 transcript (**b**) in pulmonary artery smooth muscle cells (PASMCs) exposed to hypoxia (Hyp) as compared with normoxia (Norm) (N=5/group), *** P<0.0001. (**c**) Flow cytometry revealed a decrease of ISCU1/2 protein levels in PASMCs during hypoxia as compared with normoxia (N=5/group), *** P<0.0001. Error bars reflect SEM.
