# Supplementary material for: Genetic and hypoxic alterations of the microRNA-210-ISCU1/2 axis promote iron–sulfur deficiency and pulmonary hypertension
Source: EMBO Mol Med. 2015 Mar 30;7(6):695–713. doi: 10.15252/emmm.201404511 (PMC4459813; doi:10.15252/emmm.201404511)
Supplement: Supplementary file 25 [file emmm0007-0695-sd25.pdf]

Genetic and hypoxic alterations of the microRNA-210-ISCU1/2 axis promote iron-sulfur deficiency and pulmonary hypertension

Kevin White, Yu Lu, Sofia Annis, Andrew E. Hale, B. Nelson Chau, James E. Dahlman, Craig Hemann, Alexander R. Opotowsky, Sara O. Vargas, Ivan Rosas, Mark A. Perrella, Juan C. Osorio, Kathleen J. Haley, Brian B. Graham, Rahul Kumar, Rajan Saggar, Rajeev Saggar, W. Dean Wallace, David J. Ross, Omar F. Kahn, Andrew Bader, Bernadette R. Gochuico,Majed Matar, Kevin Polach, Nicolai M. Johannessen, Haydn M. Prosser, Daniel G. Anderson, Robert Langer, Jay L. Zweier, Laurence A. Bindoff, David Systrom, Aaron B. Waxman, Richard C. Jin and Stephen Y. Chan

Corresponding author: Stephen Y. Chan, Brigham and Women's Hospital, and Harvard Medical School

Review timeline:

Submission date:	23 August 2014
Editorial Decision:	07 October 2014
Revision received:	30 January 2015
Editorial Decision:	17 February 2015
Revision received:	20 February 2015
Accepted:	23 February 2015

Transaction Report:

(Note: With the exception of the correction of typographical or spelling errors that could be a source of ambiguity, letters and reports are not edited. The original formatting of letters and referee reports may not be reflected in this compilation.)

Editor: Roberto Buccione

1st Editorial Decision 07 October 2014

Thank you for the submission of your manuscript to EMBO Molecular Medicine. We are very sorry that it has taken so long to get back to you on your manuscript.

In this case we experienced unusual difficulties in securing three willing and appropriate reviewers. As a further delay cannot be justified I have decided to proceed based on the two available consistent evaluations and sought advice from an external advisor.

Both Reviewers are largely positive although they raise a number of issues, in part overlapping, that require your action. I will not go into any detail as their comments are detailed, reasonable and quite clear and should be thus all fully addressed.

While publication of the paper cannot be considered at this stage, and in agreement with the external advisor, we would be pleased to consider a revised submission, with the understanding that the

Reviewers' concerns must be addressed including with additional experimental data where appropriate and that acceptance of the manuscript will entail a second round of review.

Please note that it is EMBO Molecular Medicine policy to allow a single round of revision only and that, therefore, acceptance or rejection of the manuscript will depend on the completeness of your responses included in the next, final version of the manuscript.

As you know, EMBO Molecular Medicine has a "scooping protection" policy, whereby similar findings that are published by others during review or revision are not a criterion for rejection. However, I do ask you to get in touch with us after three months if you have not completed your revision, to update us on the status. Please also contact us as soon as possible if similar work is published elsewhere.

I look forward to seeing a revised form of your manuscript as soon as possible.

***** Reviewer's comments *****

Referee #1 (Comments on Novelty/Model System):

The authors discovered that deficiency of ISCU1/2, a core protein involved in Fe-S cluster assembly, promoted pulmonary hypertension in mice. They use a myriad of elegant genetic and biochemical approaches as well as a study in a human subject with ISCU deficiency to validate their conclusions. I anticipate that this study will have high impact in the pulmonary hypertension, Fe-S cluster and mitochondrial metabolism field. It is also possible that this work could lead to potential therapeutics for pulmonary hypertension.

Referee #1 (Remarks):

This interesting manuscript from the Chen laboratory (White et al.) shows that chronic activation of miR-210-ISCU1/2 axis in mice promotes pulmonary hypertension (PH). The data presented are an extension of a previous study (Chen, et al.) showing that miR-210 repression of ISCU1/2 leads to Fe-S cluster deficiency, reduced mitochondrial respiration and increased glycolysis. In this current manuscript, their conclusion that alterations in miR-210-ISCU1/2 promote Fe-S deficiency and PH is validated by several elegant approaches using cultured cells, mice and humans. They show that overexpression of miR-210 in lungs of mice leads to increased endothelin 1, right ventricular systolic pressure (RVSP), vessel muscularization and vessel wall thickness, all features of PH, while depletion of miR-210 either genetically (miR-210 KO mice) or pharmacologically (anti-sense oligonucleotides) protected against PH or improved existing PH. They further showed that ISCU1/2 depletion alone in the pulmonary vasculature of mice promoted PH and that a patient with an ISCU mutation displayed exercise-induced pulmonary dysfunction, which was not previously recognized. Elegant biochemical approaches using a fluorescent-based detection assay and EPR spectroscopy was used to confirm a reduction in Fe-S clusters in endothelial cells and diseased pulmonary vasculature in mice and humans. The work is of high quality and the use of multiple approaches convincingly demonstrates the importance of miR-210-ISCU1/2 axis in promoting PD. I believe that these studies will be of particular interest to researchers in the PH, Fe-S cluster and mitochondrial metabolism fields and also to researchers interested in therapeutic interventions for PH.

The work has high potential impact and is suitable for publication in EMBO Molecular Medicine. There are a few issues noted below that could improve the manuscript. Some are minor editorial issues. One issue is clarification of the Discussion. Some sentences are not interpretable and should be rewritten (indicated below). Also, a more in depth, but concise discussion of mechanistic issues related to specific regulation of ISCU1/2 by miR-210 and metabolic dysfunction in diseases linked to mitochondrial Fe-S cluster biogenesis would be useful.

1. What is the significance of extracellular miR-210?

2. ISCU1 and ISCU2 - provide a brief description of the relevance of the two forms.

3. It is intriguing that miR-210 targets, E2F3 and ephrinA3 are not altered in WT mice exposed to Hyp+Su5416. Is the expression of miR-210 targets COX10 and SDHD altered in PH-mice? Briefly comment in the Discussion on a potential mechanism for miR-210 selective regulation of ISCU1/2.

4. On page 5, correct sentence "In correlation, in situ miRNA staining was performed {using} specific mouse models {VHL-/- and IL6 Tg} ....."

5. Page 7, the sentence "....by forced miR-210 expression (Fig. 1c ...)" should be Fig 2c.

6. Figure 2 legend, correct sentence in (d).

7. Figure 3E, the text states that "3' nitrotyrosine levels ....was significantly reduced in the pulmonary vessels of miR-210-/- mice as compared with WT control following hypoxia + Su5416". Therefore, a bar should be placed across WT lung (Hyp+Su5416) vs mir-210-/- (Hyp +SU5416) and an asterisk indicating significance.

8. Page 16, tricylic should be tricarboxylic acid cycle.

9. Discuss potential mechanisms through which dysregulated mitochondrial respiratory activity and the metabolic shift to glycolysis as a consequence of altered miR-210-ISCU1/2 activity affect vascular cell proliferation and remodeling.

10. Page 17 (Discussion), briefly state how altered iron transport causes PH in mice (reference 32). The statement "Such dysregulation of iron homeostasis is also consistent with our finding of increased oxidative stress during vascular Fe-S deficiency in vivo." However, Fig. S9 shows that total iron pulmonary iron content is not significantly changed in mice treated with hypoxia + SU54167 for 7 or 21 days vs day 0. Therefore, there are no studies in this manuscript showing dysregulated iron homeostasis. Clarify this statement. Diseases linked to mutations in Fe-S cluster assembly core proteins (e.g. frataxin, ISD11) are characterized by increased mitochondrial iron accumulation and alterations in iron homeostasis. While total iron content is not altered in diseased mice, it is possible that subcellular iron content is changed. Is mitochondrial iron increased under conditions where ISCU1/2 expression decreased?

Also, Fig. S9 indicates that iron was measured by an "ELISA assay", but this assay is described in the Methods section as QuantiChromeTM Iron Assay Kit, which is not an ELISA.

11. The data convincingly show that ISUC1/2 deficiency promotes PH. A question arises whether deficiencies in other core Fe-S cluster biogenesis proteins (e.g. frataxin, ISD11, NFS1 or ferrodoxin 2) promote PH. Another related question is whether reduced mitochondrial respiration and increased glycolysis due to causes unrelated to Fe-S cluster biogenesis also promote PH. Briefly comment on these questions in the Discussion.

12. Last sentence of the discussion should be rewritten.

Referee #2 (Comments on Novelty/Model System):

This is a very interesting study by an active group of investigators and physician scientists who have made significant contributions to the field. The study provides some conceptually novel observations that will befit all the investigators in the field. Overall, this is an excellent manuscript; I only have some minor concerns and questions.

Referee #2 (Remarks):

This is a well conducted study demonstrating that miR-210-ISCU1/2 regulates the development of pulmonary hypertension. The authors previously reported that hypoxia up-regulated miR-210 leading to specific mitochondrial and metabolic alterations. In current study, they demonstrated that miR-210-ISCU1/2 axis was activated in both mouse models and human examples of PH. Over-

expression of miR-210 repressed ISCU1/2 and promoted PH. Furthermore, deletions of the miR-210 and antisense inhibition of miR-210 protect against the development of PH. Overall, the experiments are well designed and executed, and the study bears conceptual novelty and is of great interest. The data are clearly presented in the manuscripts. I only have some minor concerns that may need the authors' attention to improve the manuscript.

Minor comments:
1. The study may need more and better discussion/description on the interaction of PA endothelial and smooth muscle cells according to the activated miR-210-ISCU1/2 axis. Induction of miR-210 was also found in whole lungs of mice with chronic hypoxia-induced PH by other investigators and up-regulation of miR-210 in PASMC may inhibit cell apoptosis during hypoxia by repressing E2F3 levels. Does ISCU1/2 expression change in PASMC with increased miR-210 levels in PH mice? Does activated miR-210-ISCU1/2 in endothelial cells contribute to pulmonary vascular remodeling or concentric vascular wall thickening (in addition to the enhanced PA smooth muscle cell proliferation)?

2. Several PH animal models have been used to study the pathogenesis of PH. Hyp-mouse and MCT-rat are most commonly used and well characterized. In this study, the authors found that miR-210-ISCU1/2 was activated in VHL-/-, Hyp+Su5416, IL-6 TG, and S. mansoni infection PH models. Are miR-210-ISCU1/2 activated in Hyp-mouse or MCT rat models?

3. Did you see significantly changes in RV/LV hypertrophy in the miR-210 knockout or antisense inhibited mice treated with Hyp+SU5416?

1st Revision - authors' response 30 January 2015

Response to Reviewers (White *et al.*)

We thank the Reviewers and the Editors for their positive remarks regarding this manuscript. We have taken the comments seriously, and we believe that all concerns have now been addressed comprehensively with new data and revised discussion. Consequently, we believe this manuscript is much improved, and we hope that it is now acceptable for publication.

Reviewer #1

1. What is the significance of extracellular miR-210?

We appreciate the opportunity to clarify this point. There is a growing literature showing that diseased tissues, which express increased levels of specific microRNAs, can release those microRNAs into the extracellular space and into the circulating plasma. These circulating microRNAs have been proposed as unique biomarkers for specific diseases. In addition, we and others have shown that extracellular miR-210 can be taken up by recipient tissue to induce true biological effects beyond the source tissue (Hale et al, 2014). Thus, the fact that PH patients carry increased levels of plasma-based miR-210 is significant as these data could be the basis for the future study of miR-210 as a biomarker for PH. These data also may suggest that diseased pulmonary vasculature may even use circulating miR-210 as a molecular messenger to communicate with other recipient tissues during disease progression. These points are now more clearly stated in the Results (p. 6) and Discussion (p. 22-23).

2. ISCU1 and ISCU2 - provide a brief description of the relevance of the two forms.

The description of these two isoforms of ISCU is now in the Introduction (p. 4).

3. It is intriguing that miR-210 targets, E2F3 and ephrinA3 are not altered in WT mice exposed to Hyp+Su5416. Is the expression of miR-210 targets COX10 and SDHD altered in PH-mice? Briefly comment in the Discussion on a potential mechanism for miR-210 selective regulation of ISCU1/2.

We thank the Reviewer for this suggestion. In addition to our original immunohistochemical data

© EMBO

measuring E2F3 and Ephrin A3 expression in remodeled pulmonary vessels, we have quantified the expression of ISCU1/2, COX10, SDHD, E2F3, and Ephrin A3 (all reported direct targets of miR-210) by flow cytometry in PECAM-positive pulmonary vascular endothelial cells derived from mice suffering from hypoxic PH (chronic hypoxia x 3 weeks) versus normoxic control mice. As displayed in Fig. E6-7 and described in p. 6-7, while miR-210 was up-regulated and ISCU1/2 was down-regulated, these other targets of miR-210 either remain unchanged or even increased in expression, thus emphasizing the unique importance of ISCU1/2 as a canonical miR-210 target gene in this context.

In the Discussion (p. 19), we now offer a more in-depth explanation for these findings. Similar to our previous findings for miR-21 and PH (Parikh et al, 2012), this is not an uncommon scenario in miRNA biology where, with increased specific miRNA expression (such as miR-210), only a subset of its validated gene targets may display a net down-regulation. We previously have postulated that specific stoichiometry of miRNA to target transcript levels can dictate the overall effectiveness of target gene down-regulation (either translational repression or mRNA degradation). Alternatively, indirect regulatory pathways may certainly exist (such as during hypoxic stress) that ultimately may be more powerful stimuli on a specific target gene than any direct miR-210 engagement of the target transcript itself. Such complex regulation may serve as homeostatic rheostats. Both possibilities may be active in this case, resulting in an apparent context-specific predilection for only certain targets, such as ISCU1/2, to be subject to net down-regulation.

4. On page 5, correct sentence "In correlation, in situ miRNA staining was performed (using) specific mouse models (VHL-/- and IL6 Tg)....."

This sentence has been corrected (p. 5).

5. Page 7, the sentence "....by forced miR-210 expression (Fig. 1c ...)" should be Fig 2c.

This sentence has been corrected (now p. 8).

6. Figure 2 legend, correct sentence in (d).

This sentence has been corrected (p. 41).

7. Figure 3E, the text states that "3' nitrotyrosine levels ....was significantly reduced in the pulmonary vessels of miR-210-/- mice as compared with WT control following hypoxia + Su5416". Therefore, a bar should be placed across WT lung (Hyp+Su5416) vs mir-210-/- (Hyp +SU5416) and an asterisk indicating significance.

These points have been corrected (Fig. 3E).

8. Page 16, tricylic should be tricarboxylic acid cycle.

This term has been corrected (p. 17).

9. Discuss potential mechanisms through which dysregulated mitochondrial respiratory activity and the metabolic shift to glycolysis as a consequence of altered miR-210-ISCU1/2 activity affect vascular cell proliferation and remodeling.

We thank this Reviewer for this suggestion. We have included a discussion of putative mechanisms in the Discussion with references (p.20-21).

10. Page 17 (Discussion), briefly state how altered iron transport causes PH in mice (reference 32). The statement "Such dysregulation of iron homeostasis is also consistent with our finding of increased oxidative stress during vascular Fe-S deficiency in vivo." However, Fig. S9 shows that total iron pulmonary iron content is not significantly changed in mice treated with hypoxia + SU54167 for 7 or 21 days vs day 0. Therefore, there are no studies in this manuscript showing dysregulated iron homeostasis. Clarify this statement. Diseases linked to mutations in Fe-S cluster assembly core proteins (e.g. frataxin, ISD11) are characterized by increased mitochondrial iron accumulation and alterations in iron homeostasis. While total iron content is not altered in diseased

mice, it is possible that subcellular iron content is changed. Is mitochondrial iron increased under conditions where ISCU1/2 expression decreased?

We thank this Reviewer for allowing us to address this confusing point. We have removed the statement "Such dysregulation of iron homeostasis is also consistent with our finding of increased oxidative stress during vascular Fe-S deficiency in vivo." In its place, we have included new data and discussion to clarify these points (Discussion p. 18-19, p. 23).
As this Reviewer suggests, although total iron levels are not altered, we believe that ISCU1/2 modulation can regulate subcellular iron content and is the subject of our ongoing investigation beyond the scope of this manuscript. First, mutations in ISCU1/2 have previously been linked to mitochondrial iron overload, and we now cite those studies in the text (Haller et al, 1991; Mochel et al, 2008). Second, to determine whether miR-210-specific activity mirrors that finding, we generated whole cell lysates and isolated mitochondria from human PAECs over-expressing this miRNA. While iron content in whole cell lysates were not different, mitochondrial iron levels were greater in miR-210-expressing PAECs as compared with control PAECs (now shown in Fig. E16). Furthermore, our unpublished work indicates that miR-210 likely regulates additional gene targets important in iron homeostasis and transport, consistent with prior reports from other groups (Qiao et al, 2013; Yoshioka et al, 2012). In the Discussion (p. 18-19, p. 23), we now clarify that, although our primary focus in this manuscript is the control of Fe-S biogenesis by miR-210, the complex actions of the miR-210-ISCU1/2 axis in iron handling in general may be relevant to PH pathogenesis and may be of particular significance for the known clinical association of iron deficiency and PH. We hope this Reviewer agrees, however, that any additional studies of complex iron transport here would distract from the focus on Fe-S biology in this already expansive manuscript.

Also, Fig. S9 indicates that iron was measured by an "ELISA assay", but this assay is described in the Methods section as QuantiChromeTM Iron Assay Kit, which is not an ELISA.

This sentence has been corrected (now Fig. E10 legend), and we apologize for the error.

11. The data convincingly show that ISUC1/2 deficiency promotes PH. A question arises whether deficiencies in other core Fe-S cluster biogenesis proteins (e.g. frataxin, ISD11, NFS1 or ferrodoxin 2) promote PH. Another related question is whether reduced mitochondrial respiration and increased glycolysis due to causes unrelated to Fe-S cluster biogenesis also promote PH. Briefly comment on these questions in the Discussion.

We thank this Reviewer for this suggestion. We have included these insightful points in the Discussion (p. 20-21, 23).

12. Last sentence of the discussion should be rewritten.

This sentence has been revised and shortened (p.24).

Reviewer #2

Minor comments:

1. The study may need more and better discussion/description on the interaction of PA endothelial and smooth muscle cells according to the activated miR-210-ISCU1/2 axis. Induction of miR-210 was also found in whole lungs of mice with chronic hypoxia-induced PH by other investigators and up-regulation of miR-210 in PASMC may inhibit cell apoptosis during hypoxia by repressing E2F3 levels. Does ISCU1/2 expression change in PASMC with increased miR-210 levels in PH mice? Does activated miR-210-ISCU1/2 in endothelial cells contribute to pulmonary vascular remodeling or concentric vascular wall thickening (in addition to the enhanced PA smooth muscle cell proliferation)?

We thank the Reviewer for giving us a chance to clarify. We now have included data regarding cultured human PASMCs, whereby hypoxia indeed induces miR-210 while decreasing ISCU1/2 expression (Fig. E16, p. 21). Notably, hypoxic induction of miR-210 is more modest in PASMCs than in PAECs, thus reinforcing the idea that miR-210 has particularly pronounced effects on

endothelial cells, as we have discussed in a prior report (Chan et al, 2009).
In vivo, via serial sections of immunohistochemical staining, we found miR-210 up-regulation (Fig. 1c) and ISCU1/2 down-regulation (Fig. 1f) specifically in the a-smooth muscle actin-positive medial layer of diseased and remodeled pulmonary arterioles. Thus, these data demonstrate the activity of the miR-210-ISUC1/2 axis in PASMCs in PH mice.
Notably, however, while miR-210-specific actions in PASMCs may certainly contribute to overall PH manifestations, we reiterate that our findings in this manuscript focused on the endothelial contribution to disease. Notably, we demonstrated a substantial amelioration of histologic pulmonary vascular remodeling with inhibition of miR-210 specifically in endothelial cells (Fig. 6h-i), thus proving the notion that, indeed, miR-210 is crucial in endothelial cells for control of vascular wall thickening. These points are made in the Results (p. 13) and Discussion (p. 20-21).

2. Several PH animal models have been used to study the pathogenesis of PH. Hyp-mouse and MCT-rat are most commonly used and well characterized. In this study, the authors found that miR-210-ISCU1/2 was activated in VHL-/-, Hyp+Su5416, IL-6 TG, and S. mansoni infection PH models. Are miR-210-ISCU1/2 activated in Hyp-mouse or MCT rat models?

We thank this Reviewer for this suggestion. In the revised manuscript, we have added a study of chronically hypoxic mice (normobaric 10% O2 x 3 weeks) that suffer from PH versus normoxic littermate control mice. In this case, we isolated PECAM-positive pulmonary vascular endothelial cells for study. In these cells, miR-210 was up-regulated (as determined by RT-qPCR) while ISCU1/2 expression was down-regulated (as determined by flow cytometry), consistent with the other rodent models of hypoxia-relevant PH. These data are included in Fig. E6a-b and described on p. 6-7.

3. Did you see significantly changes in RV/LV hypertrophy in the miR-210 knockout or antisense inhibited mice treated with Hyp+SU5416?

We thank the Reviewer for the chance to clarify. As compared with miR-210-replete controls, *miR-210-/-* mice displayed a blunted increase of RV/LV+S (Fulton index) under disease versus baseline conditions (expressed as a ratio of RV/LV+S under Hyp+SU5416 vs. Norm+SU5416). These data (Fig. 3j, p. 11) indicated at least partial protection from the RV hypertrophic response and were consistent with the hemodynamic improvements of RVSP. Notably, LV ejection fraction, fractional shortening, and interventricular thickness were not altered in miR-210 knockout mice as compared with controls either with Norm+SU5416 or Hyp+SU5416 (as reported in Fig. E13).

References

Chan SY, Zhang YY, Hemann C, Mahoney CE, Zweier JL, and Loscalzo J (2009) MicroRNA-210 controls mitochondrial metabolism during hypoxia by repressing the iron-sulfur cluster assembly proteins ISCU1/2. Cell Metab 10:273-284.
Hale A, Lee C, Annis S, Min PK, Pande R, Creager MA, Julian CG, Moore LG, Mitsialis SA, Hwang SJ, Kourembanas S, and Chan SY (2014) An Argonaute 2 switch regulates circulating miR-210 to coordinate hypoxic adaptation across cells. Biochim Biophys Acta 1843:2528-2542.
Haller RG, Henriksson KG, Jorfeldt L, Hultman E, Wibom R, Sahlin K, Areskog NH, Gunder M, Ayyad K, Blomqvist CG, and et al. (1991) Deficiency of skeletal muscle succinate dehydrogenase and aconitase. Pathophysiology of exercise in a novel human muscle oxidative defect. J Clin Invest 88:1197-1206.
Mochel F, Knight M, Tong W, Hernandez D, Ayyad K, Taivassalo T, Andersen P, Singleton A, Rouault T, Fischbeck K, and Haller R (2008) Splice mutation in the iron-sulfur cluster scaffold protein ISCU causes myopathy with exercise intolerance. Am J Hum Genet 82:652-660.
Parikh VN, Jin RC, Rabello S, Gulbahce N, White K, Hale A, Cottrill KA, Shaik RS, Waxman AB, Zhang YY, Maron BA, Hartner JC, Fujiwara Y, Orkin SH, Haley KJ, Laszlo-Barabasi A, Loscalzo J, and Chan SY (2012) MicroRNA-21 Integrates Pathogenic Signaling to Control Pulmonary Hypertension: Results of a Network Bioinformatics Approach. Circulation 125:1520-1532.
Qiao A, Khechaduri A, Kannan Mutharasan R, Wu R, Nagpal V, and Ardehali H (2013) MicroRNA-210 decreases heme levels by targeting ferrochelatase in cardiomyocytes. J Am Heart Assoc 2:e000121.
Yoshioka Y, Kosaka N, Ochiya T, and Kato T (2012) Micromanaging Iron Homeostasis: hypoxia-inducible microRNA-210 suppresses iron homeostasis-related proteins. J Biol Chem 287:34110-34119.

© EMBO

2nd Editorial Decision 17 February 2015

Thank you for the submission of your revised manuscript to EMBO Molecular Medicine. We have now received the enclosed reports from the referees that were asked to re-assess it. As you will see the reviewers are now globally supportive and I am pleased to inform you that we will be able to accept your manuscript pending the following final minor amendments:

1) The quality of some images specifically of the blots in Fig.2 is not ideal. Specifically, there is excess contrasting that should be reduced to an acceptable level.
2) Please provide your manuscript in word (.doc) format.
3) We are now encouraging the publication of source data, particularly for electrophoretic gels and blots, with the aim of making primary data more accessible and transparent to the reader. Would you be willing to provide a PDF file per figure that contains the original, uncropped and unprocessed scans of all or at least the key gels used in the manuscript? The PDF files should be labeled with the appropriate figure/panel number, and should have molecular weight markers; further annotation may be useful but is not essential. The PDF files will be published online with the article as supplementary "Source Data" files. If you have any questions regarding this just contact me.

Please submit your revised manuscript as soon as possible so that we can proceed with formal acceptance.

***** Reviewer's comments *****

Referee #1 (Comments on Novelty/Model System):

The authors have responded to the critiques and have made the appropriate changes. The discussion has been revised and provides a more in depth analysis of the data.

Referee #1 (Remarks):

The manuscript is suitable for publication.

Referee #2 (Comments on Novelty/Model System):

This is a very interesting study from an excellent and productive research team. The manuscript provides some novel and important results that may yield more thoughts on developing novel therapeutic approaches for pulmonary vascular disease. The manuscript is well written with high quality of data. I think it is an important paper in the field of pulmonary vascular pathobiology and pulmonary hypertension, and the revised manuscript is now deemed ready for publication in the journal.

Referee #2 (Remarks):

The authors have adequately and appropriately addressed all my concerns.

2nd Revision - authors' response 20 February 2015

We thank the Reviewers and the Editors for their positive remarks regarding this manuscript. We have now made corrections based on the final recommendations.

1) The quality of some images specifically of the blots in Fig.2 is not ideal. Specifically, there is excess contrasting that should be reduced to an acceptable level.

We have now revised the immunoblot images utilizing less contrast in Fig. 2. In a similar manner, we have also improved the quality of the Western blot images in Fig. E7.

2) Please provide your manuscript in word (.doc) format.

We have provided the main text in a .doc format.

3) We are now encouraging the publication of source data, particularly for electrophoretic gels and blots, with the aim of making primary data more accessible and transparent to the reader. Would you be willing to provide a PDF file per figure that contains the original, uncropped and unprocessed scans of all or at least the key gels used in the manuscript? The PDF files should be labeled with the appropriate figure/panel number, and should have molecular weight markers; further annotation may be useful but is not essential. The PDF files will be published online with the article as supplementary "Source Data" files. If you have any questions regarding this just contact me.

We have provided source data for all of our immunoblots in Fig. 2, Fig. E4, Fig. E7, and Fig. E15. Please note that we routinely cut our membranes in order to blot for different sized proteins derived from the same gel. The immunoblots from these original membranes are shown in the source data.
