# Supplementary material for: Genetic and hypoxic alterations of the microRNA-210-ISCU1/2 axis promote iron–sulfur deficiency and pulmonary hypertension
Source: EMBO Mol Med. 2015 Mar 30;7(6):695–713. doi: 10.15252/emmm.201404511 (PMC4459813; doi:10.15252/emmm.201404511)

Fig 2A. FLAG for GRX2

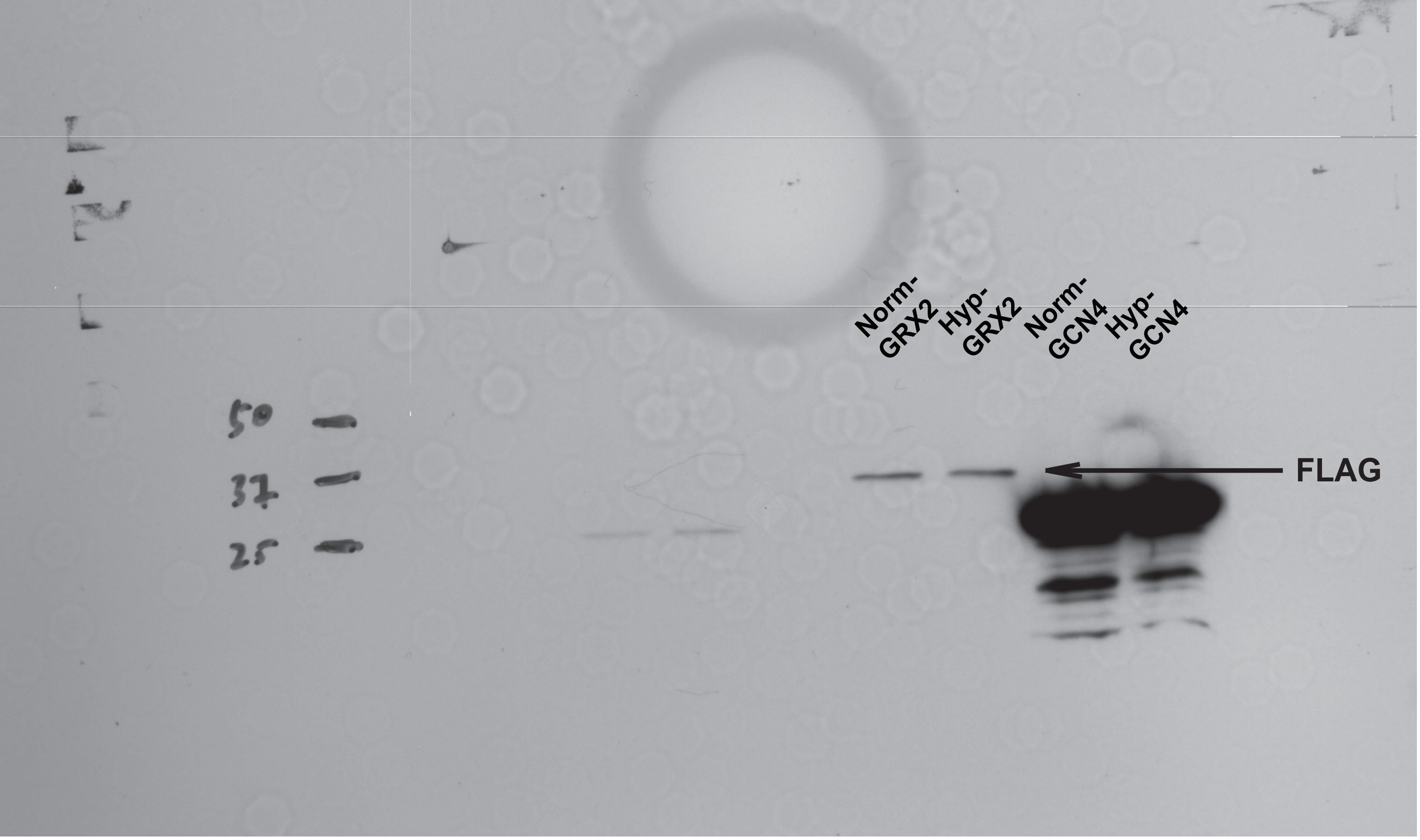

Fig 2A. FLAG for GCN4

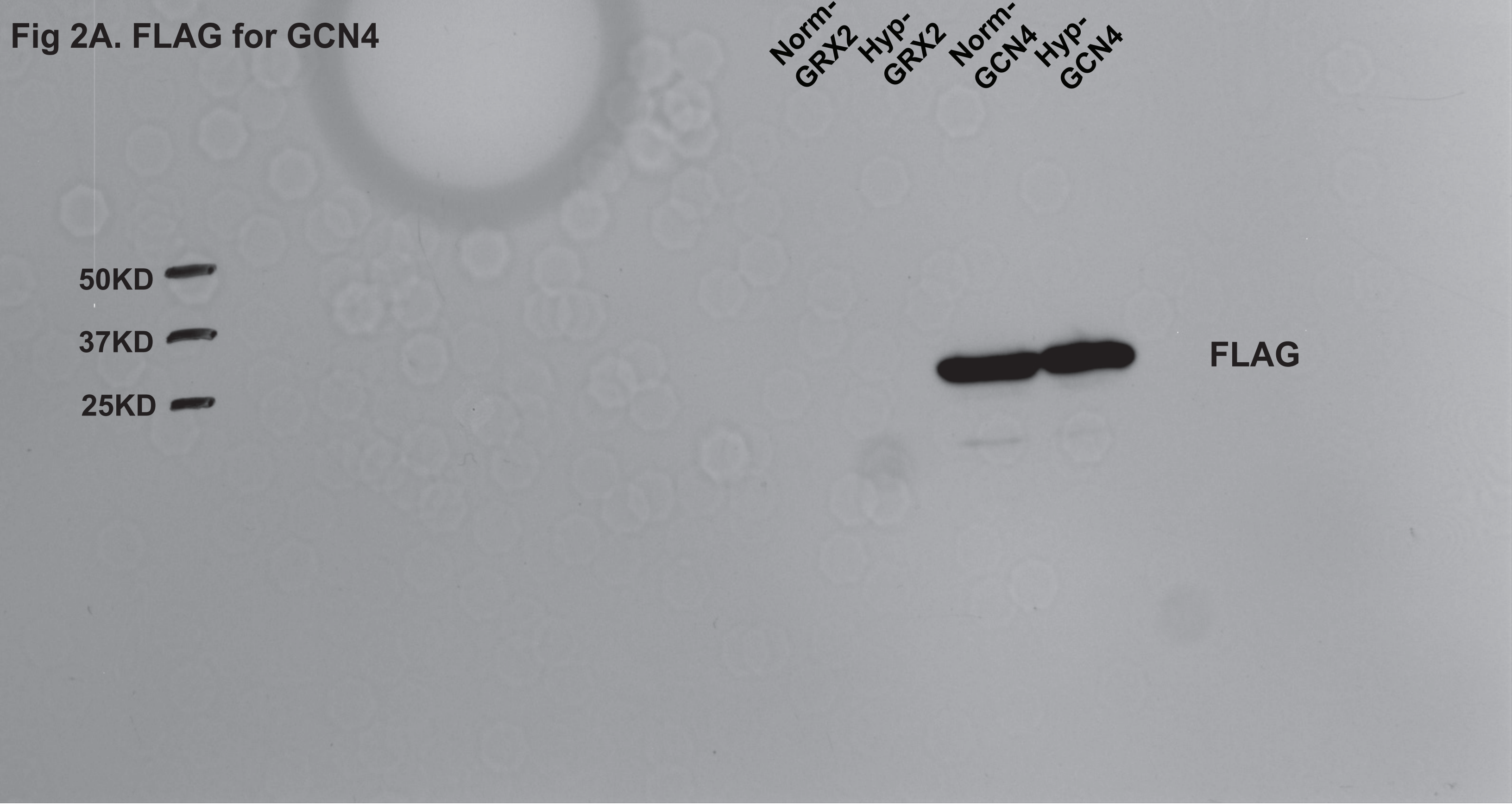

Fig 2A. Stripped membrane for ACTIN

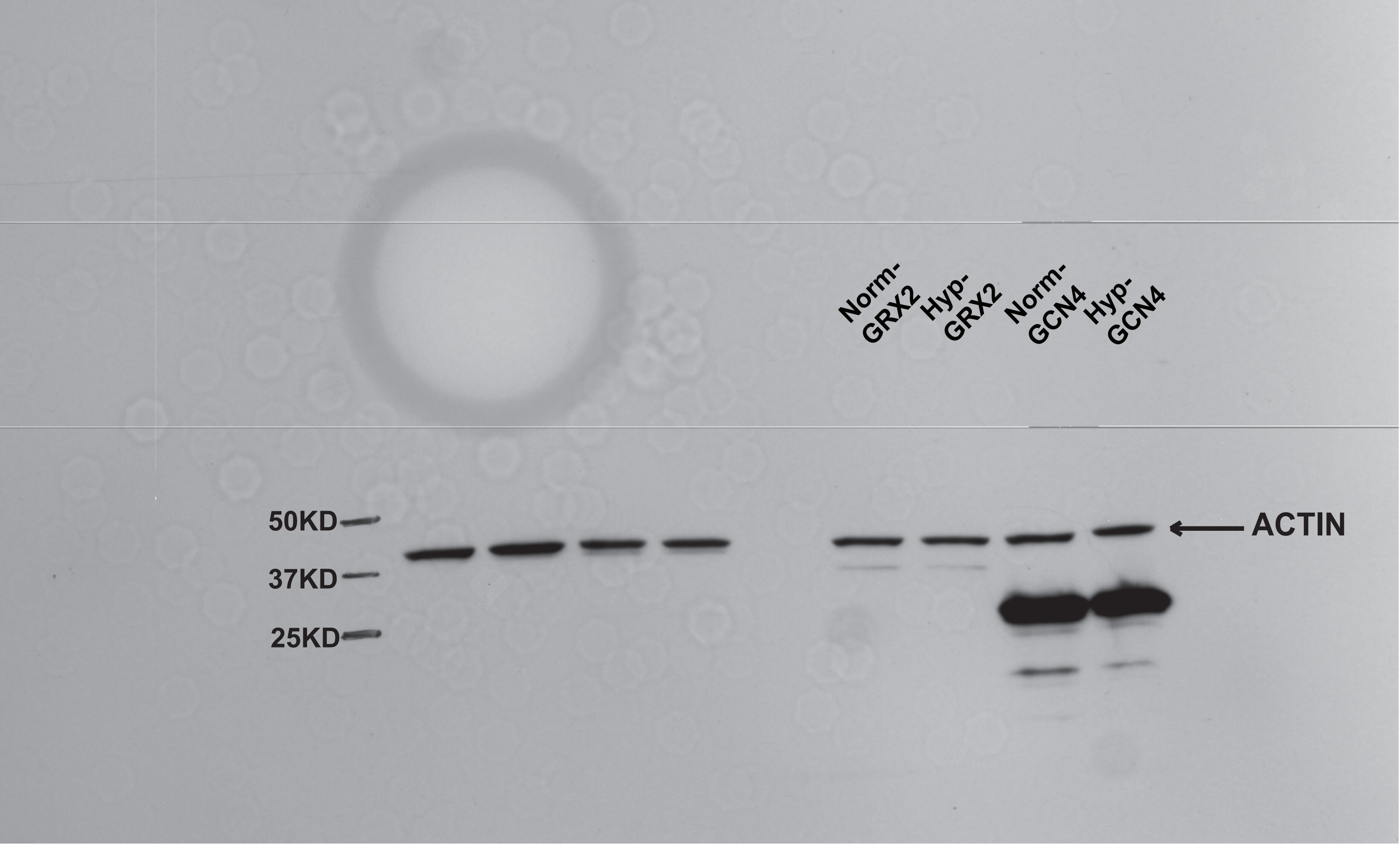

Fig 2B. FLAG for GRX2

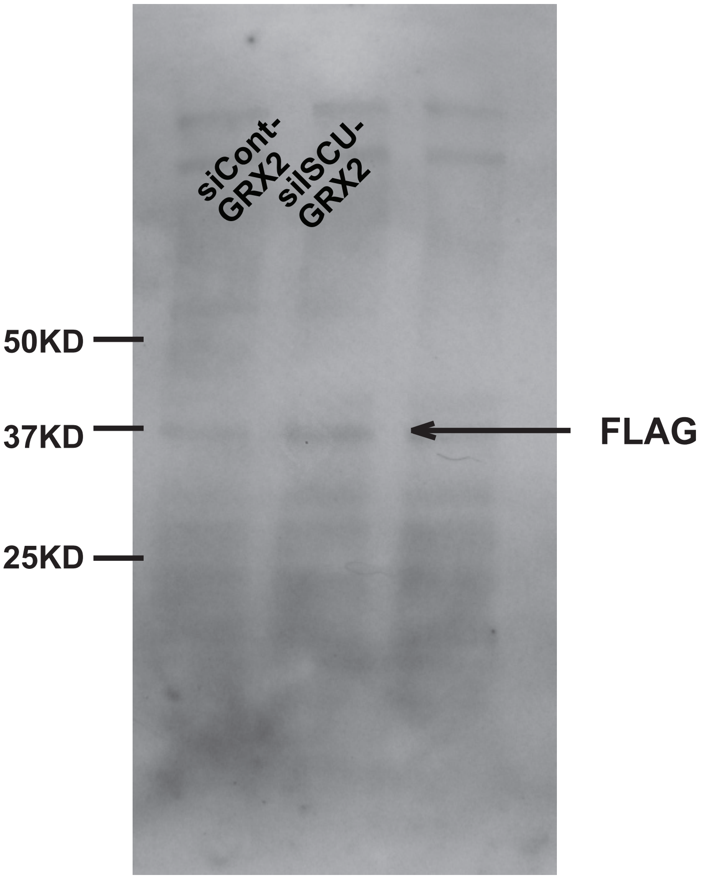

Fig 2B. FLAG for GCB4

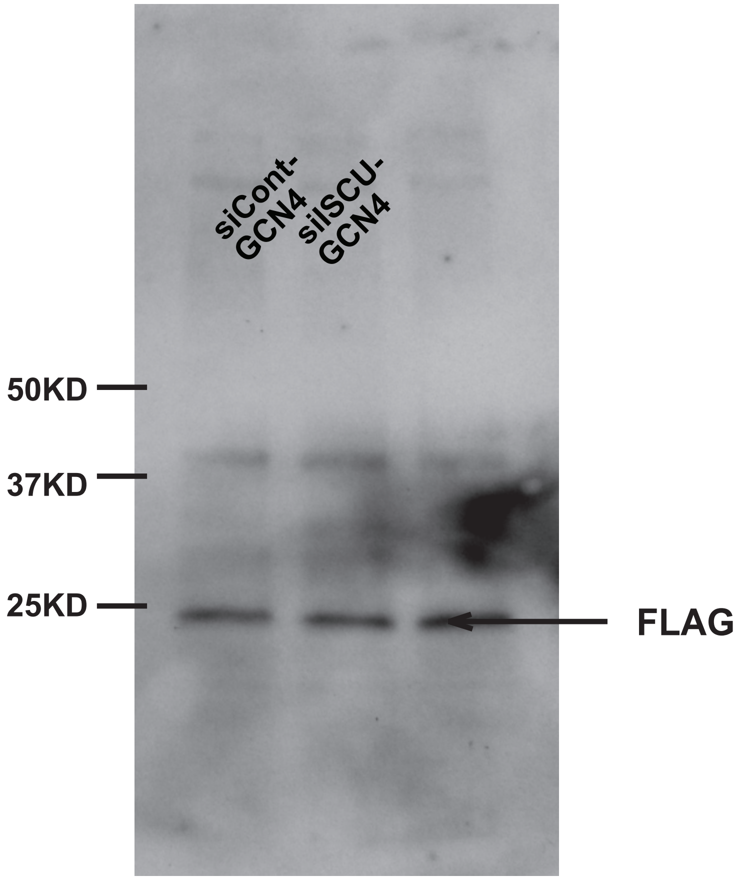

Fig 2B. ACTIN for GRX2

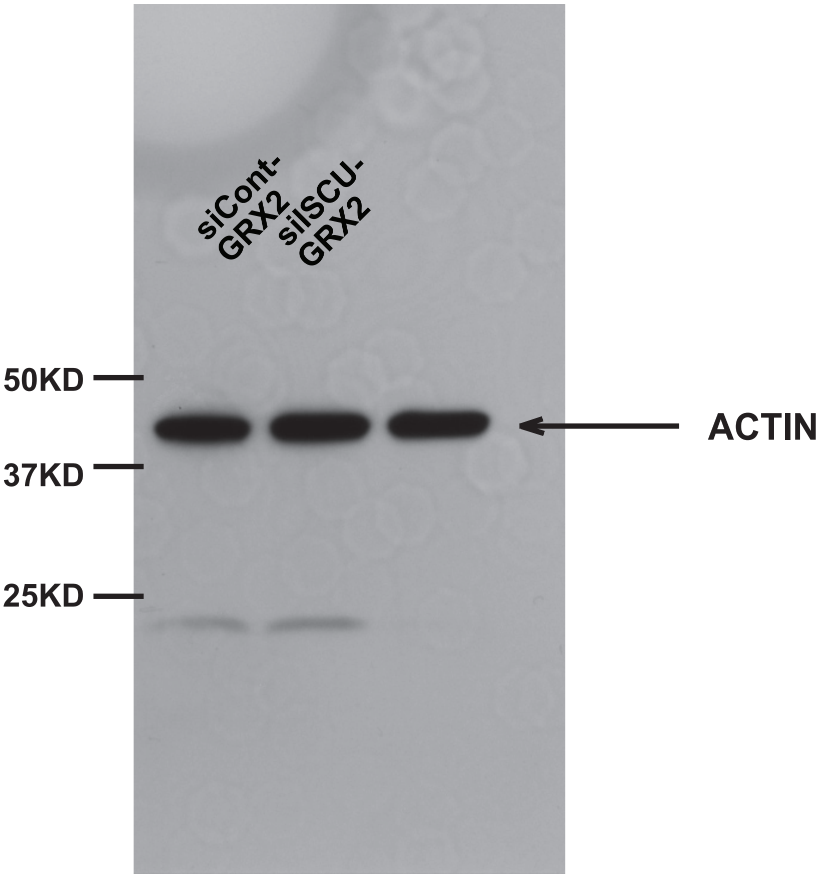

Fig 2B. ACTIN for GCN4

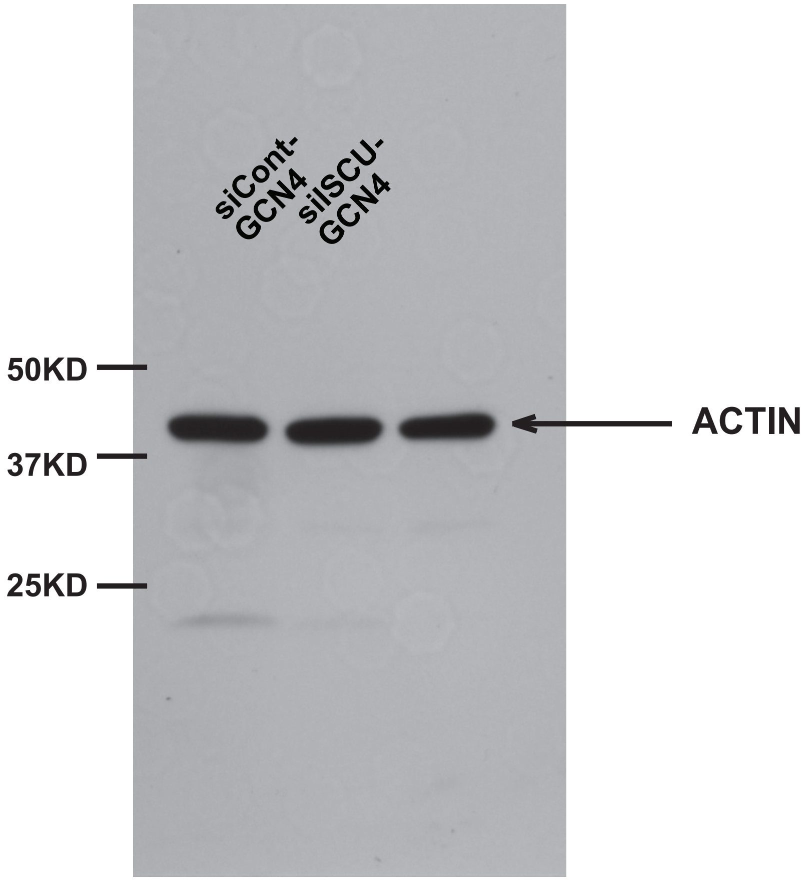

Fig 2C. FLAG for GRX2

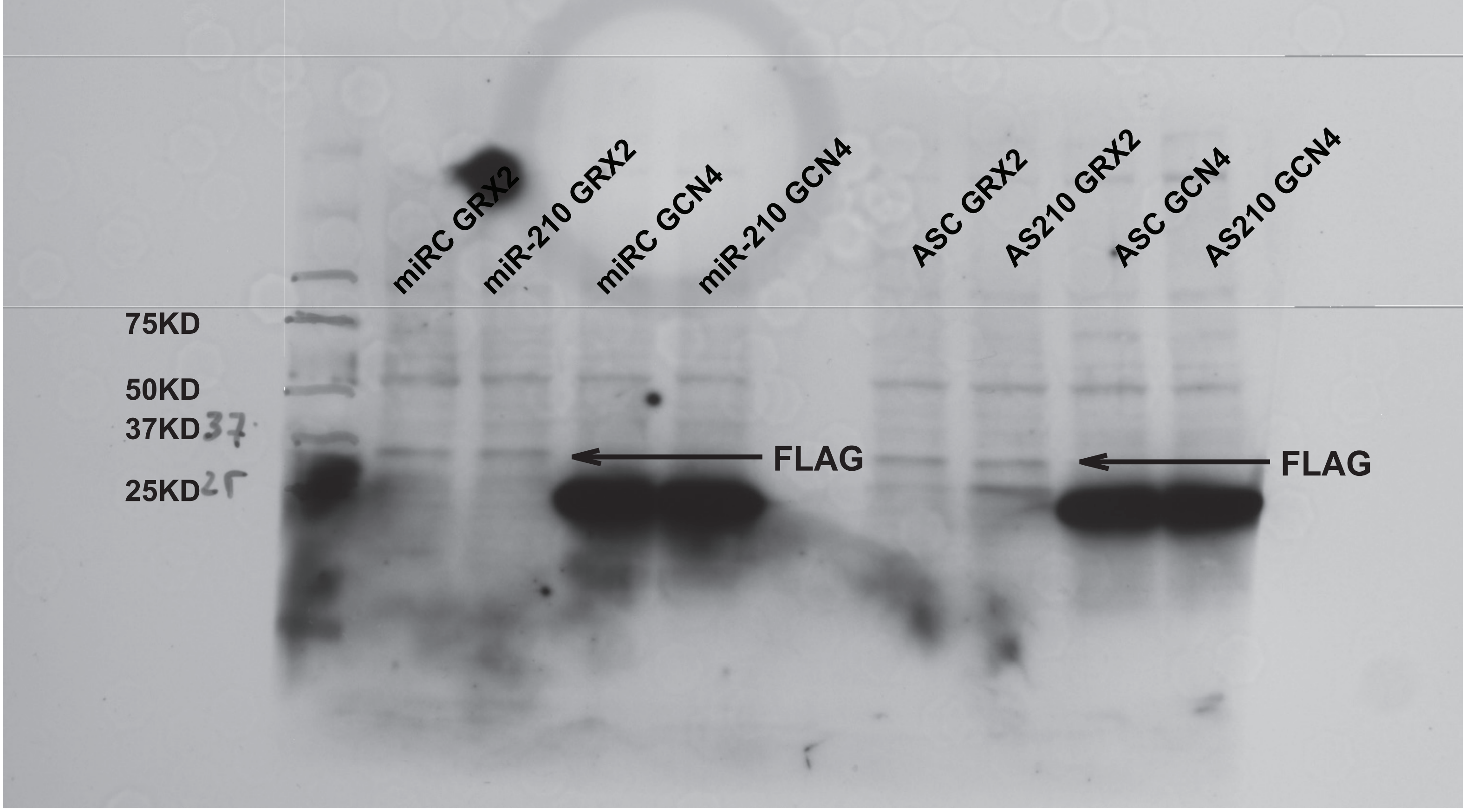

Fig 2C. FLAG for GCN4

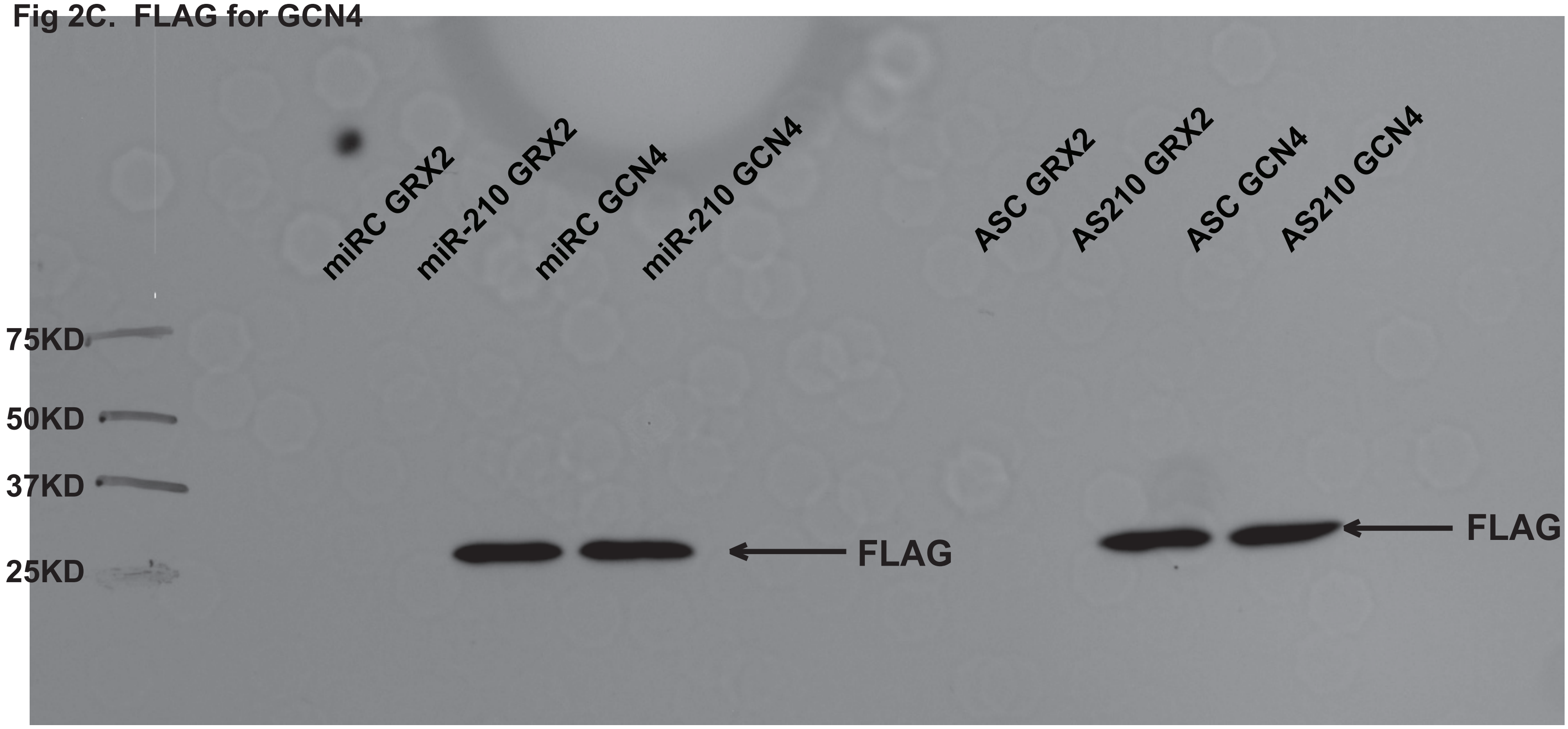

Fig 2C. Stripped membrane ACTIN for GRX2 and GCN4

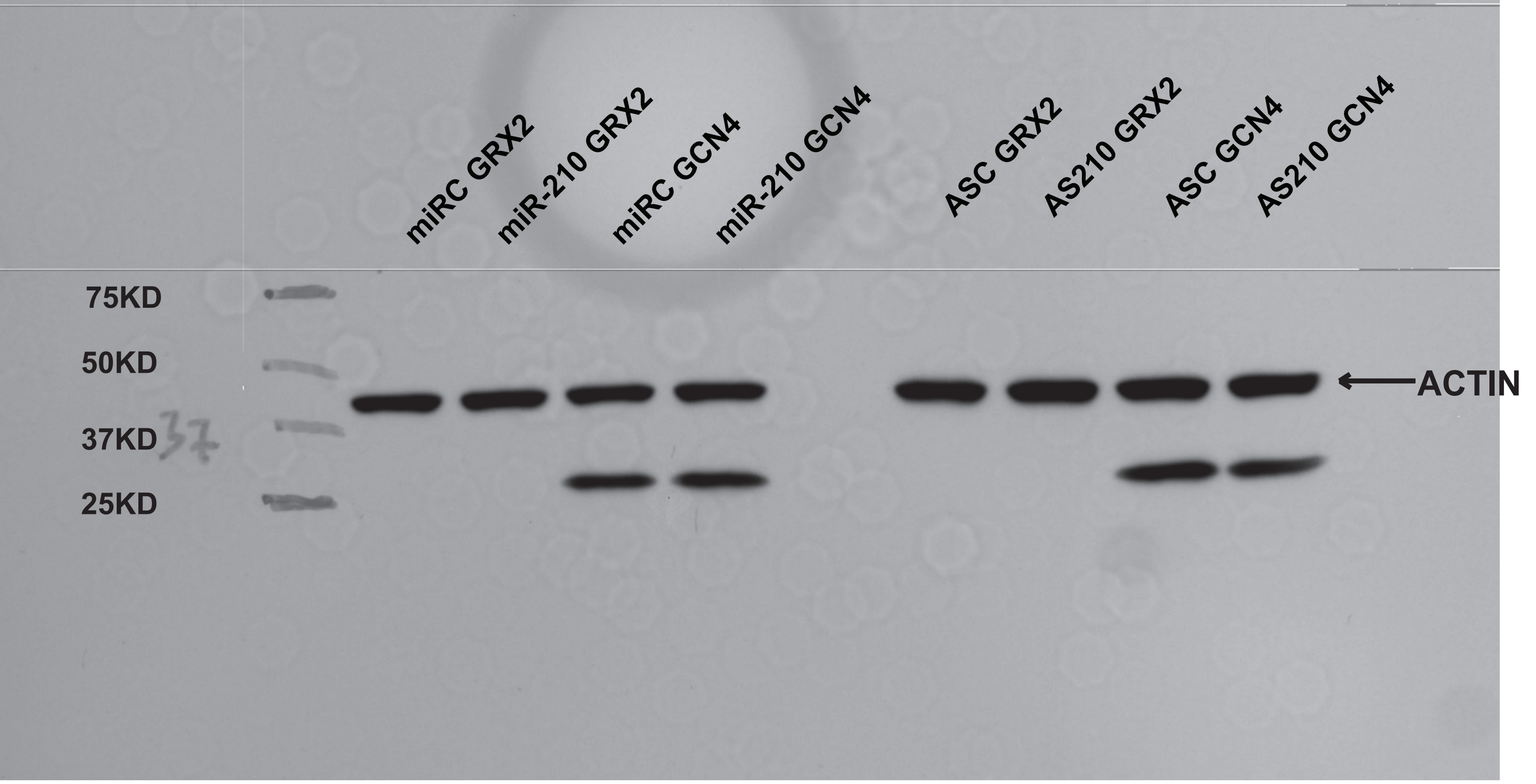

Supplement: Supplementary file 26 [file emmm0007-0695-sd26.pdf]
